# Mycobacteriophage-drived diversification of *Mycobacterium abscessus*

**DOI:** 10.1186/1745-6150-9-19

**Published:** 2014-09-15

**Authors:** Mohamed Sassi, Philippe Gouret, Olivier Chabrol, Pierre Pontarotti, Michel Drancourt

**Affiliations:** 1 Unité de Recherche sur les Maladies Infectieuses et Tropicales Emergentes UMR CNRS 6236 IRD198, IFR48, Institut Méditerranée Infection, Aix Marseille Université, Marseille, France; 2 I2M, UMR 7373, EBM 13331Aix Marseille Université, CNRS, Centrale Marseille, Marseille, France

**Keywords:** *Mycobacterium abscessus*, *Mycobacterium bolletii*, *Mycobacterium massiliense*, Prophages, Mycobacteriophages

## Abstract

**Background:**

*Mycobacterium abscessus* is an emerging opportunistic pathogen which diversity was acknowledged by the recent description of two subspecies accommodating *M. abscessus*, *Mycobacterium bolletii* and *Mycobacterium massiliense* isolates.

**Results:**

Here, genome analysis found 1–8 prophage regions in 47/48 *M. abscessus* genomes ranging from small prophage-like elements to complete prophages. A total of 20,304 viral and phage proteins clustered into 853 orthologous groups. Phylogenomic and phylogenetic analyses based on prophage region homology found three main clusters corresponding to *M. abscessus*, *M. bolletii* and *M. massiliense*. Analysing 135 annotated Tape Measure Proteins found thirteen clusters and four singletons, suggesting that at least 17 mycobacteriophages had infected *M. abscessus* during its evolution. The evolutionary history of phages differed from that of their mycobacterial hosts. In particular, 33 phage-related proteins have been horizontally transferred within *M. abscessus* genomes. They comprise of an integrase, specific mycobacteriophage proteins, hypothetical proteins and DNA replication and metabolism proteins. Gene exchanges, loss and gains which occurred in *M. abscessus* genomes have been driven by several mycobacteriophages.

**Conclusions:**

This analysis of phage-mycobacterium co-evolution suggests that mycobacteriophages are playing a key-role in the on-going diversification of *M. abscessus*.

**Reviewers:**

This article was reviewed by Eric Bapteste, Patrick Forterre and Eugene Koonin.

## Background

*Mycobacterium abscessus* is a non-tuberculous species comprising emerging opportunistic pathogens [1] responsible for sporadic cases and outbreaks of skin and soft-tissue infections following surgical and cosmetic practices [2-4]; catheter-related bacteremia [5,6]; and respiratory tract infections in patients with underlying lung disorders, particularly cystic fibrosis [7-13]. *M. abscessus* is broadly resistant to antibiotics and the cure of localized *M. abscessus* infection may require surgery [14].

Previous phenotypic [3] and genetic [15-17] analyses showed diversity among collections of *M. abscessus* isolates acknowledged by the description of two subspecies, *M. abscessus* subsp. *abscessus* and *M. abscessus* subsp. *bolletii*[18-20]. Later taxon accommodates mycobacteria previously refered as *Mycobacterium bolletii*[21] and *Mycobacterium massiliense*[22]. *M. abscessus* can therefore be viewed as a complex of at least three different organisms *M. abscessus*, *M. bolletii* and *M. massiliense* and this nomenclature will be retained in this paper.

There are a few data regarding mycobacteriophages in *M. abscessus* complex including a 81-kb prophage in the reference *M. abscessus* genome [1]. Also, we recently resolved the electron microscopy 3D structure of a *M. bolletii* mycobacteriophage named Araucaria [23]. However, the repertoire of *M. abscessus* phages and their evolutionary history within this bacterium is unknown and no systematic exploration for prophages and mycobacteriophages has been performed among additionally available sequenced *M. abscessus* genomes, leaving undetermined whether these initial observations were unique to some particular isolates or were representative of the *M. abscessus* species.

Here, exploiting genome sequence available for 48 *M. abscessus* mycobacteria by original bio-informatic analyses, we explored the repertoire of *M. abscessus* mycobacteriophages to gain insights into their evolution history compared to that of *M. abscessus* hosts.

## Methods

### Establishing the repertoire of *M. abscessus* phages

The genomes of 48 *M. abscessus* mycobacteria available in June 2013 were downloaded from Genbank (Table 1, Additional file 1). As for 47 unfinished genomes, the contigs were reoriented based on the *M. abscessus* type strain genome (GenBank GCF_000069185.1) used as reference using MAUVE software [24]. The prophage regions were detected using PHAST software [25]. Protein sequences were predicted in all genomes using prodigal software [26] in order to normalize prediction. *M. abscessus* pan-proteome was annotated using BlastP search with a cutoff E-value < 0.001, percentage similarity > 30% and an alignment length > 50 amino-acids against a home-made database (including PHAST database, Mimivirus, Marseillevirus and additional mycobacteriophage proteins). We further analyzed the *M. abscessus* complex genomes for Clustered Regularly Interspaced Short Palindromic Repeats (CRISPRs) using CRISPRs finder program [27].

**Table 1:** ***M. abscessus *** **genomes properties and prophage regions**

**Group**	**Strain**	**Genome lenght Mb**	**Genome GC%**	**N° of prophage regions**
***M. abscessus***	***M. abscessus*.CIP104536T**	5.09	62.7	1
	M93	5.08	64.2	4
	M94	5.1	64.2	2
	4S-0116-R 4S_0116_R	4.84	64	1
	4S-0116-S 4S_0116_S	4.84	64	1
	4S-0726-RA 4S_0726_RA	4.84	64	1
	4S-0206 M4S_0206	4.86	64	2
	4S-0303 4S_0303	4.86	64	2
	4S-0726-RA 4S_0726_RB	4.86	64	1
	3A-0930-R 3A_0930_R	5.27	64	8
	3A-0119-R 3A_0119_R	5.28	63.8	7
	3A-0810-R M3A_0810_R	5.29	64	8
	3A-0122-R 3A_0122_R	5.23	63.9	5
	3A-0122-S 3A_0122_S	5.23	63.9	6
	3A-0731 3A_0731	5.39	64	8
	3A-0930-R 3A_0930_S	5.25	64	8
	6G-0728-S 6G_0728_S	5.32	64.1	2
	6G-0125-S 6G_0125_S	5.33	64.1	2
	6G-0728-R M6G_0728_R	5.34	64.1	2
	6G-1108 6G_1108	5.34	64.1	2
	6G-0125-R 6G_0125_R	5.14	64.1	2
	6G-0212 M6G_0212	5.14	64.1	2
***M. massiliense***	***M. massiliense *** **BD**	5.2	64.2	3
	M172	5.2	64.2	6
	M47J26	4.87	64.1	3
	M154	4.8	64.1	0
	M18	4.89	64.2	1
	2B-0107 M2B_0107	4.81	64.2	2
	2B-0307 M2B_0307	4.81	64.2	2
	2B-0912-R 2B_0912_R	4.81	64.2	2
	2B-0912-S 2B_0912_S	4.81	64.2	1
	2B-0626 M2B_0626	4.81	64.2	2
	B-1231 M2B_1231	4.81	64.2	2
	1S_51_0915	4.89	64.2	3
	1S-152-0930	4.9	64.2	3
	1S_152_0914	4.9	64.2	3
	5S-1215 5S_1215	5.21	64.1	6
	5S-0421 5S_0421	5.24	64.1	5
	5S-1212 5S_1212	5.24	64.1	6
	5S-0304 5S_0304	5.25	64.1	6
	5S-0708 5S_0708	5.25	64.1	6
	5S-0817 5S_0817	5.25	64.1	6
	5S-0921 M5S_0921	5.25	64.1	6
	5S-0422 5S_0422	5.32	64.1	6
	M159	4.94	64.2	1
	M115	4.98	64.1	3
***M. bolletii***	***M. bolletii *** **BD**^**T**^	5.05	64.2	3
	M24	5.51	64.2	7

### Determining *M. abscessus* phage phylogenies

#### M. abscessus mycobacteria tree

*M. abscessus* genomes were aligned using Muscle aligner implemented in Mauve software [24]. Mauve alignment generated an identity matrix which the identity scores range between 0 and 1, where 0 indicates that no identical homologous nucleotides were found, and 1 indicates that every homologous nucleotide was identical. This matrix was then used to construct *M. abscessus* split network using Neighbor-Net algorithm in the package SplitsTree4 [28].

#### M. abscessus phage tree

The annotated viral and phage proteins were classified using OrthoMCL software [29]. Only protein sequences > 50-amino acid residues were considered for further analyses. Homologous sequences were selected using the all-against-all BlastP algorithm [30] with an E value of <10^−5^. Then, clustering of the orthologous sequences was analyzed using the Markov Cluster algorithm [31]. The inflation index of 1.5 was used to regulate cluster tightness (granularity). The resulting orthologous groups were used to construct a whole-genome network using the Neighbor-Net algorithm based on a gene content matrix. The similarity between two species is defined as the number of phage genes in common divided by the total number of genes of the two species. [32,33]. Using this matrix, we constructed also a heatmap clusterization using R package [http://www.r-project.org/].

#### Detecting gene transfer events

The orthologous groups identified by OrthoMCL were submitted to PhyloPattern for the analysis and manipulation of phylogenetic trees (within the DAGOBAH framework) [34,35]. The *M. abscessus* tree was used as a reference to infer topologies in order to detect gene gain and lost as previously described [35]. The results were submitted to FIGENIX [36] for phylogenetic reconstruction within the DAGOBAH framework as previously described [35]. The output generated by FIGENIX was submitted to the multi-agent system DAGOBAH, in which horizontal gene transfer (HGT) events were detected using an in-house-built transfer filter called HGT agent, as previously described [35,36]. This filter uses PhyloPattern to annotate each internal duplication node of the tree with three tags, including the recipient species, the donor species and external species [34]. Then, it applies a special phyletic pattern and searches the gene tree to find recipient species that are closer to donor species than to other external species that would otherwise be placed between the recipient and donor species in the species tree. In other words, a “donor” subtree must contain only species of a specific group and not those from the “recipient” group and vice versa and there should be no common species between the donor and external groups. Using HGT agent, one can specify the name of the donor and recipient species according to their usage.

## Results

### The repertoire of *M. abscessus* phages

Among 48 analysed genomes of *M. abscessus*, we found that only *M. abscessus* M154 encodes no prophage regions whereas the other 47 *M. abscessus* mycobacteria genomes harbour one to eight prophage regions. A total of 171 predicted prophage regions could be separated into four types i) intact prophages encoding structural proteins, lysis proteins, integration proteins and proteins necessary for replication and recombination ii) questionable prophages iii) incomplete prophage regions iv) small prophage-like elements (Table 1, Additional file 1).

**Figure 1:**
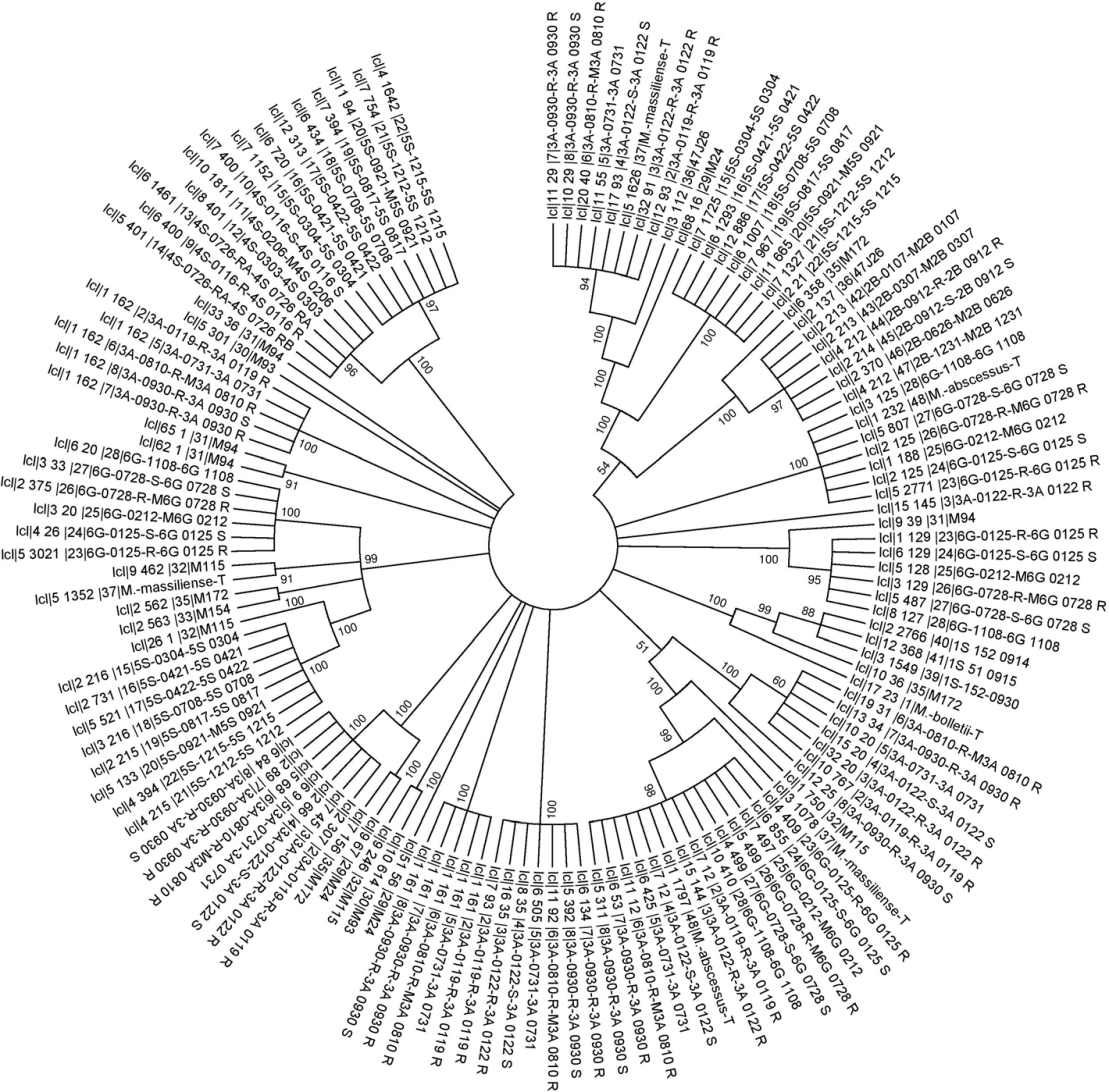
Phylogenetic tree based on annotated tape measure protein sequences using neighbour joining method.

In order to estimate the number of phages infecting *M. abscessus*, a phylogenetic tree was constructed based on Tape Measure Proteins (TMP) (Figure 1). The TMP was selected because it is typically the longest gene in mycobacteriophage genomes and because regions within the TMP gene are conserved [37].We could annotate 135 TMPs which clustered into thirteen groups and four singletons using orthoMCL. The TMP-based phylogenetic tree was constructed using MEGA software. The tree suggested that at least 17 different mycobacteriophages had infected *M. abscessus*, *M. bolletii* and *M. massiliense* during their evolution.

**Figure 2:**
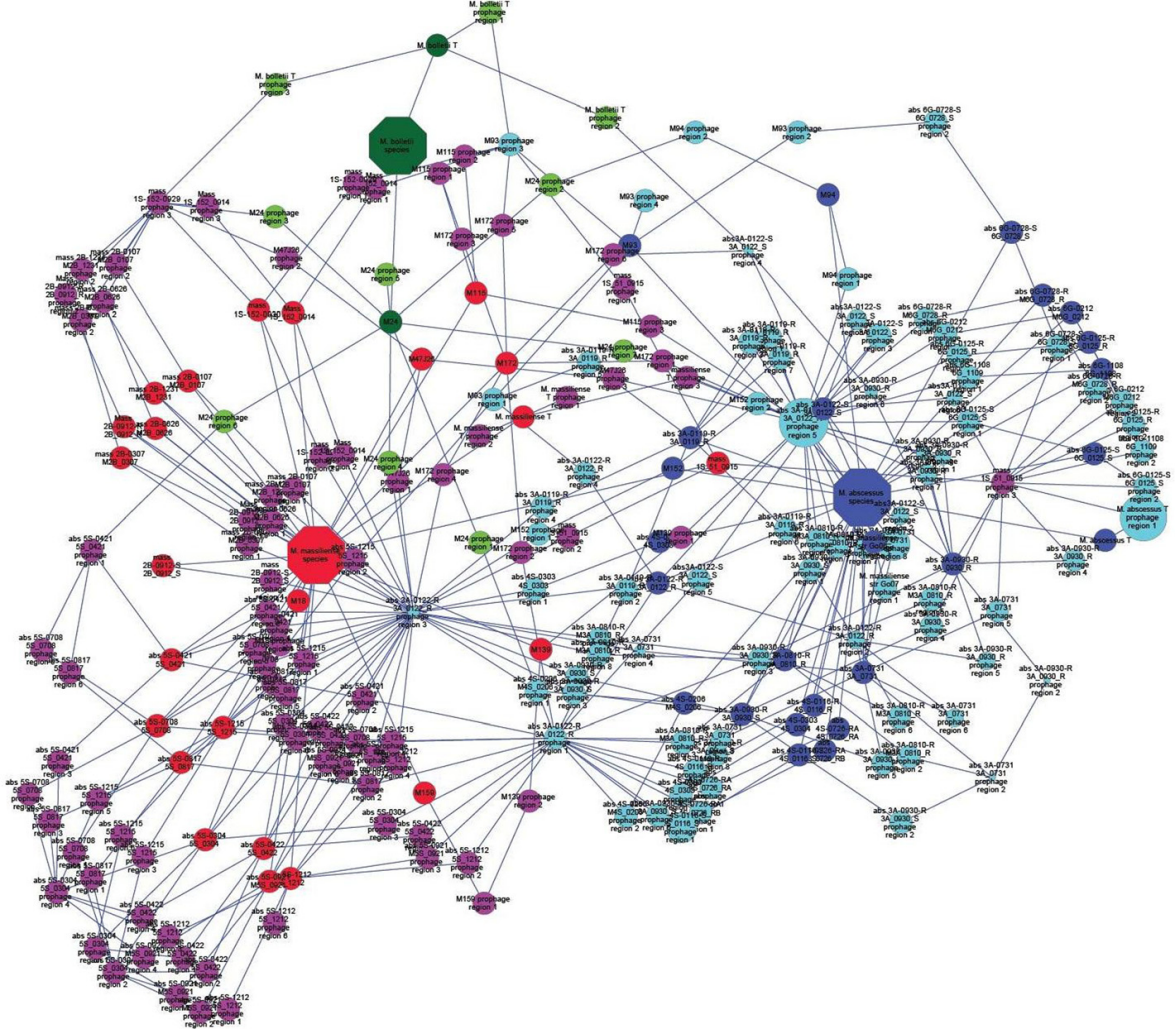
**Interacting Map based on *M. abscessus *prophage region homology.** Blue: *M. abscessus* species. Red: *M. massiliense* species. Green: *M. bolletii* species. The nodes represent the *M. abscessus* prophage regions. The node size correlates to the size of the *M. abscessus* prophage regions. The homologous regions were connected with edges.

**Figure 3:**
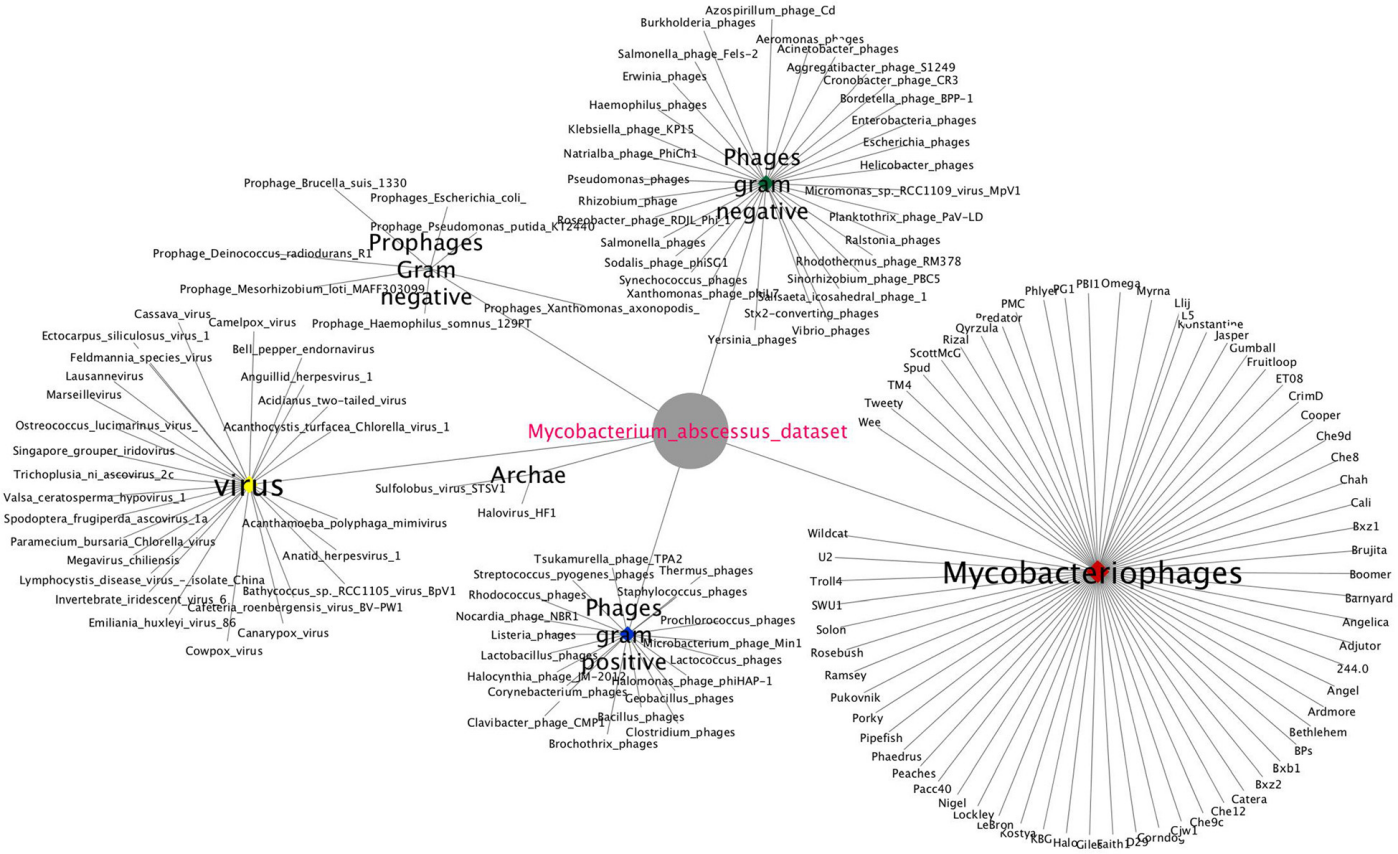
***M. abscessus *** **viral and phage proteins annotation.**

Based on homology between prophage regions of *M. abscessus* genomes, the *M. abscessus* group could be separated into three clusters, *M. abscessus*, *M. massiliense* and *M. bolletii* (Figure 2). Few exceptions were observed: strains M139 and 1S_51_0915 showed prophage region homology with the *M. abscessus* cluster while *M. bolletii* M24 showed prophage region homology with the *M. massiliense* cluster (Figure 2). Also, a 12-kb small prophage-like element is conserved within the *M. abscessus* and *M. massiliense* clusters. Analyzing 242,067 proteins of all 48 *M. abscessus* proteomes found 20,304 (8.4%) proteins homologous to viral or phage proteins represented in Figure 3. These 20,304 proteins yielded 853 groups of orthologous proteins. All the species are represented in 239 groups (28.02%). Only three *M. abscessus* genomes have unique genes, two viral proteins in *M. abscessus* M94, four mycobacteriophage proteins in *M. abscessus* M159 and two viral proteins in *M. abscessus* M172. The annotation of the prophage found 44% proteins to be implicated in DNA replication and bacterial or/and phage metabolism, 37% were annotated as bacteriophage proteins (including structural, integration and terminase) and 14% proteins had no functional annotation. Interestingly, 289 proteins were annotated as holin and 75 as lysin protein. Twenty-five genomes including *M. abscessus*, *M. bolletii* and *M. massiliense* type strains encode endolysin-A and endolysin-B family lysin proteins. A total of 37.3% such proteins are homologous to lysin from mycobacteriophages, the other ones being homologous to lysin from phage infecting Firmicutes bacteria (*Bacillus* phages). Moreover, 156 proteins are repressor and anti-repressor proteins of the lambda repressor CI/C2 family (*Lactobacillus* phage and *Staphylococcus* phage), immunity repressor (*Bacillus* phage and *Geobacillus* phage) and Phage antirepressor protein KilAC domain (*Rhodococcus* phage). *M. bolletii* genome encodes only one CI/C2 repressor homologous to the CI repressor from *Bacillus* phage and one putative repressor located out of the Araucaria genome. All other *M. abscessus* encode three to nine repressors. Only *M. abscessus* 6G and *M. abscessus* type strain encode antirepressors.

Twelve genomes including *M. abscessus* type strain encode no CRISPRs, seven genome including *M. bolletii* type strain encode one possible CRISPR, twelve genomes encode two possible CRISPRs, twelve genomes encode three possible CRISPRs and four genomes encodes four CRISPRs including M154, M115, M172 and M18 strains (Additional file 2).

### Phylogenomic and phylogenetic analyses

#### M. abscessus tree

**Figure 4:**
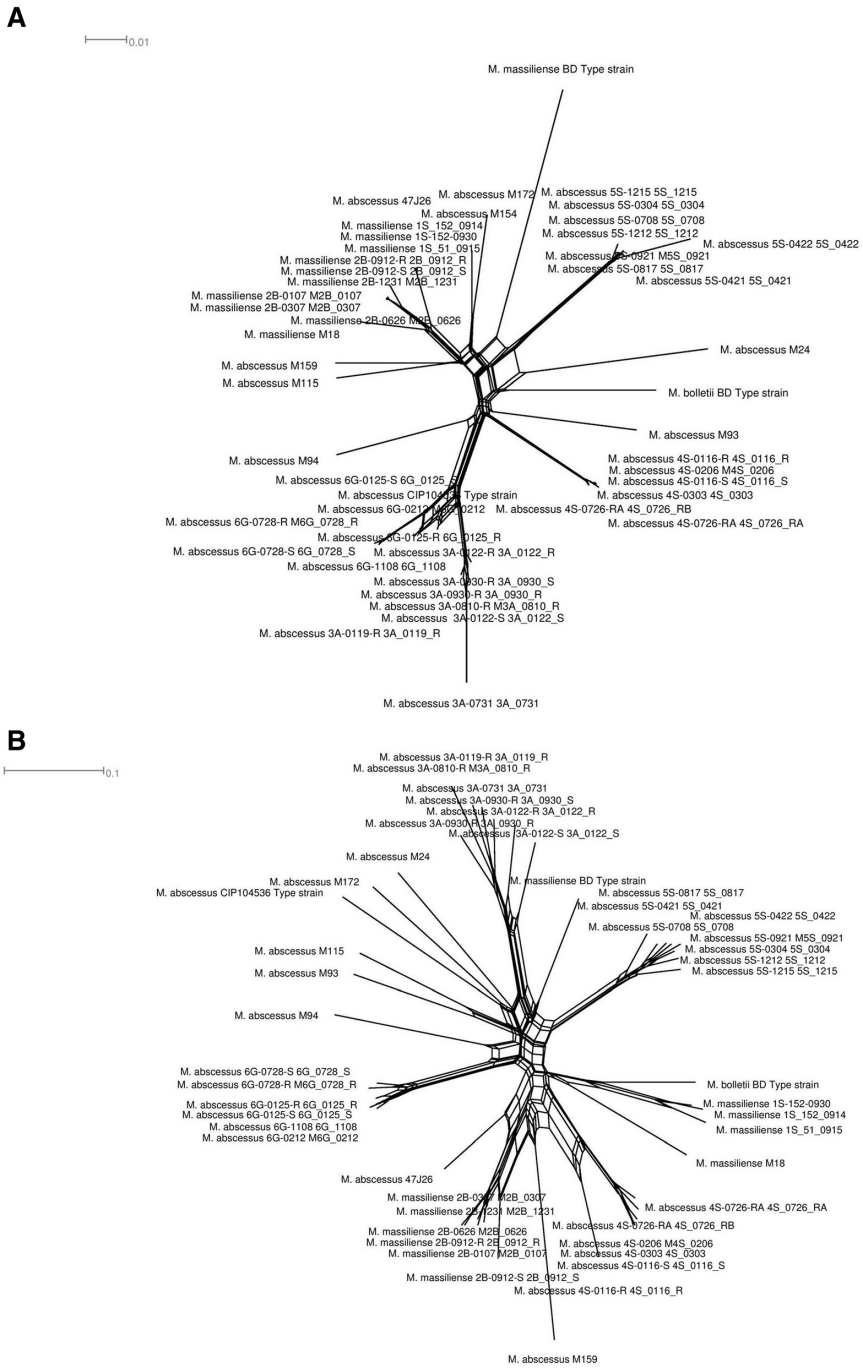
***M. abscessus *phylogeny. A**- *M*. *abscessus* species split network. **B**- *M. abscessus* phage split network based on phage gene content matrix. Every edge is associated with a split of the taxa, but there may be a number of parallel edges associated with each split. The edges separate taxa on one side of the split from the taxa on the other side of the split. The length of an edge in the network is proportional to the weight of the associated split. This is analogous to the length of a branch in a phylogenetic tree.

The split network based on whole-genome content of *M. abscessus* shows the separation of *M. abscessus* strains into three main clusters respectively comprising *M. abscessus*, *M. massiliense* and *M. boll*e*tii* genomes (Figure 4A). *M. abscessus* clusters comprise seven splits of a set of 22 strains. *M. massiliense* cluster comprises two sub-clusters; one sub-cluster forming the strains M159 and M115 and one sub-cluster forming 22 other strains. The *M. massiliense* cluster comprises 12 splits of a set of 24 strains. *M. bolletii* cluster comprises of two strains *M. bolletii* type strain and strain M24.

#### M. abscessus phage tree

**Figure 5:**
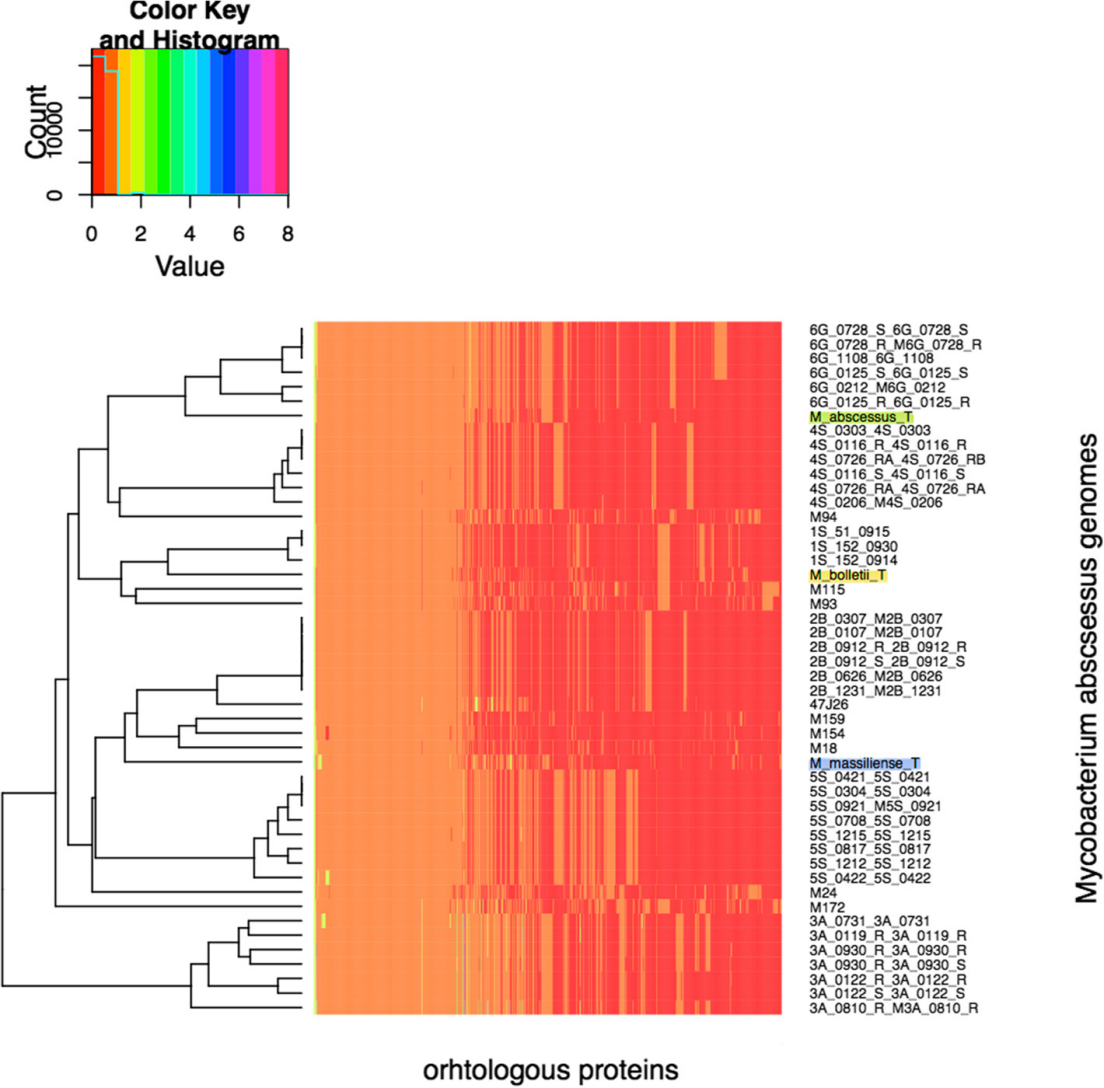
Heatmap clusterization based on protein presence absence matrix.

The split network based on prophage gene content (i.e., the presence or absence of orthologous proteins) showed an organization that differed from that of the *M. abscessus* tree (Figure 4B). The organization of splits in the *M. abscessus* phage split network differs from *M. abscessus* split network. *M. massiliense* type strain clusters with *M. abscessus*, while *M. abscessus* 4S strain clusters with *M. massiliense* strains 2B and M159. The phage split network shows clusterization of *M. massiliense* 1S strains with *M. bolletii* while *M. abscessus* 6G strains forming a different cluster from *M. abscessus*. This phylogenomic analysis showed that *M. abscessus* viral and phage gene repertoires have different evolutionary histories. Also, a heatmap clusterization was constructed using a matrix of presence/absence of orthologous proteins. The heatmap clusterization showed a species organization different from that of the *M. abscessus* tree suggesting that *M. abscessus* may have been infected by several phages during their evolution (Figure 5). Likewise, using the tree based on whole-genome content, individual phylogenetic analysis for the different orthologous proteins groups revealed many topologies that differed from that of the *M. abscessus* tree. These results suggested that gene loss and HGT are relevant for all gene functions. Interestingly, Araucaria TMP clusters with *M. massiliense* strains 1S and M172, suggesting that mycobacteriophages infecting *M. massiliense* mycobacteria may have features similar to Araucaria.

#### Detection of HGT cases

Among the 853 orthologous groups, phylogenetic trees were successfully reconstructed for 213 (25%) of the cases, 156 transfer events (Additional file 3) were detected out of which 33 cases were associated with strong boostrap support for HGT. A 45.45% proportion of the transferred proteins are homologous to mycobacteriophages proteins, 12.12% homologous to viral proteins, 21.21% to proteins of phages infecting gram-positive bacteria and 21.21% to proteins of phages infecting gram-negative bacteria. The probable sources are environmental bacteria in 33 cases, *M. abscessus* in 13 cases, M. *bolletii* in 6 cases and *M. massiliense* in 4 cases.

## Discussion

Analysing 171 prophage regions in 47 *M. abscessus* complex genomes indicated that *M. abscessus* complex has been infected by at least 17 different mycobacteriophages, including Araucaria, the sole available mycobacteriophage that we recently isolated from *M. bolletii*[23]. Noteworthy, *M. abscessus* M154, an isolate from Malaysia [38] is the only strain lacking any evidence for phage. Our previous analysis revealed no unique genes in this strain [39], which nevertheless encodes four possible CRISPRs, one cmr1 family and one cmr4 family, with potential immunity against phage infection [40]. Alternatively, no phage was detected in this strain because of database limitations. For example, Araucaria did not yield significant homology with any other *M. abscessus* complex phage, as confirmed by phylogenetic studies using whole viral and phage proteins clusterization and TMP protein sequence based tree. Sequencing additional mycobacteriophages may next reveal prophage regions in *M. abscessus* M154.

We further observed that some *M. abscessus* phage proteins had homology to other mycobacteriophages and to phages infecting environmental bacteria. *M. abscessus* complex mycobacteria are opportunistic pathogens, but these observations suggest that environments, rather than host microbiota, are sources of evolution for *M. abscessus* complex mycobacteriophages. Noteworthy, potential sources are living in amoeba (Additional file 4) where *M. abscessus* complex mycobacteria including *M. massiliense*[22] and *M. bolletii*[41] are also residing. Amoeba are a place for DNA exchanges between sympatric organisms and the amoeba themselves [42-48]. Data here reported suggest that amoeba are a likely place for mycobacteriophage exchanges and therefore, may be a place to look at for the discovery of new mycobacteriophages.

Accordingly, a striking feature of *M. abscessus* complex phage genomes is their pervasive mosaicism, a previously reported hallmark of mycobacteriophages [49,50]. Our phylogenomic and phylogenetic analyses revealed a different split network topology between the hosts and the phages. This probably reflects reciprocal genome evolution through a dynamic co-evolutionary process [51]. *M. abscessus* complex was infected by at least 17 phages and these infections contributed to differentiate *M. abscessus* complex into several clusters of mycobacteria. Widespread occurrence of phage sequences in almost all studied *M. abscessus* complex isolates suggests that the rate of prophage invasion is faster than the rate of mutation, implying rapid evolution of *M. abscessus*. Also, in *M. abscessus* complex a total of 6/33 (19%) gene transfers occurred between a set of donor species (at least two donors) and a recipient species and 27/33 (81%) gene transfers occurred between a single species donor and a recipient species, clearly indicating it is an on-going process.

## Conclusions

Excluding the prophage-free strain M154, phages account for only 6.7-9.6% of *M. abscessus* complex genomic content, but they profoundly impact their hosts, participating to their on-going diversification.

## Reviewers’ comments

We appreciate the reviewer’s comments from Dr. Eric Bapteste (UPMC, Institut de Biologie Paris Seine, France), Dr. Patrick Forterre (Institut de Génétique Microbiologie, 91405 Orsay Cedex, France Institut Pasteur) and Dr. Eugene Koonin (National Center for Biotechnology Information, National Library of Medicine, National Institutes of Health, Bethesda, MD, USA). We have revised the manuscript according to your comments and suggestions.

### Reviewer 1: Dr. Eric Bapteste (UPMC, Institut de Biologie Paris Seine, France)

The general topic of this research and the reported findings are very relevant for Biology Direct, however the current version of this MS is certainly not yet ready for publication.

Even though I am willing to trust the authors about their main conclusions, I strongly recommend major revisions, because it is currently hardly possible to evaluate most of the evidence on which they based their observations.

In short:

- The trees supporting lateral gene transfers should be presented in an organized fashion in a Supp. Mat.

Authors’ response: *The reviewer is right*, *all 605 trees are now provided as Additional File and trees supporting LGT are highlighted*.

- Many current figures are not of sufficient quality to be printed in a journal (gene/taxon names are impossible to read, etc.)

Authors’ response: *Authors improved the quality of documents*.

- The methodology used for the tree reconstruction is not sufficiently detailed: crucial information such as the number of positions retained or the substitution models used are lacking.

Authors’ response: *Methodology has been expanded* (*Lines 87*–*92*; *lines 100*–*102*; *lines 112*–*122*).

- Many figures presented in the text are under-interpreted, and not critically discussed.

Elements requiring significantly more details:

p.4. l. 86: The authors report that ‘M. abscessus proteomes were aligned using Mauve software [24]’. I am not familiar with ProgressiveMauve, but does this software really align proteomes, or is it rather a tool to align genomes based on their content and gene order? If so, the next sentence in the MS is hard to understand

Authors’ response: *M. abscessus genomes were aligned using Mauve software. Progressive Mauve uses Muscle or clustlW to perform alignment. Here we used Muscle. The authors corrected and explained the methodology* (*P.4 line 86*).

‘Then M. abscessus tree was constructed using Neighbor-Net algorithm in the package SplitsTree’. What distance matrix was provided to Splitstree? What was this distance reflecting?

Authors’ response: *Mauve alignment generates an identity matrix file which calculates the identity score range between 0 and 1*; *0 indicates no homologous nucleotides and 1 indicates that every homologous nucleotide was identical. The authors explained this part in the text* (*P.5 line 88*).

If the proteomes alignments evoked above were ‘classic’ protein alignments, then what happened to poorly aligned sites? How many positions were retained? For how many proteins? The material and methods must be much more detailed for the reader to really understand the analysis and the results. Please note that this criticism also applies for the trees that show some LGT.

Authors’ response: *The authors corrected that M. abscessus genomes was aligned not proteomes in the text* (*P.4 line 86*).

p.5. l, 95–96: The analysis described here faces a potential pitfall if ‘presence’ and ‘absence’ were treated in a symmetric fashion, especially if there were lots of ‘absences’ in this matrix. While ‘presences’ can be used to group genomes sharing some features, more caution is required in the use of shared ‘absences’.

If the groupings of genomes are firstly caused by the lack of shared features, then these groupings can be very artefactual (sharing ‘losses’ is different from sharing nothing. The potential problem here is that two genomes that have positively nothing, or not much in common, may still be grouped due to their lack of genes, while sometimes it is a better idea not to group genomes that share nothing in common!). How did the authors address this potential issue?

Authors’ response: *Here we constructed a matrix based on the similarity between two species which is defined as the number of genes that they have in common divided by their total number of genes* (*P.5 line l00*-*104*).

p.5, l. 98–108: Incongruence between trees is considered to be synonym of lateral gene transfer, and the possibility of tree reconstruction artefacts is not discussed. Since the tree reconstruction methods are poorly described, it is difficult to evaluate this part of the work.

Authors’ response: *The methods of tree reconstruction are now described in the materials and methods section*. (*P.5 line 105*–*124*).

p.6., l. 119: The authors write that ‘a phylogenetic tree was constructed based on Tape Measure Proteins (TMP)’ What are ‘Tape Measure Proteins’? Why this marker? What methods/positions/models were used to reconstruct this tree?

Authors’ response: *The TMP was selected because it is typically the longest gene in mycobacteriophage genomes and because regions within TMP gene are conserved* (*P.6 line 125*).p.7, l. 156. Figure 4A: I have not been able to see this figure, or if it refers to the split network, then its description in the text must be expanded and be more critical. What do the proportion/size/presence of splits indicate? What is their biological meaning? Also for example, what does the position of M. massiliense BD type strain suggest? Same question for the position of M. abscessus 47 J26?

Authors’ response: *The authors performed a better figure quality and more description in the text. The length of an edge in a split network is analogous to the length of a branch in a phylogenetic tree*.p.7, l.160: same problem with Figure 4B.

Authors’ response: *The authors performed a better figure quality and more description in the text* (*P.7 lines 158*–*160*).Overall, the exploitation of these 2 figures is a bit vague. The authors only write about it that: ‘A phylogenomic tree based on prophage gene content (i.e., the presence or absence of orthologous proteins) showed an organization that differed from that of the M. abscessus tree (Figure 4B).’ Please, increase the descriptions of what these differences are (or use a metrics to compare these two split networks).

Authors’ response: *The authors performed a better figure quality and more description in the text* (*P.8 line 186*–*190*).p.8, l.165: Figure 5 is neither described nor exploited in a way that allows to make sense of the main text about it. Please, give more time to a careful critical description of the figure.

Authors’ response: *The authors clarified this point* (*P.8 line 174*).

p.8, l. 172: The 214 phylogenetic trees mentioned here (reconstructed how, please precise) should be logically classified and presented as Supp. Mat, or made available somewhere. Currently, it is simply impossible to review this part of the MS without being able to look at the evidence.

Authors’ response: *The phylogenetic trees are provided in Additional file*4.

p.10. l-210-211: ‘Also, M. abscessus complex phages further shuttled gene transferts, 16/29 (55%) of which occurred between different M. abscessus complex clusters but 13/29 (45%) of which occurred between strains of the same cluster, clearly indicating it is an on-going process’. Where do these numbers come from? How were they obtained? Where is the evidence?

Authors’ response: *The authors clarified this point in the text* (*P.10 line 241*–*246*).p.19, l. 379: Figure 2 legend: What is ‘an interacting map’? How is one supposed to read such a map? What are the nodes? What are the edges? More descriptions are required.

Authors’ response: *The authors clarified this point* (*P.19 line 422*).

There are also some minor typos/issues:

p.3, l. 56 ‘three different organisms’: do you really mean organisms, or species, or strains here?

Authors’ response: *We mean organisms*.p.4, l.86 (and in some other places in the text): the authors refer to the splitsnetwork as the ‘M. abscessus tree’. Elswhere, as in the legend of Figure 4, they call this type of graphs ‘network trees’. This wording is confusing. Is it a network or is it a tree? To me, each of this graph should be called a split network.

Authors’ response: *The authors corrected network tree to split network* (*P.5 line 91*).

p.5., l. 95: Likewise, what the authors call ‘a whole-genome phylogenetic tree’ looks very much like a network.

Authors’ response: *The authors corrected phylogenetic tree to network* (*P.5 line 100*).Figure 2: ‘Few exceptions were observed: strains M139 and 1S_51_0915 showed prophage region homology with the M. abscessus cluster while M. bolletii M24 showed prophage region homology with the M. massiliense cluster’. Please help the reader more to see this, it is impossible to guess where the strains discussed here are in this map, add some arrows.Figure 3 is likely too large in its current format for publication.

Authors’ response: *The authors improved the quality of Figures*.

p.6, l.136: ‘Interestingly”. Why? Please explain why it is interesting.

Authors’ response: *The authors clarified this point* (*P.7 line 137*).SI 1 & 2: ‘porphages’ should be prophages Figure legends: Figure 1: ‘Phylogentic’ must be fixed + see problems with Figure 4 and Figure 2 legends discussed above.

Authors’ response: *The authors corrected this point*.

p.34. Table three: what is the difference between a ‘parent species’ and a ‘donor species?’ (‘donor’ takes only 1 ‘n’). What does the column ‘Nb Duplications before parent’ refers to?

Authors’ response: *The parent species is the node which contains the two sub*-*trees*: *recipient and donor species and the number of duplication before parent refers to the number of gene duplication before the HGT event*.

### Second revision requested be the Reviewer 1: Dr. Eric Bapteste (UPMC, Institut de Biologie Paris Seine, France)

The revised version of the MS by Sassi et al. is improved. I am still uncertain whether the quantification of HGT using gene trees means much biologically. I suppose this is because I doubt that trees alone convey that kind of evidence anyway. I am more convinced by studies of synteny showing prophages with similar genes inserted at various positions of Mycobacterium genomes. I remain also unconvinced (to be honest somewhat skeptical) about the quality of the figures. I suppose this latter possible issue would be addressed by the publisher then.

p.9. l. 195. The content of Additional file 4 is useful, also not yet perfect for its purpose: both trees with and without candidate HGT are present in this file (i.e. there are around 214 trees in it, not 156 trees), making it difficult to evaluate the trees with HGT only. The legend for this file (p.27, l.614) is confusing as it seems to announce 214 trees with candidate HGT. When one looks at some of these tree files however, one finds the following associated description: ‘None horizontal gene transfert event’. (So some of these trees should be removed from Additional file 4, and the English of this final description could be improved).

Authors’ response: *In the additional file*4*we changed the report by trees as figures and it presents 75 trees representing a total of 156 transfer events. The HGT is represented by yellow squares in the figures. The legend is corrected accordingly to the reviewer*'*s comment*.

p.9. l.199-201: When discussing the sources of HGT, I find it strange that the category ‘unknown’ is not quantified, nor discussed. In the few trees I have looked at from additional file 4, ‘unknown’ was the major HGT donor…

Authors’ response: *The category* “*unknown*” *in the report files represents the taxonomy used in the project which is not that same used by NCBI. As the species name is too long we removed it and only the strain name is presented*, *meaning that the program we run does not recognize the names we gave in data. Here we present as additional file*4*the figures of the trees to avoid any confusion*.

p.10. l. 227. ‘a different topology’, sure, but a topology of what? I suppose of split networks. Indeed, the networks look different, but note that their difference is still not assessed by any formal distance computed between them, nor by any statistical test. Some might find that this aspect of the study would have deserved to be improved.

Authors’ response: *Indeed a different split network topology. This is corrected in the text Page10*, *line 227*.

All the minor comments were corrected.

### Reviewer 2: Dr. Patrick Forterre (Institut de Génétique Microbiologie, 91405 Orsay Cedex, France Institut Pasteur)

The authors have analyzed the proviruses integrated in 48 strains of the Mycobacterium abscessus complex. Interestingly, this analysis allowed detecting six new families of mycobacterioviruses, in addition to the previously described virus Araucaria. The authors observe that these viruses roughly co-evolved with their hosts since they can be divided in three clusters corresponding to the three Mycobacterium abscessus sub-complexes. However, they also noticed many incongruence between various tree topologies that are interpreted as horizontal gene transfer (HGT). It is unclear for me which of these transfer correspond to independent gain and/or loss of proviruses in different lineages of M. absessus and which ones are due to real transfer of viruses from one lineage to the other. It is also not clear why the authors concluded that viral infection contributes to the differentiation of the M. abscessus complex.

The presentation of Figures and Table could be improved. The Tables 1 and 2 could be placed in supplementary material and important information about the proviruses summarized in Figure (diagram) and/or Table (how many genomes have 0, 1, 2….7,8 integrated elements, size distribution, main features of the four classes proposed). The trees/networks are also difficult to interpret.

**Table 2:** ***M. abscessus *** **HGT cases**

**Putative HGT**	**Homology**	**Parent species**	**Recipient species**	**Donor species**	**Nb duplications before parent**
1	PHAGE_Mycoba_Peaches-gi|282598664|ref|YP_003358761.1|gp58[Mycobacterium_phage_Peaches]	[M93]	[6G-0125-R]	[M93]	5
2	PHAGE_Plankt_PaV_LD-gi|371496158|ref|YP_004957306.1|ABCtransporter[PlanktothrixphagePaV-LD]	[PSEUDO ~ −5S-0421]	[M94]	[M93 M115]	4
3	PHAGE_Mycoba_LeBron-gi|304360967|ref|YP_003857149.1|gp18[Mycobacterium_phage_LeBron]	[M115]	[M172]	[M115]	2
4	PHAGE_Mycoba_Giles-gi|160700672|ref|YP_001552352.1|gp23[Mycobacterium_phage_Giles]	[M94]	[6G-0728-R]	[M94]	5
5	PHAGE_Mycoba_Che9c-gi|29566118|ref|NP_817687.1|gp10[Mycobacterium_phage_Che9c]	[M18]	[3A-0122_S]	[M18]	2
6	PHAGE_Mycoba_Pukovnik-gi|192824238|ref|YP_001994879.1|gp62[Mycobacterium_phage_Pukovnik]	[M154]	[M115]	[M154]	5
7	PHAGE_Tricho_2c-gi|116326757|ref|YP_803294.1|hypotheticalproteinTNAV2c_gp071[Trichoplusia_ni_ascovirus_2c]	[5S-0921]	[4S-0726]	[5S-0921]	4
8	PHAGE_Salmon_PVP_SE1-gi|363539742|ref|YP_004894027.1|hypotheticalprotein[SalmonellaphagePVP-SE1]	[M. massiliense T]	[M24]	[M. massiliense T]	5
9	PHAGE_Rhodoc_REQ3-gi|372449972|ref|YP_005087193.1|phageintegrase[RhodococcusphageREQ3]	[M172]	[3A_0930_S]	[M172]	3
10	PHAGE_Salmon_PVP_SE1-gi|363539618|ref|YP_004893903.1|phosphoribosylpyrophosphatesynthetase[SalmonellaphagePVP-SE1]	[3A-0122_S1]	[M. massiliense T]	[3A-0122_S1]	5
11	PHAGE_Mycoba_Myrna-gi|203454746|ref|YP_002225062.1|gp183[Mycobacterium_phage_Myrna]	[M172]	[M94]	[M172]	5
12	PHAGE_Mycoba_Omega-gi|29566822|ref|NP_818386.1|gp85[Mycobacterium_phage_Omega]	[M115]	[M24]	[M115]	5
13	PHAGE_Mycoba_Pacc40-gi|206600097|ref|YP_002241602.1|gp18[Mycobacterium_phage_Pacc40]	[M24]	[M94]	[M24]	6
14	PHAGE_Mycoba_Pacc40-gi|206600097|ref|YP_002241602.1|gp18[Mycobacterium_phage_Pacc40]	[PSEUDO ~ − M159]	[M24]	[M172 3A-0122_S6 3A-0122_S2 3A-0122_S4 47 J26]	1
15	PHAGE_Acanth_mimivirus-gi|311977570|ref|YP_003986690.1|DNAtopoisomerase1b[Acanthamoebapolyphagamimivirus]	[3A-0122_S7]	[3A-0122_S5]	[3A-0122_S7]	2
16	PHAGE_Rhodoc_RER2-gi|372449922|ref|YP_005087145.1|hypotheticalprotein[RhodococcusphageRER2]	[M24]	[3A-0731]	[M24]	3
17	PHAGE_Aeromo_31-gi|66391812|ref|YP_238737.1|hypotheticalproteinPHG31p8[Aeromonas_phage_31]	[4S-0726]	[4S-0303]	[4S-0726]	6
18	PHAGE_Lactoc_P087-gi|229605000|ref|YP_002875699.1|putativecysteinesynthase[Lactococcus_phage_P087]	[47 J26]	[5S-0708]	[47 J26]	6
19	PHAGE_Mycoba_Myrna-gi|203454746|ref|YP_002225062.1|gp183[Mycobacterium_phage_Myrna]	[M172]	[M94]	[M172]	5
20	PHAGE_Plankt_PaV_LD-gi|371496158|ref|YP_004957306.1|ABCtransporter[PlanktothrixphagePaV-LD]	[3A-0122_S5]	[M94]	[3A-0122_S5]	2
21	PHAGE_Acanth_mimivirus-gi|311977513|ref|YP_003986633.1|putativedTDP-D-glucose4,6-dehydratase[Acanthamoebapolyphagamimivirus]	[3A-0122_S4]	[4S-0726-RA]	[3A-0122_S4]	4
22	PHAGE_Bacill_36-gi|156564011|ref|YP_001429750.1|PcrAhelicase[Bacillus_phage_0305phi8_36]	[3A-0122_S7]	[3A-0119-R]	[3A-0122_S7]	3
23	PHAGE_Mycoba_Che9c-gi|29566174|ref|NP_817745.1|gp68[Mycobacterium_phage_Che9c]	[M18]	[M94]	[M18]	3
24	PHAGE_Mycoba_Che8-gi|29565783|ref|NP_817355.1|gp17[Mycobacterium_phage_Che8]	[M24]	[M172 3A-0122_S4 3A-0122_S2 47 J26]	[M24]	2
25	PHAGE_Tricho_2c-gi|116326757|ref|YP_803294.1|hypotheticalproteinTNAV2c_gp071[Trichoplusia_ni_ascovirus_2c]	[M115]	[4S-0116_S]	[M115]	3
26	PHAGE_Microm_MpV1-gi|313768434|ref|YP_004062114.1|hypotheticalprotein[Micromonassp.RCC1109virusMpV1]	[M115]	[M94]	[M115]	3
27	PHAGE_Mycoba_Pipefish-gi|109521870|ref|YP_655307.1|gp30[Mycobacterium_phage_Pipefish]	[M18]	[M172]	[M18]	1
28	PHAGE_Plankt_PaV_LD-gi|371496158|ref|YP_004957306.1|ABCtransporter[PlanktothrixphagePaV-LD]	[M. bolletii T]	[4S-0726-RA]	[M. bolletii T]	7
29	PHAGE_Lactoc_P087-gi|229605000|ref|YP_002875699.1|putativecysteinesynthase [Lactococcus_phage_P087]	[M24]	[M. bolletii T]	[M24]	7
30	PHAGE_Mycoba_Omega-gi|29566768|ref|NP_818332.1|gp31[Mycobacterium_phage_Omega]	[M94]	[6G-1108]	[M94]	3
31	PHAGE_Burkho_phi1026b-gi|38707948|ref|NP_945089.1|gp58[Burkholderia_phage_phi1026b]	[PSEUDO ~ −M154]	[M. bolletii T]	[M. massiliense T M154 M172 M159]	6
32	PHAGE_Mycoba_Cjw1-gi|29565933|ref|NP_817504.1|gp55[Mycobacterium_phage_Cjw1]	[PSEUDO ~ −M159]	[M. bolletii T]	[M172 3A-0122_S0 1S-152-0930 3A-0122_S1]	2
33	PHAGE_Brocho_BL3-gi|327409421|ref|YP_004301563.1|gp29[BrochothrixphageBL3]	[M115]	[M24]	[M115]	5

There are several minor points Lane 123; define prophage region homology.

Authors’ response: *This methodology section has been re*-*written* (*Lines 112*–*123*).

Lane 128: 242,067 proteins (which proteins?) in general be more precise

Authors’ response: *The authors clarified this point* (*P.7 line 139*).

Lane 140 and elsewhere, gram-positive bacteria is no more a valid taxonomic grouping, better to indicate Firmicutes

Authors’ response: *The authors corrected this point* (*P.7 line 151*).

Lane 168: Auracaria should be Araucaria

Authors’ response: *The authors corrected this point* (*P.8 line 182*).

Lane 193: gram-negative and gram positive bacteria! i.e. all bacteria except mycobacteria?? So environmental bacteria is sufficient.

Authors’ response: *The authors corrected this point* (*P.9 line 206*).

### Reviewer 3: Dr. Eugene Koonin (National Center for Biotechnology Information, National Library of Medicine, National Institutes of Health, Bethesda, MD, USA)

The importance of bacteriophage contribution to the evolution of bacterial genomes is increasingly recognized. Here Sassi and coworkers conclude that bacteriophages drive the evolution of the Mycobacterium abscessus complex. I find this appealing and credible idea but fail to see how the data presented in the manuscript, even assuming that the identification of prophages is accurate (no specific evidence of that is provided), support such a strong conclusion. I can agree that the authors demonstrate differences in the prophage content between the bacteria in the complex. Then, I suppose, the argument would be that the trees of the bacteria and phages are different, suggesting that there has been some exchange of prophages and individual genes. The robustness of the trees is a concern because the trees for phages can be notoriously difficult. But, even assuming they are correct, this argument seems to fall far short of the ambitious claim of the paper. It could be very helpful if the authors made an effort to carefully present their logic.

Authors’ response: *The authors revised the manuscript in light of the reviewer comments to further discuss the impact of mycobacteriophages on the on*-*going diversification of this group of mycobacteria*. (*Lines 245*–*250*).

## Competing interests

The authors declare that they have no competing interests.

## Authors’ contributions

MS, PG and OC performed the analyses. MS and MD designed the study. MS, PP and MD interpreted data and wrote the draft. All authors read and approved the final manuscript.

## Supplementary Material

Additional file 1 ***Mycobacterium abscessus *** **genome properties and their prophage regions.** Click here for file

Additional file 2 ***Mycobacterium abscessus *** **CRISPRs and the correlation with number of prophage regions.** Click here for file

Additional file 3 **The reconstructed trees for HGT events.** Each tree contains one to six HGT events. The yellow squares represent the HGT event. Click here for file

Additional file 4 **Environmental bacteria hosting homologous *M. abscessus *** **phage proteins and evidence for bacteria-amoeba interaction.** Click here for file
