# Supplementary figures and images for: Mycobacteriophage-drived diversification of Mycobacterium abscessus
Source: Biol Direct. 2014 Sep 15;9:19. doi: 10.1186/1745-6150-9-19 (PMC4172396; doi:10.1186/1745-6150-9-19)

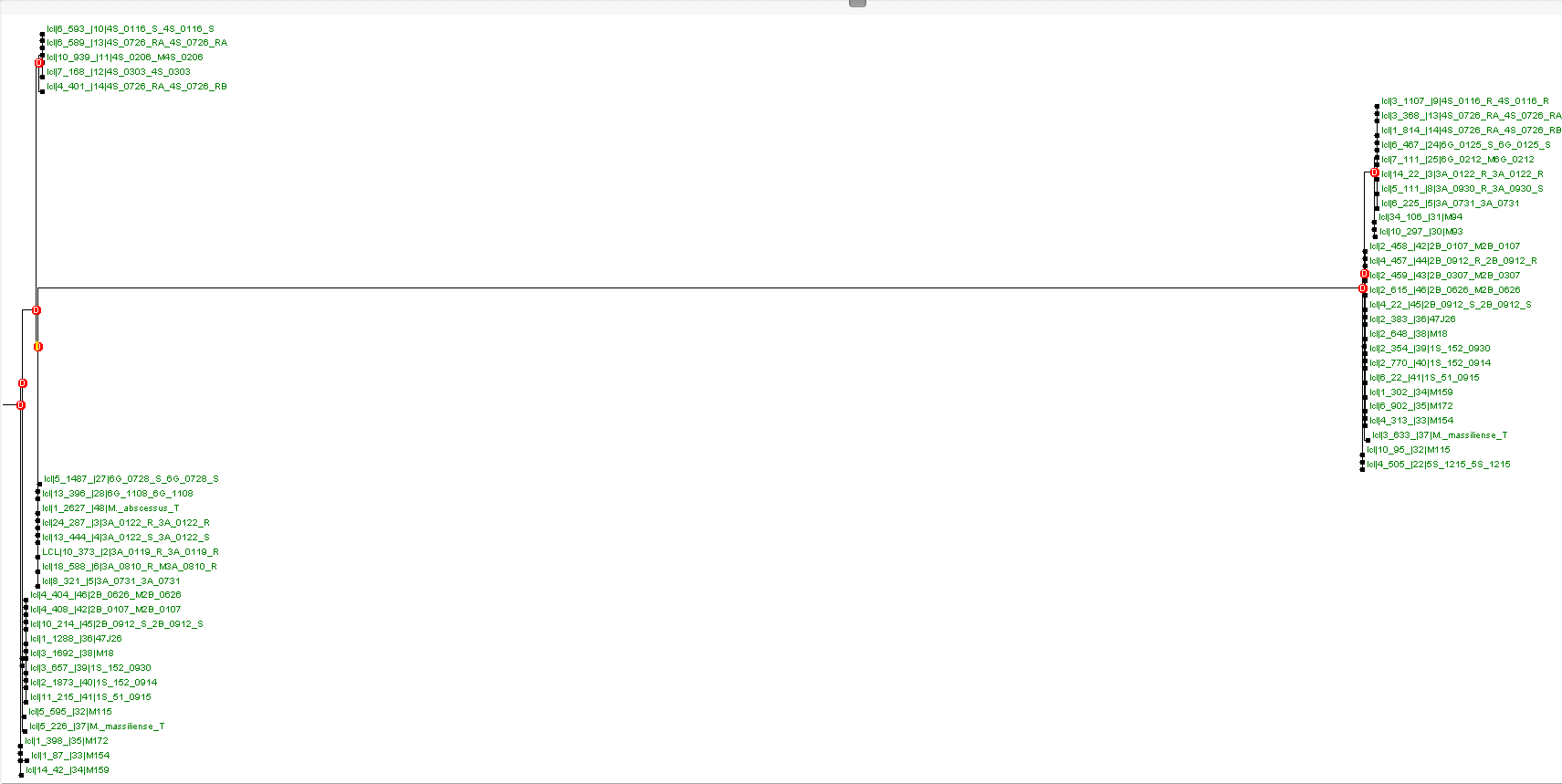

Supplement: Additional file 3 — The reconstructed trees for HGT events. Each tree contains one to six HGT events. The yellow squares represent the HGT event. [file 1745-6150-9-19-S3.zip › Tree_1.png]

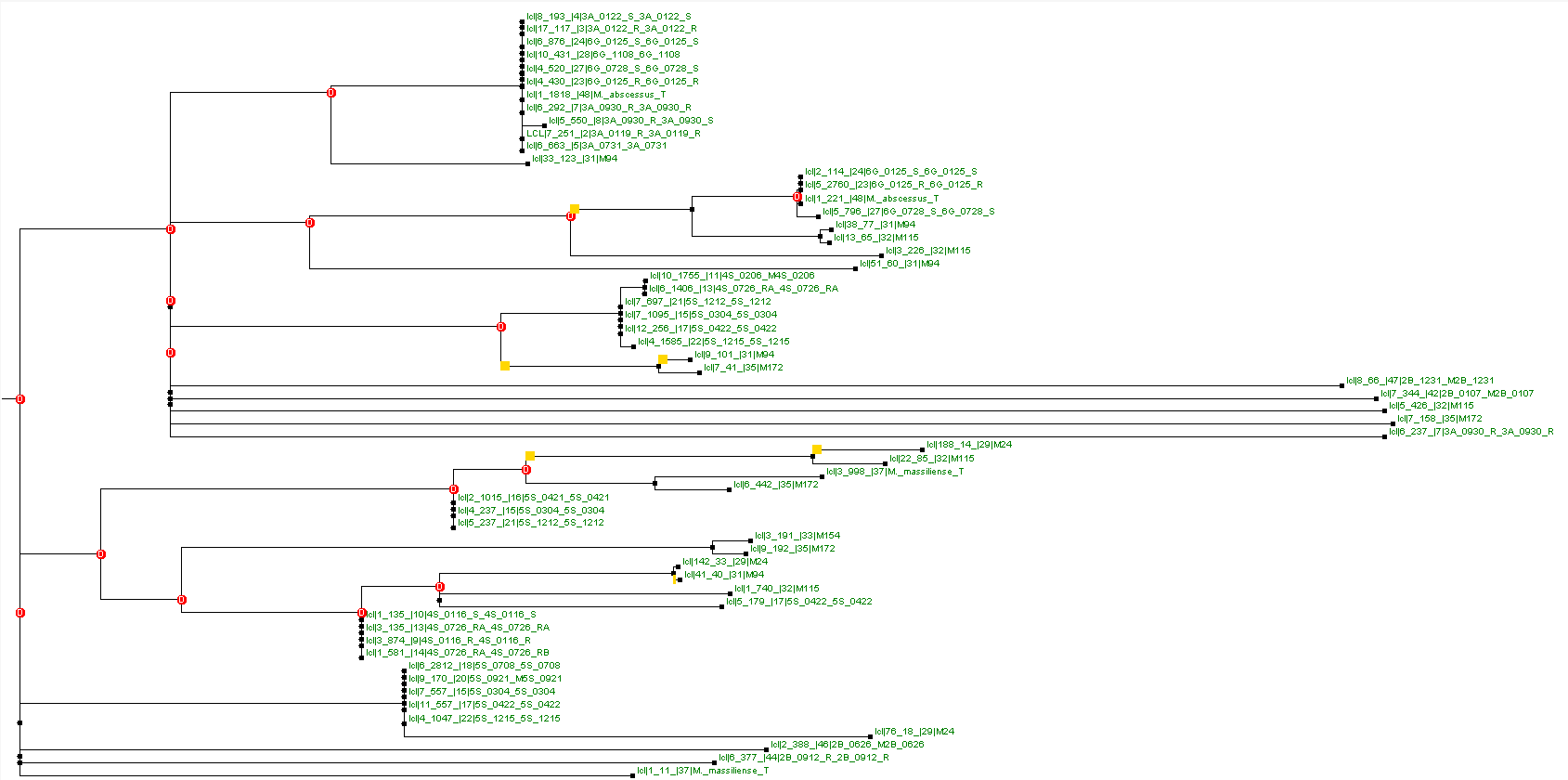

Supplement: Additional file 3 — The reconstructed trees for HGT events. Each tree contains one to six HGT events. The yellow squares represent the HGT event. [file 1745-6150-9-19-S3.zip › Tree_10.png]

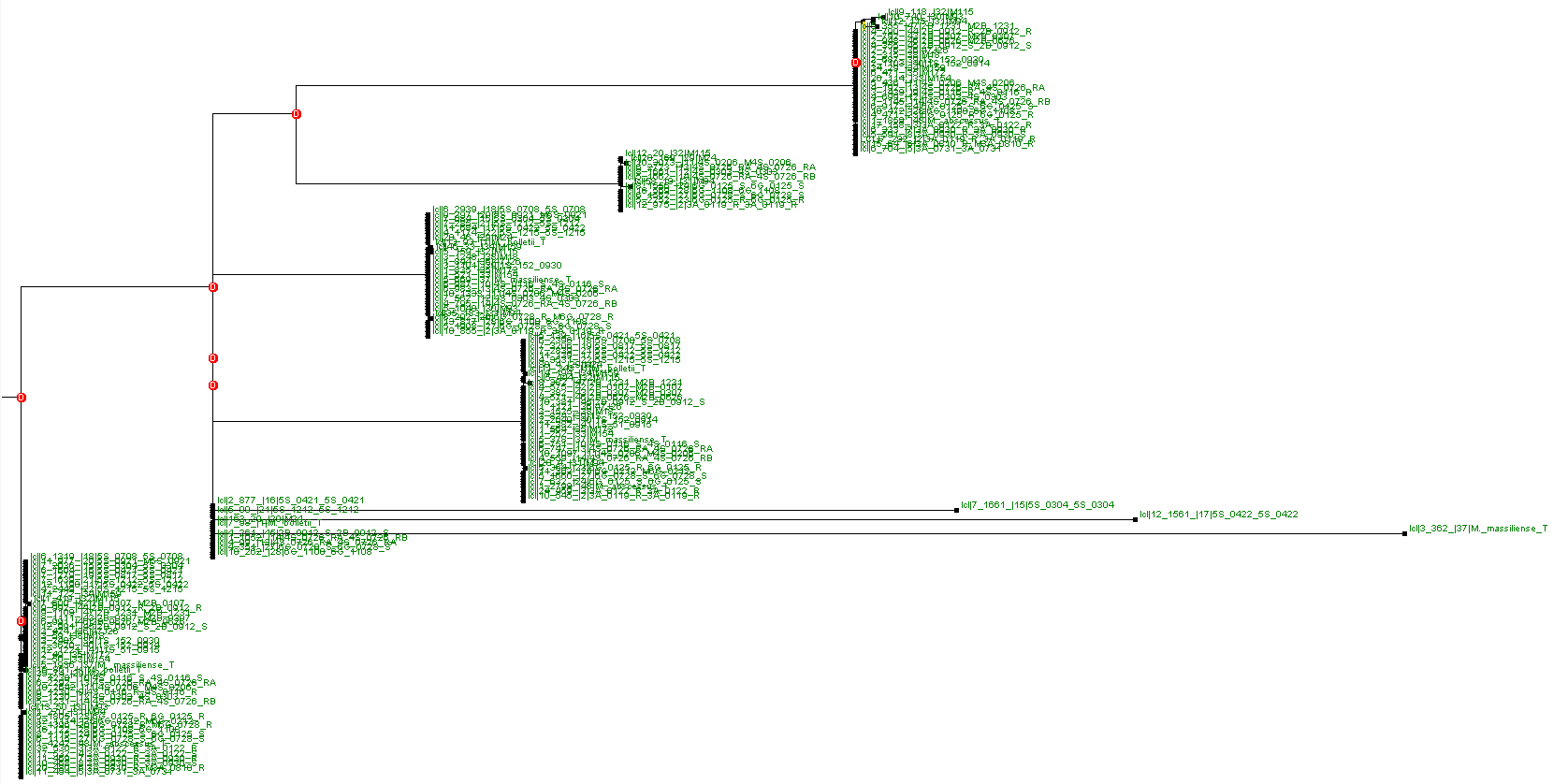

Supplement: Additional file 3 — The reconstructed trees for HGT events. Each tree contains one to six HGT events. The yellow squares represent the HGT event. [file 1745-6150-9-19-S3.zip › Tree_11.png]

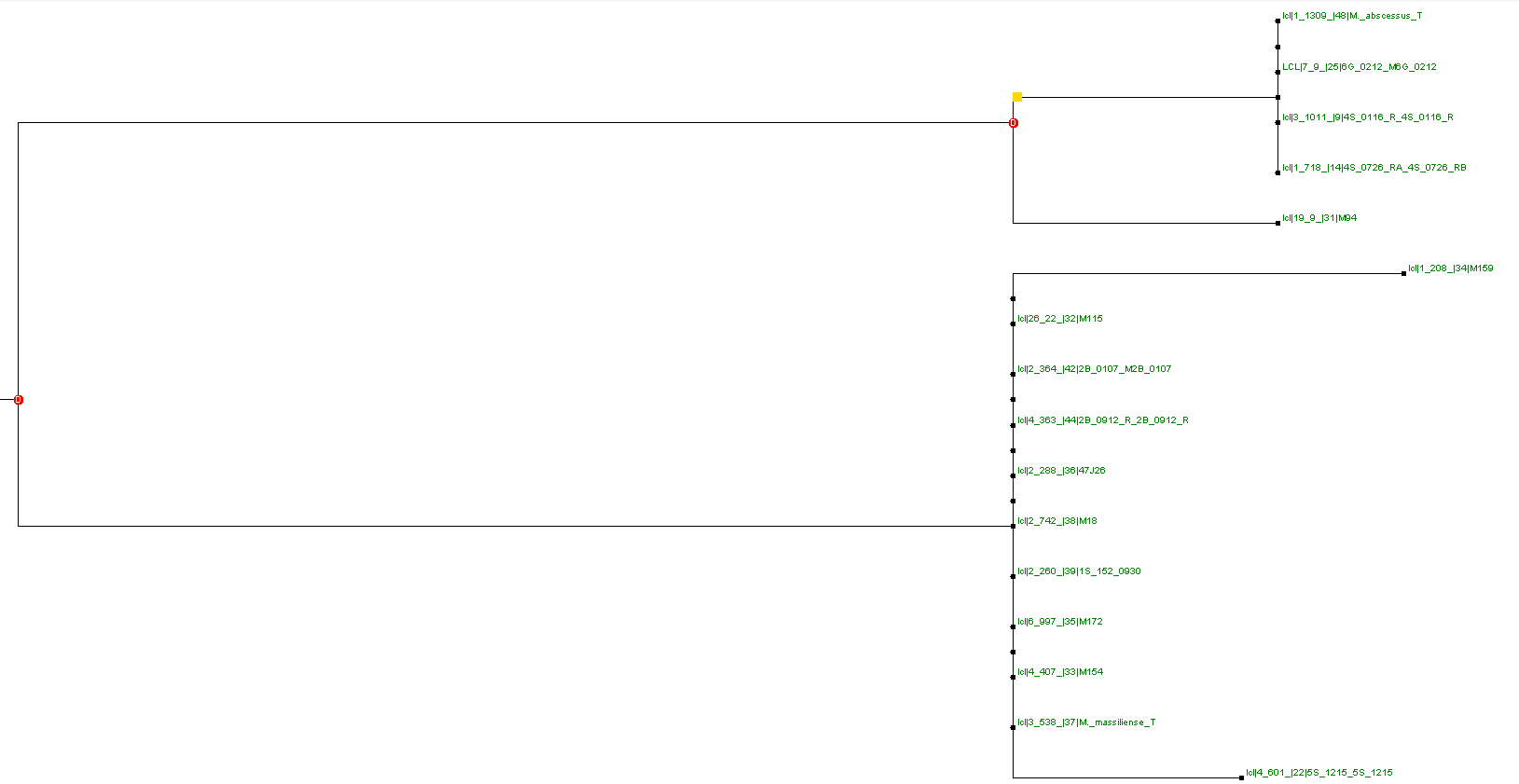

Supplement: Additional file 3 — The reconstructed trees for HGT events. Each tree contains one to six HGT events. The yellow squares represent the HGT event. [file 1745-6150-9-19-S3.zip › Tree_12.png]

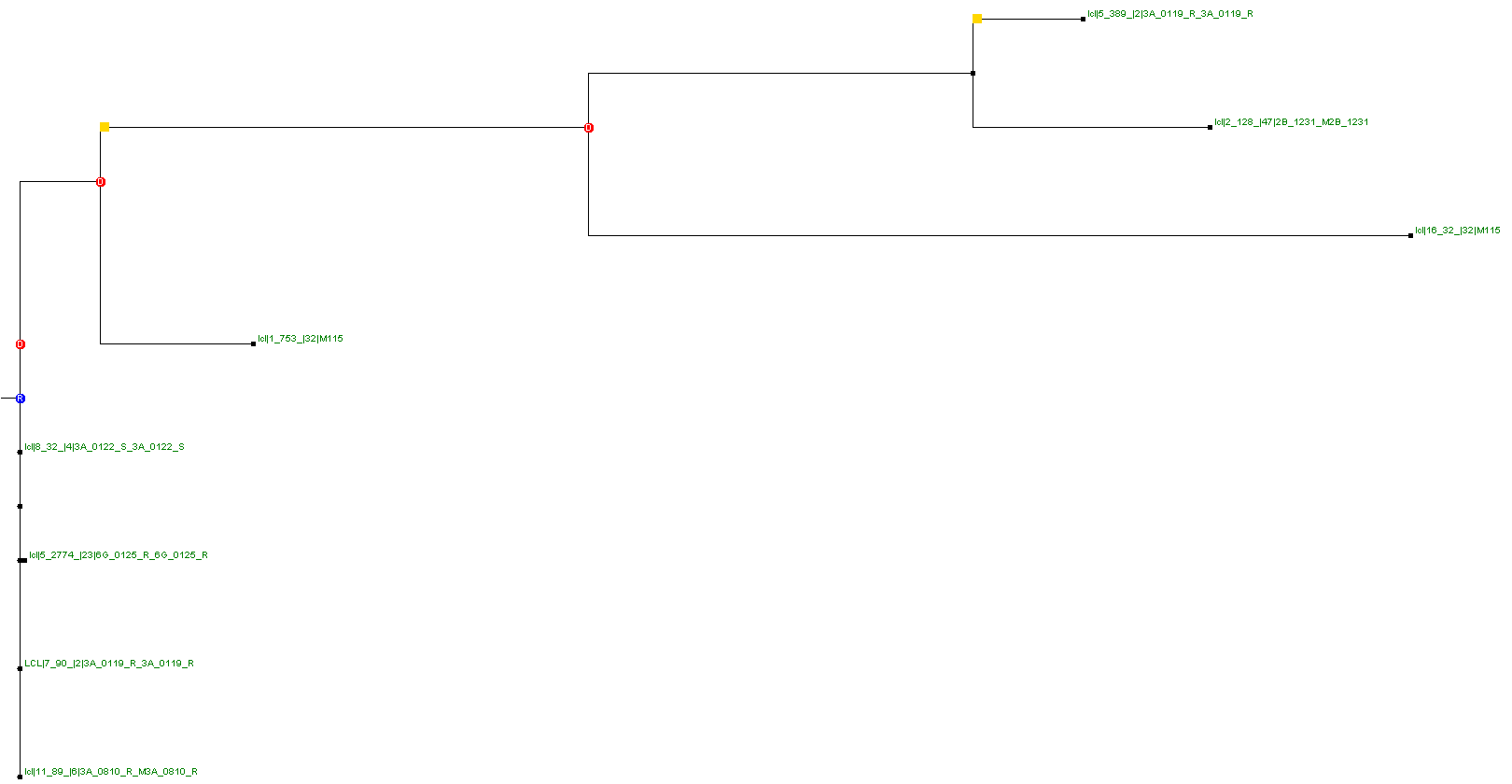

Supplement: Additional file 3 — The reconstructed trees for HGT events. Each tree contains one to six HGT events. The yellow squares represent the HGT event. [file 1745-6150-9-19-S3.zip › Tree_13.png]

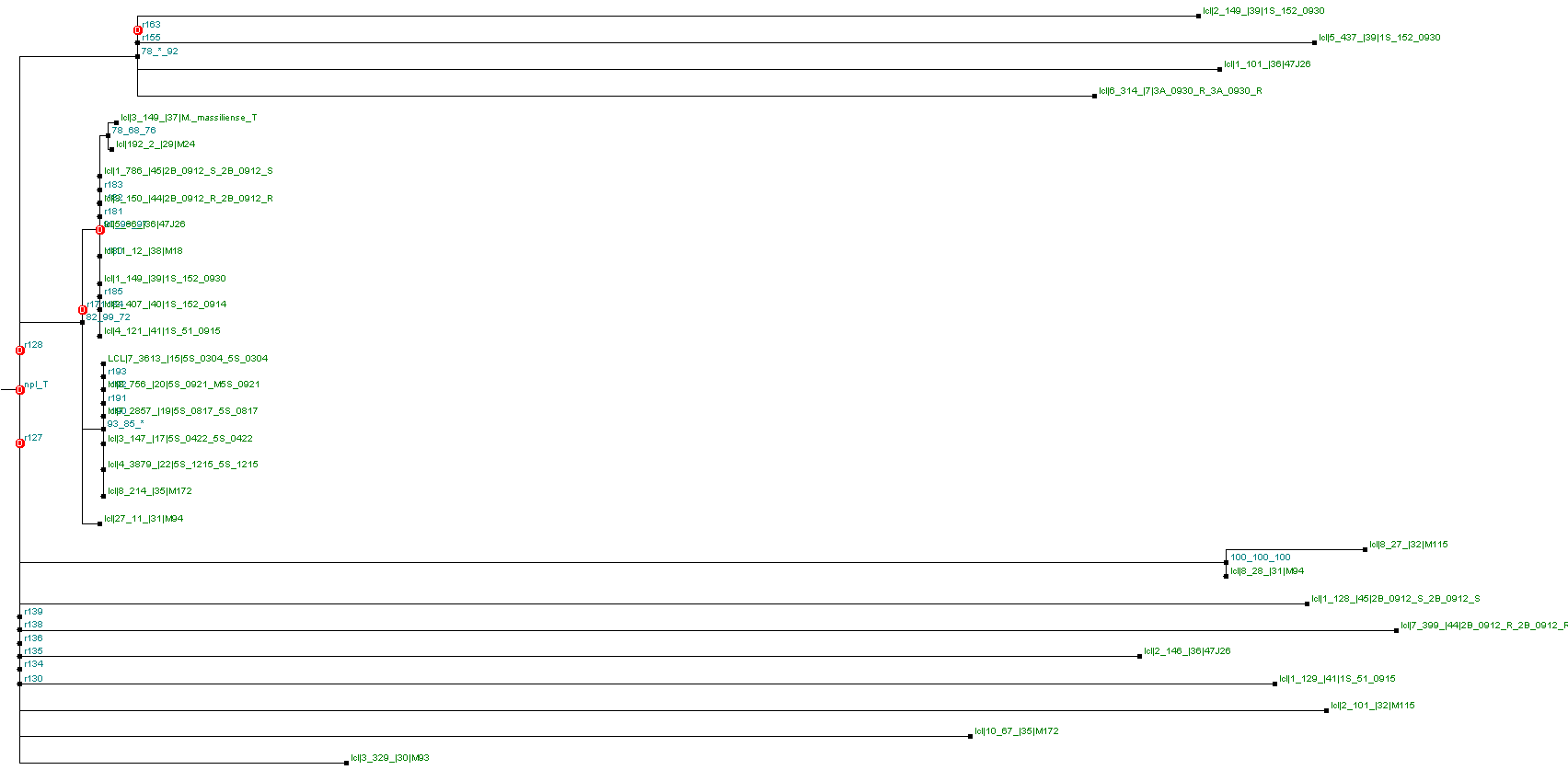

Supplement: Additional file 3 — The reconstructed trees for HGT events. Each tree contains one to six HGT events. The yellow squares represent the HGT event. [file 1745-6150-9-19-S3.zip › Tree_14.png]

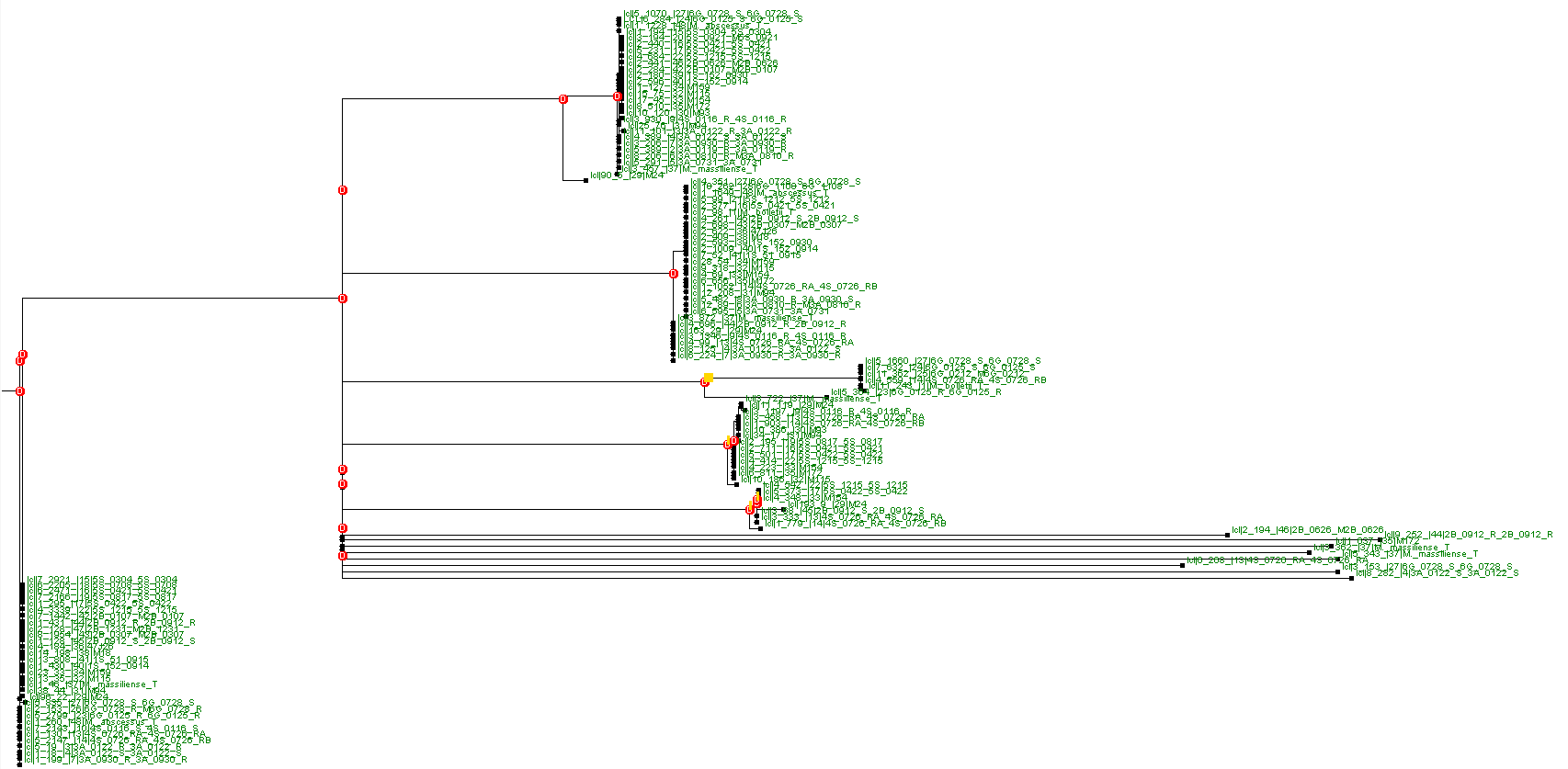

Supplement: Additional file 3 — The reconstructed trees for HGT events. Each tree contains one to six HGT events. The yellow squares represent the HGT event. [file 1745-6150-9-19-S3.zip › Tree_15.png]

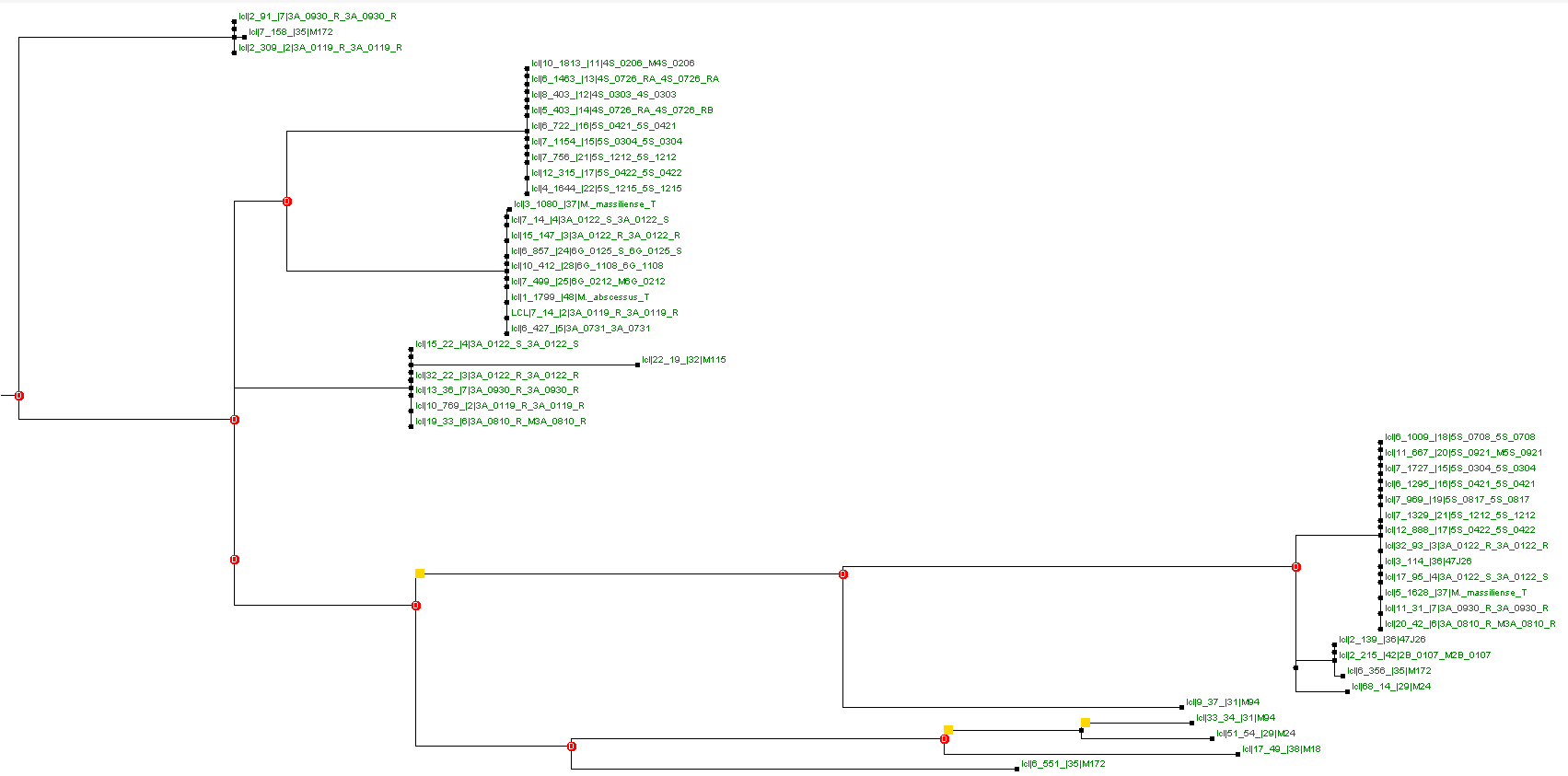

Supplement: Additional file 3 — The reconstructed trees for HGT events. Each tree contains one to six HGT events. The yellow squares represent the HGT event. [file 1745-6150-9-19-S3.zip › Tree_16.png]

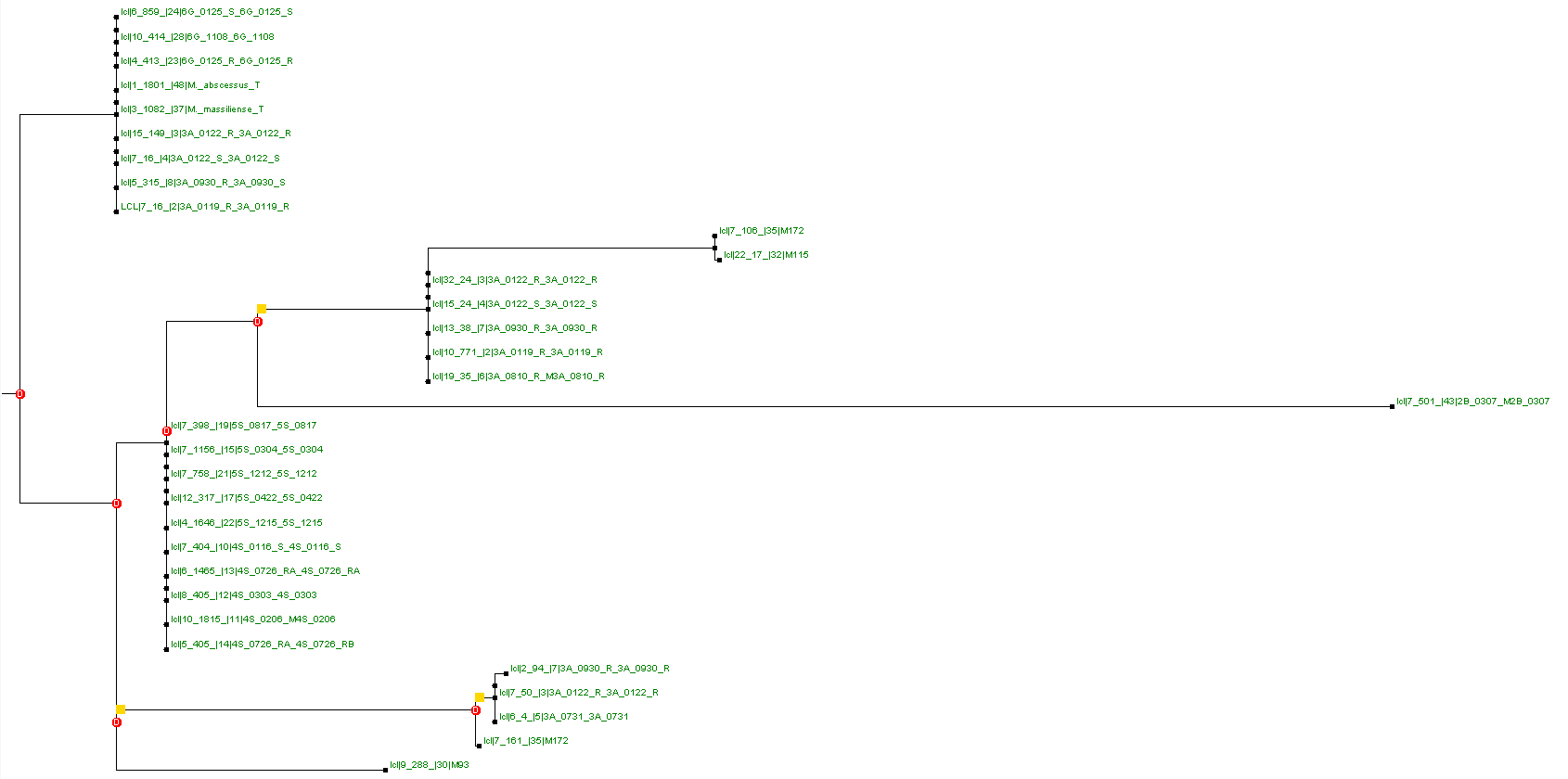

Supplement: Additional file 3 — The reconstructed trees for HGT events. Each tree contains one to six HGT events. The yellow squares represent the HGT event. [file 1745-6150-9-19-S3.zip › Tree_17.png]

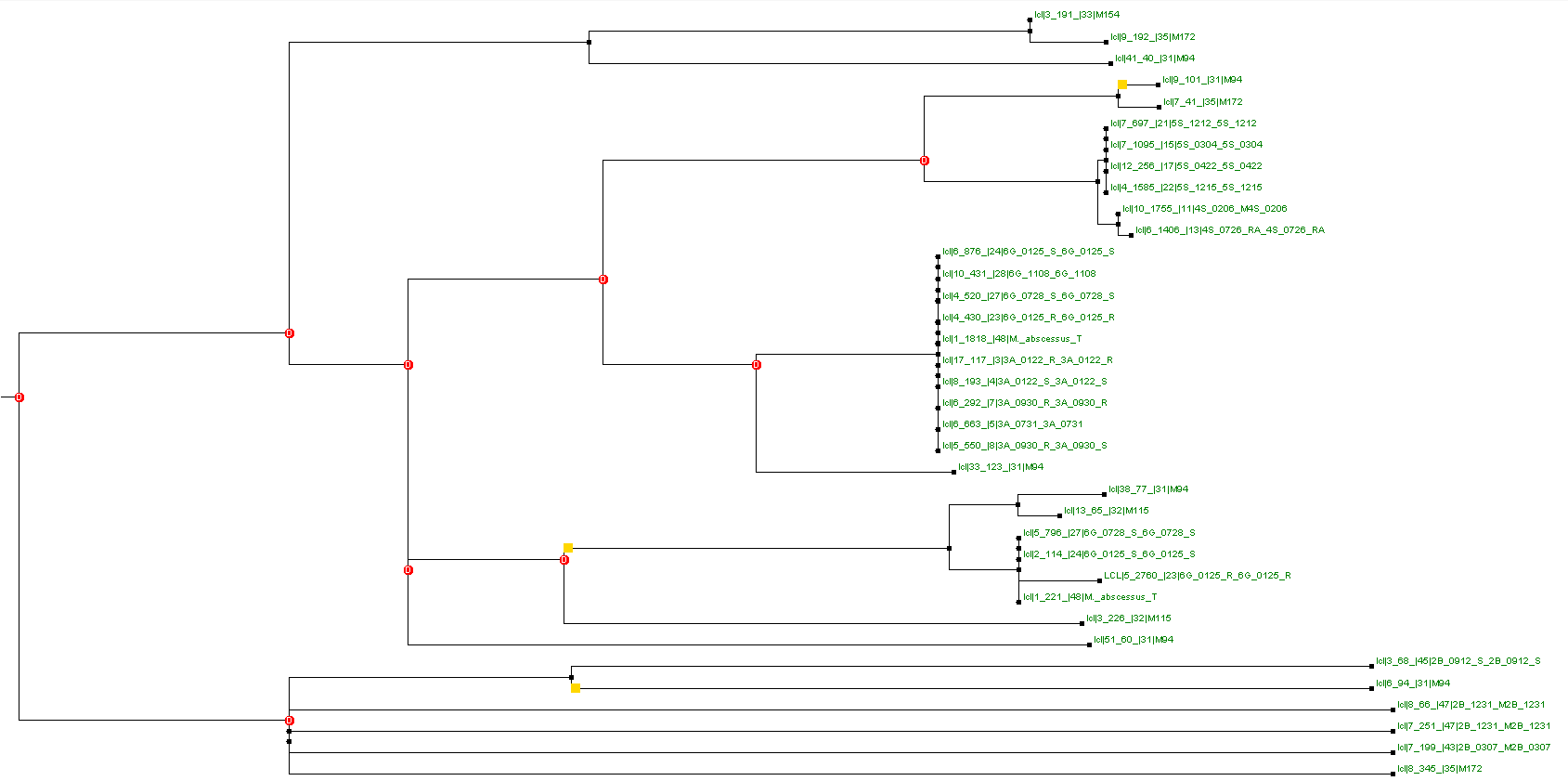

Supplement: Additional file 3 — The reconstructed trees for HGT events. Each tree contains one to six HGT events. The yellow squares represent the HGT event. [file 1745-6150-9-19-S3.zip › Tree_18.png]

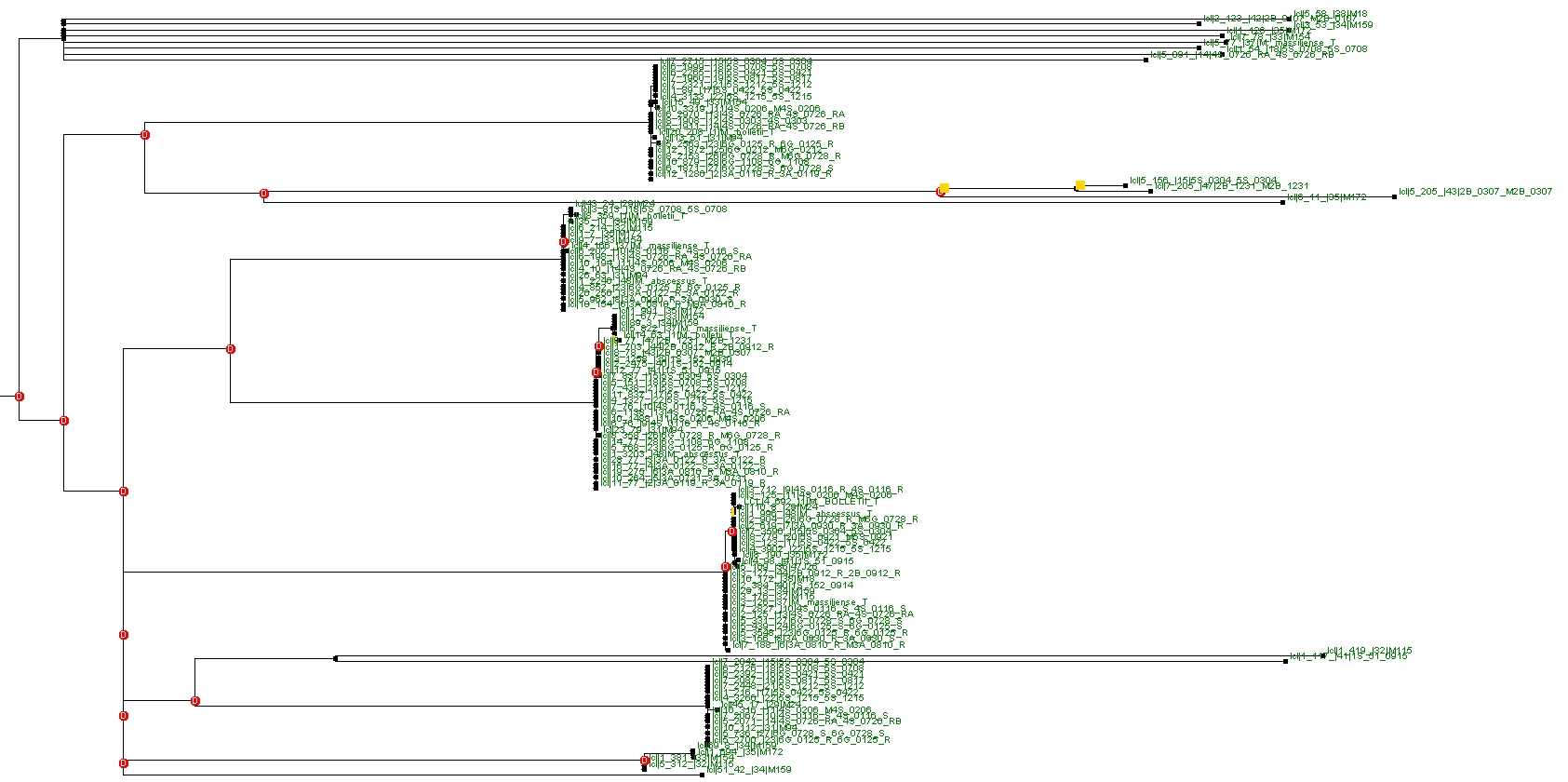

Supplement: Additional file 3 — The reconstructed trees for HGT events. Each tree contains one to six HGT events. The yellow squares represent the HGT event. [file 1745-6150-9-19-S3.zip › Tree_19.png]

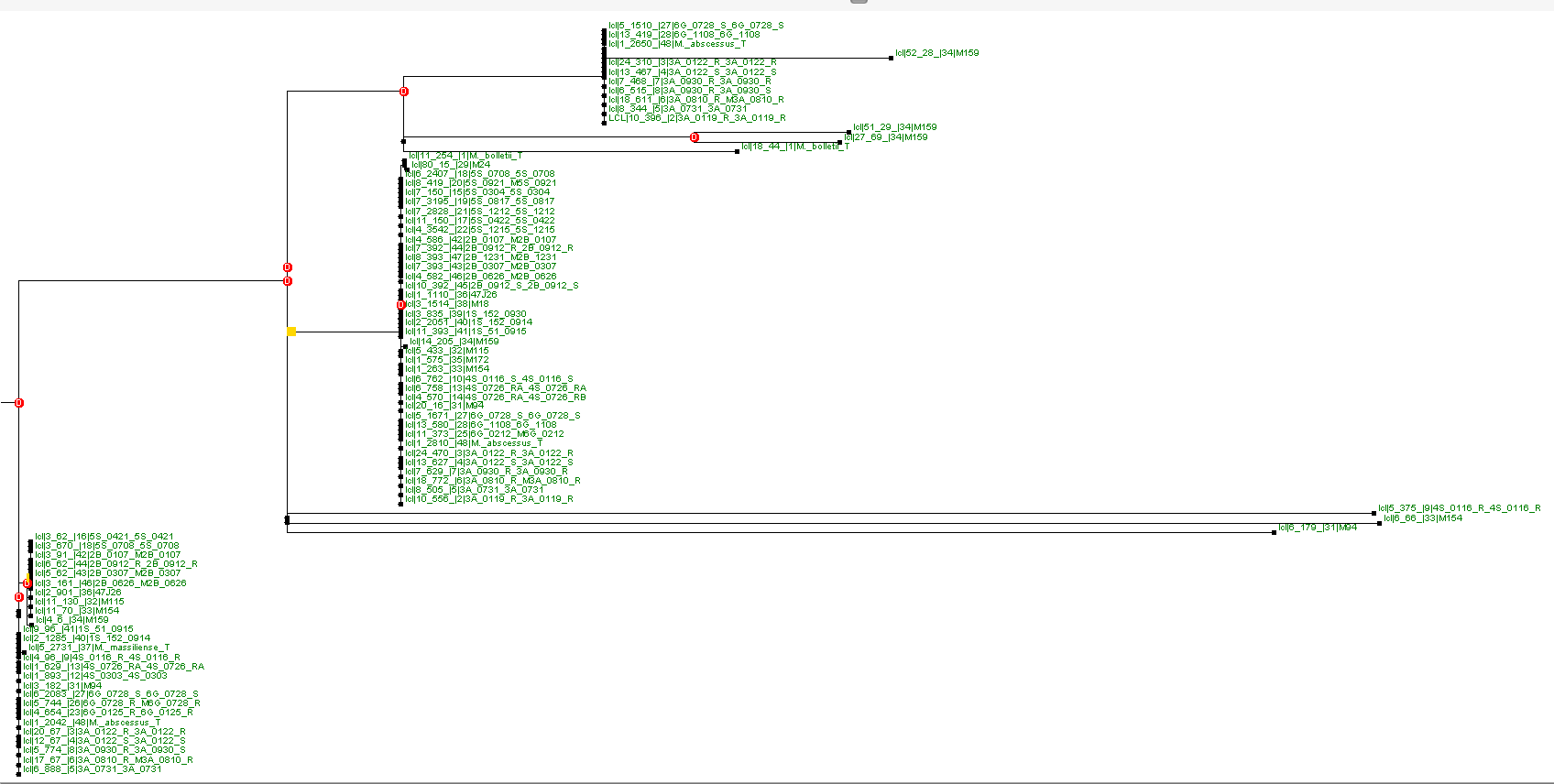

Supplement: Additional file 3 — The reconstructed trees for HGT events. Each tree contains one to six HGT events. The yellow squares represent the HGT event. [file 1745-6150-9-19-S3.zip › Tree_2.png]

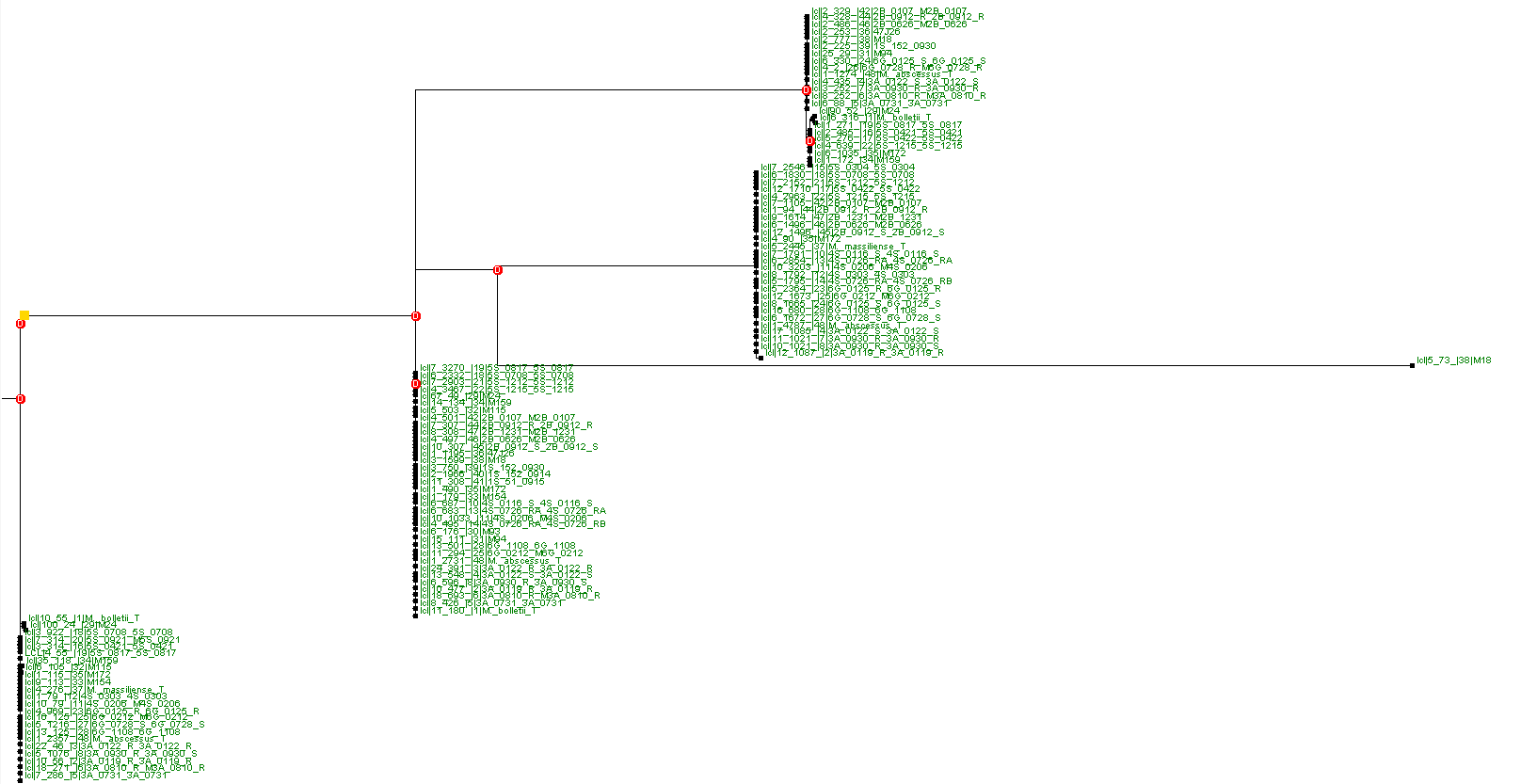

Supplement: Additional file 3 — The reconstructed trees for HGT events. Each tree contains one to six HGT events. The yellow squares represent the HGT event. [file 1745-6150-9-19-S3.zip › Tree_20.png]

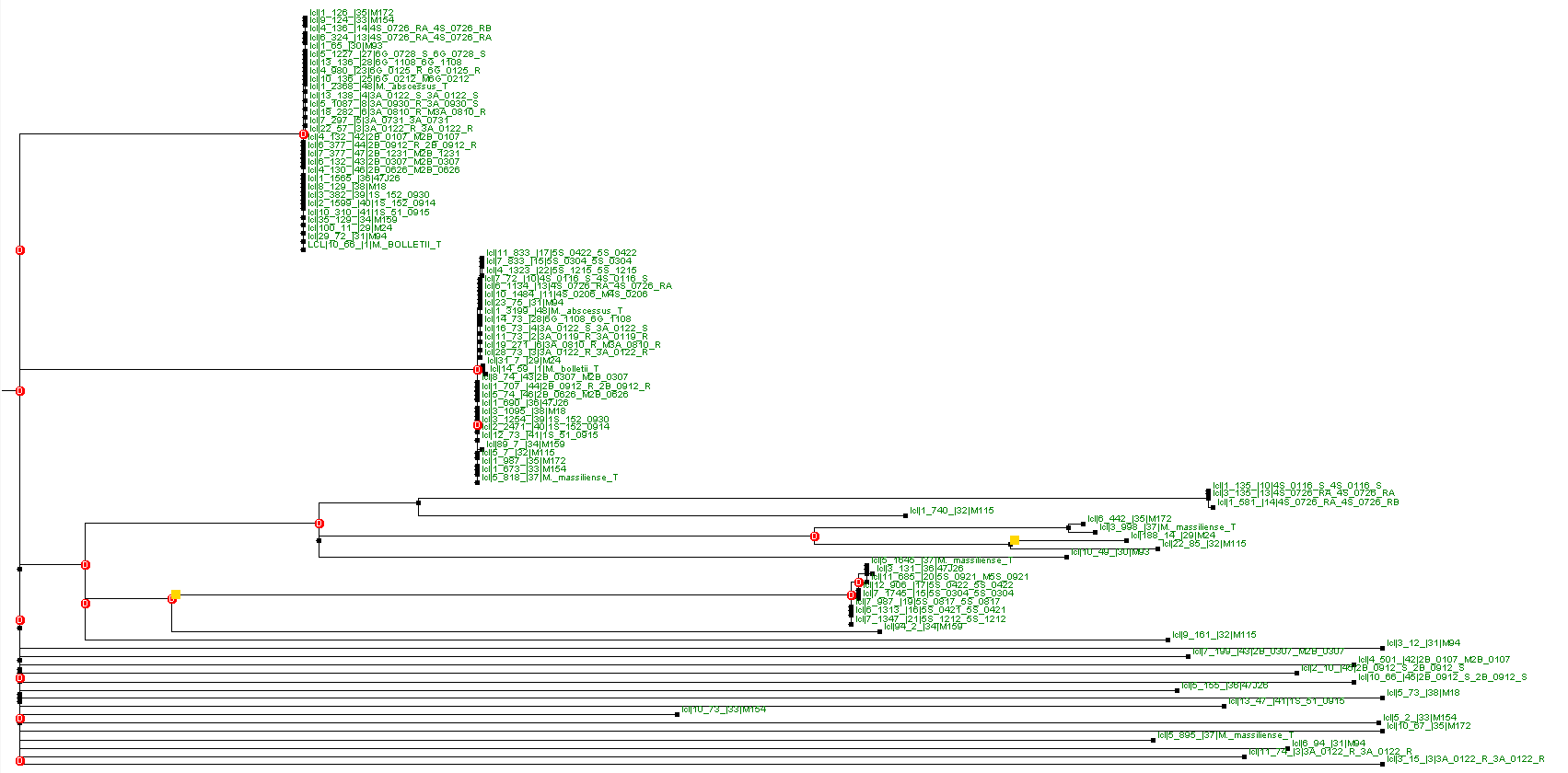

Supplement: Additional file 3 — The reconstructed trees for HGT events. Each tree contains one to six HGT events. The yellow squares represent the HGT event. [file 1745-6150-9-19-S3.zip › Tree_21.png]

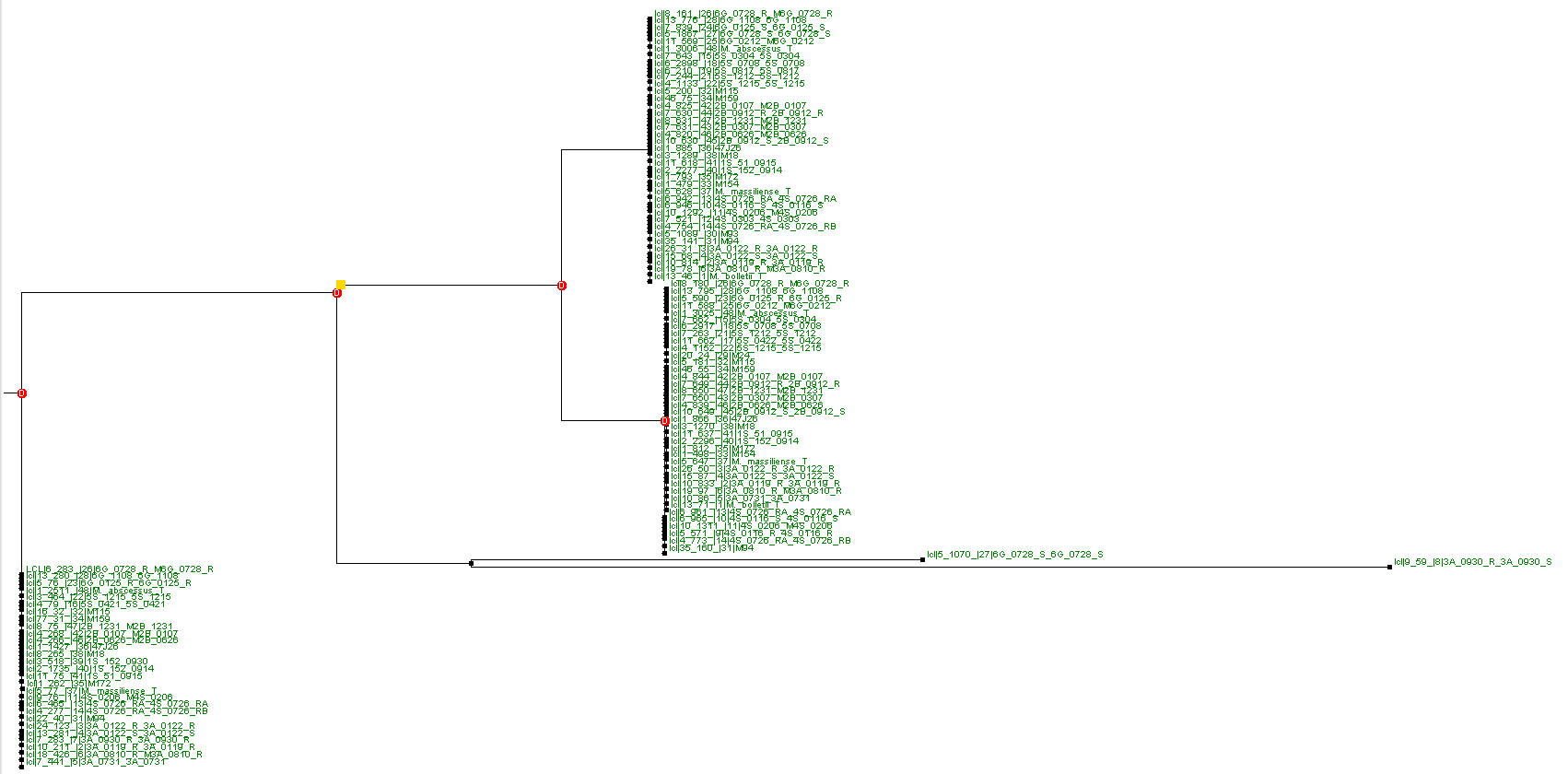

Supplement: Additional file 3 — The reconstructed trees for HGT events. Each tree contains one to six HGT events. The yellow squares represent the HGT event. [file 1745-6150-9-19-S3.zip › Tree_22.png]

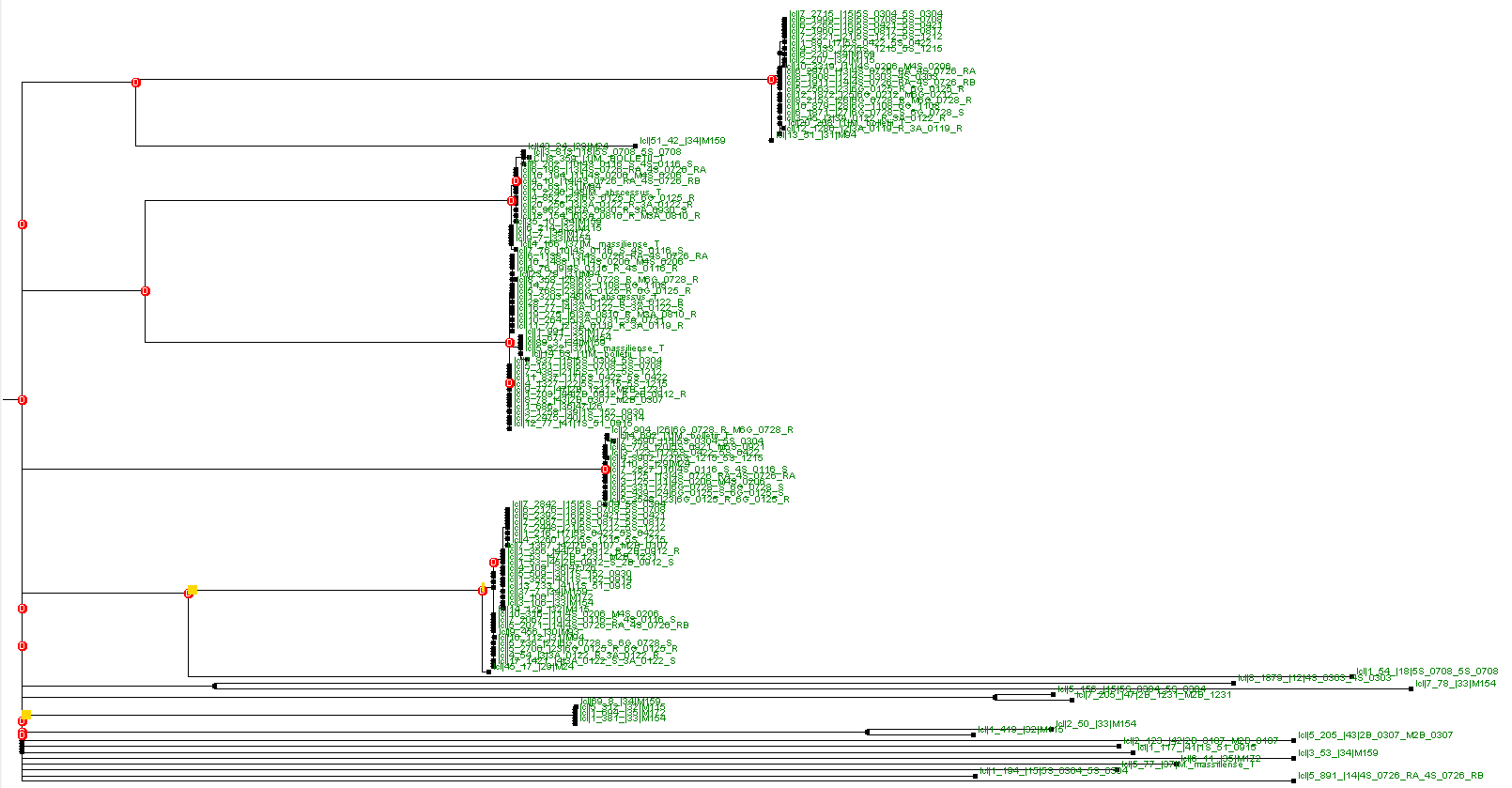

Supplement: Additional file 3 — The reconstructed trees for HGT events. Each tree contains one to six HGT events. The yellow squares represent the HGT event. [file 1745-6150-9-19-S3.zip › Tree_23.png]

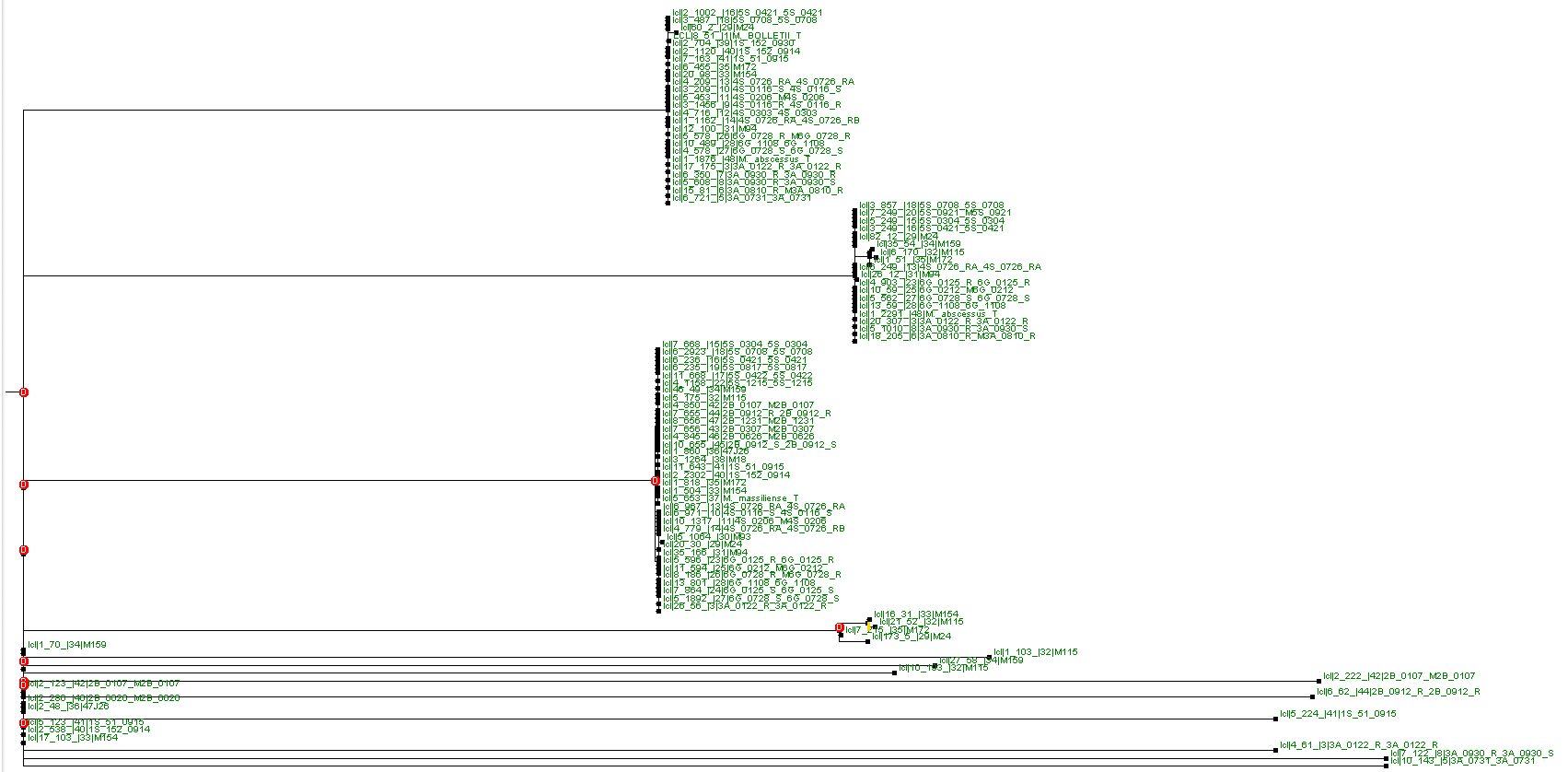

Supplement: Additional file 3 — The reconstructed trees for HGT events. Each tree contains one to six HGT events. The yellow squares represent the HGT event. [file 1745-6150-9-19-S3.zip › Tree_24.png]

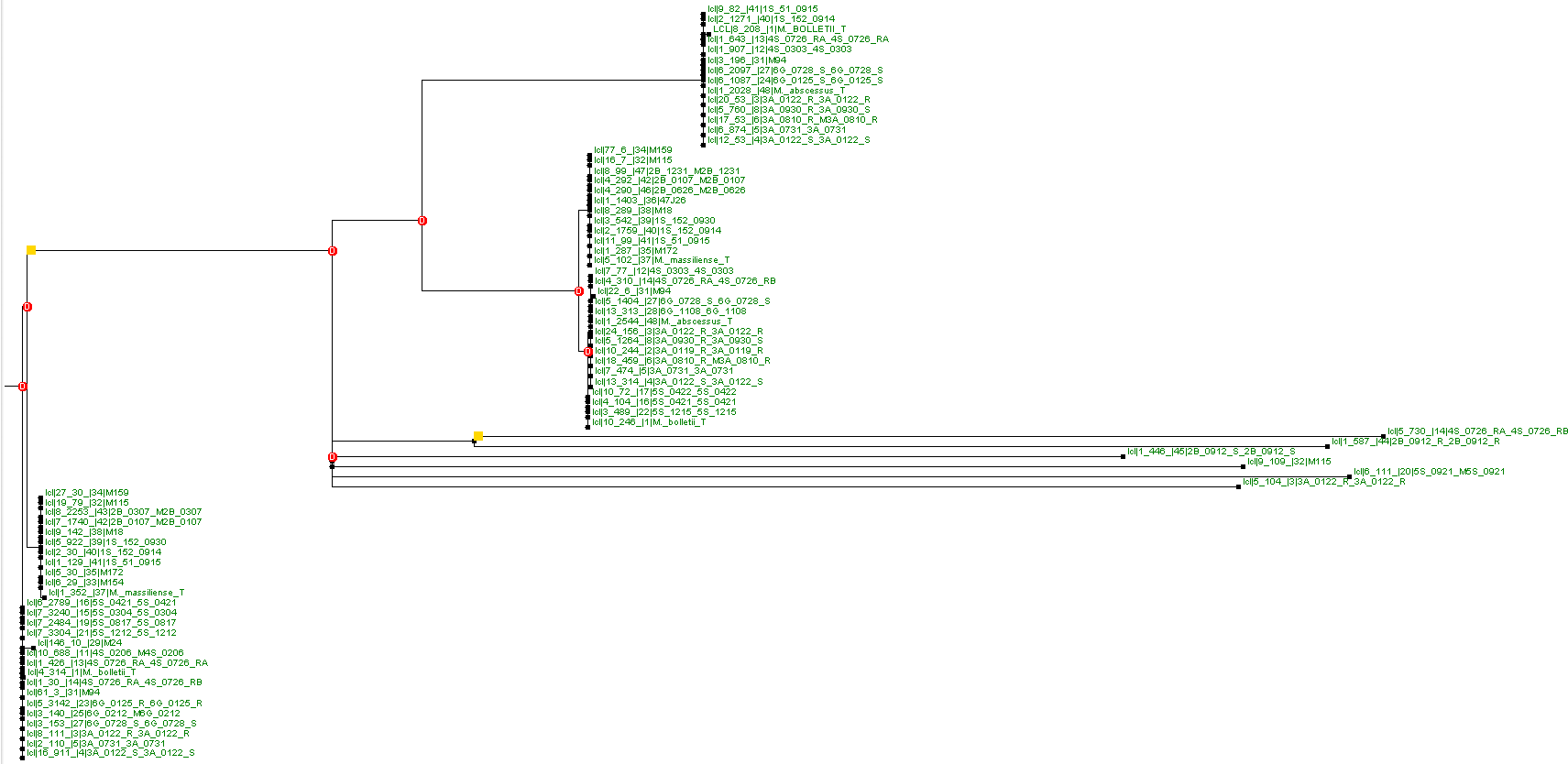

Supplement: Additional file 3 — The reconstructed trees for HGT events. Each tree contains one to six HGT events. The yellow squares represent the HGT event. [file 1745-6150-9-19-S3.zip › Tree_25.png]

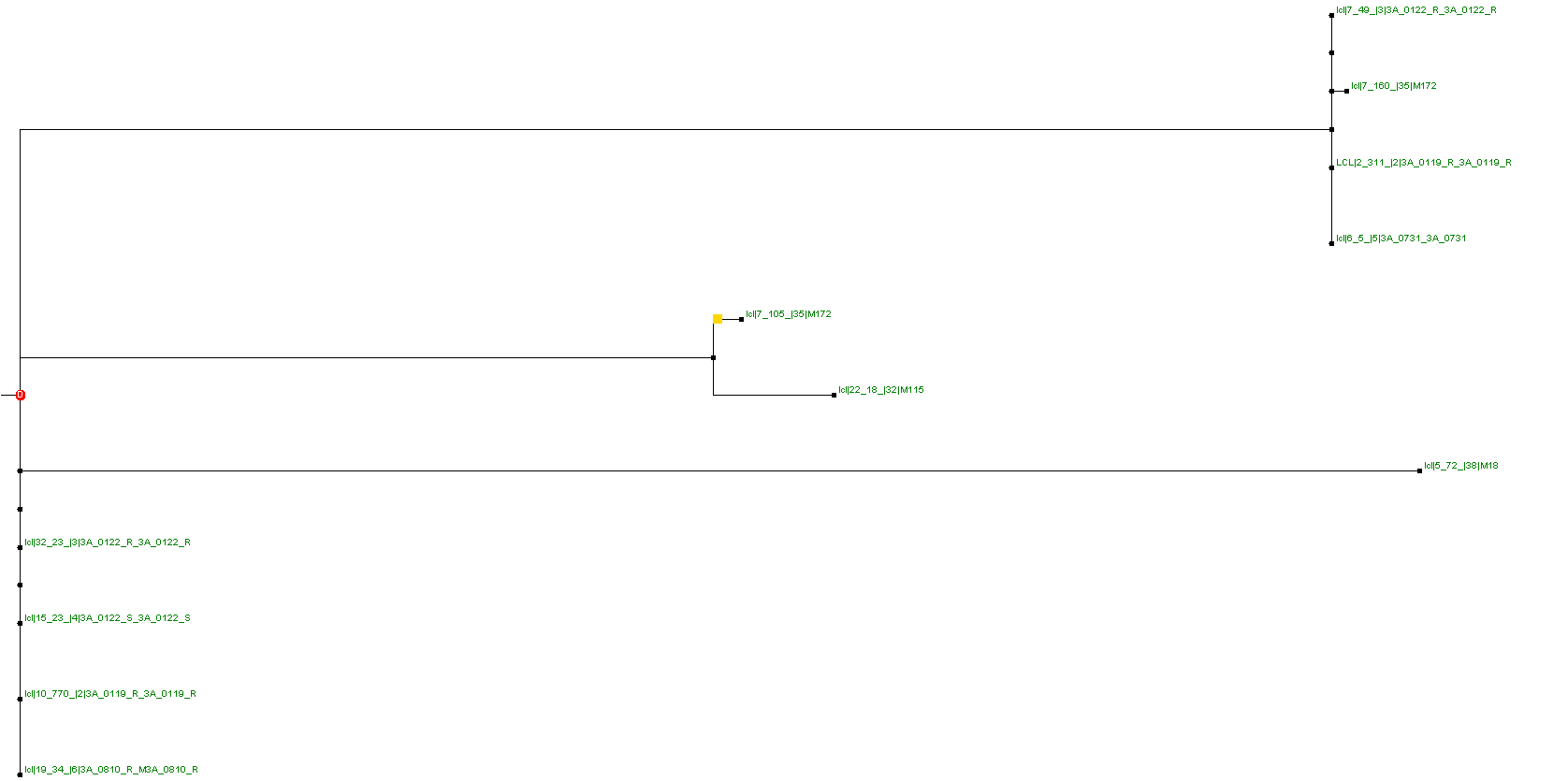

Supplement: Additional file 3 — The reconstructed trees for HGT events. Each tree contains one to six HGT events. The yellow squares represent the HGT event. [file 1745-6150-9-19-S3.zip › Tree_26.png]

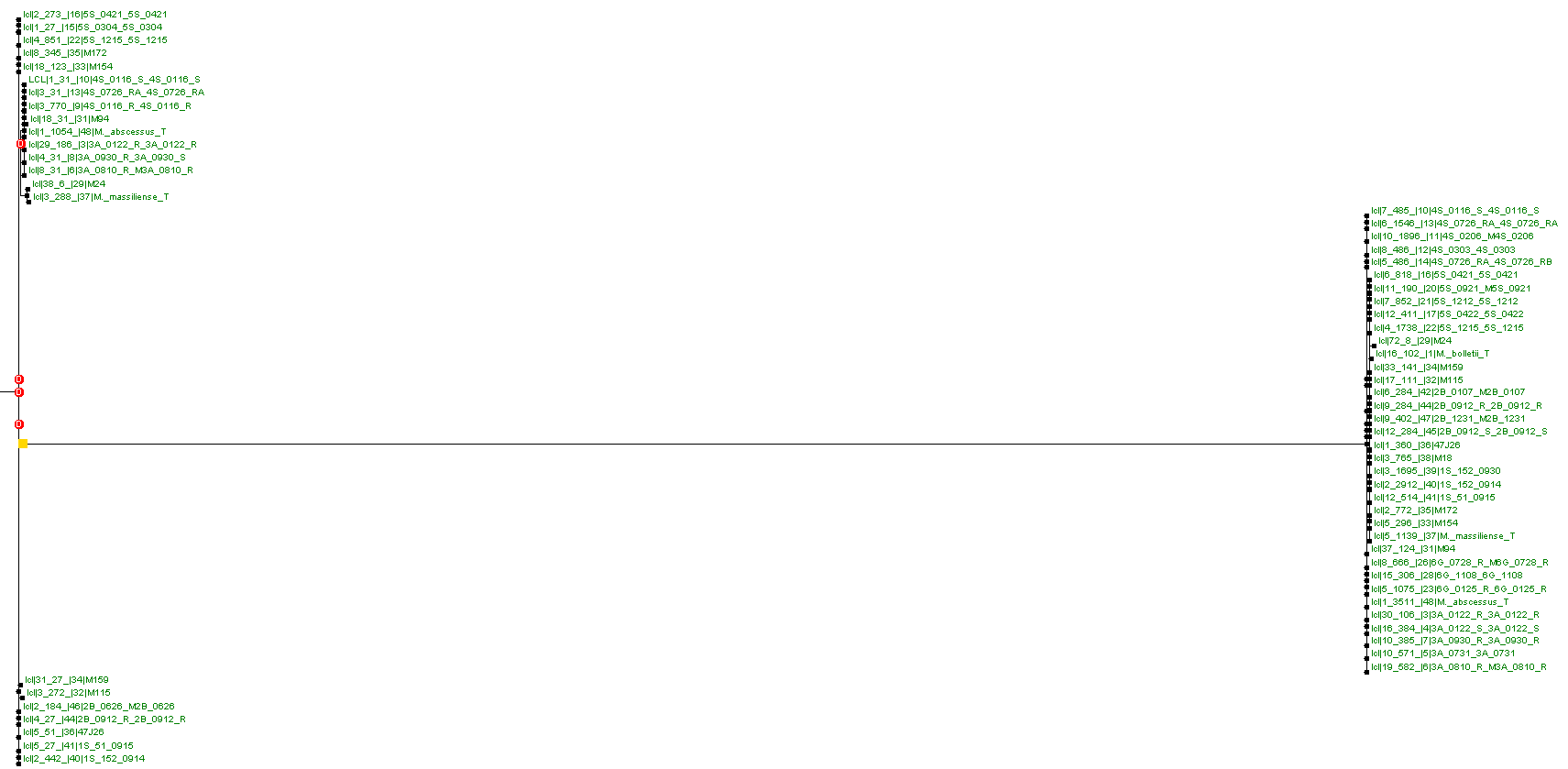

Supplement: Additional file 3 — The reconstructed trees for HGT events. Each tree contains one to six HGT events. The yellow squares represent the HGT event. [file 1745-6150-9-19-S3.zip › Tree_27.png]

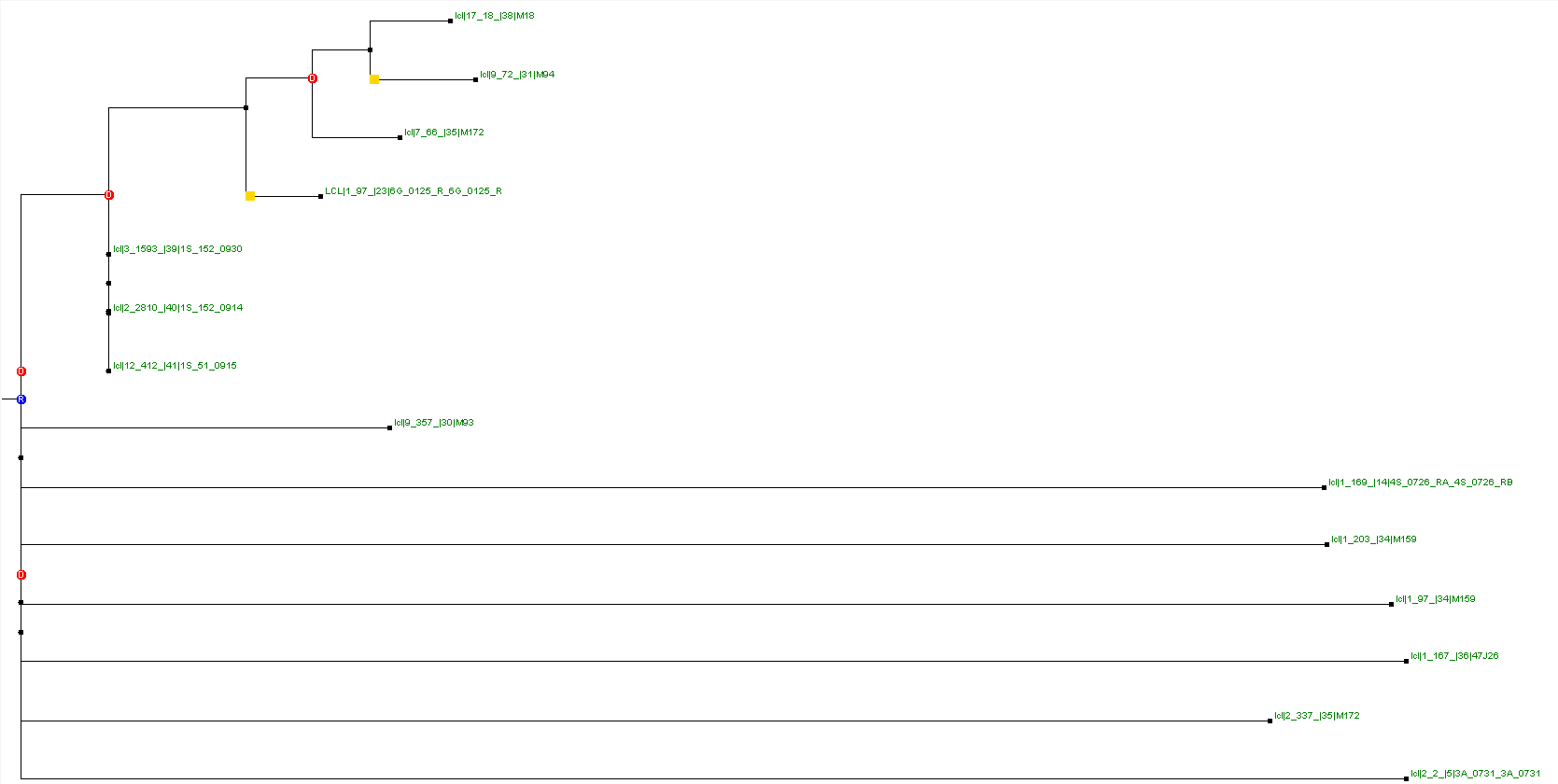

Supplement: Additional file 3 — The reconstructed trees for HGT events. Each tree contains one to six HGT events. The yellow squares represent the HGT event. [file 1745-6150-9-19-S3.zip › Tree_28.png]

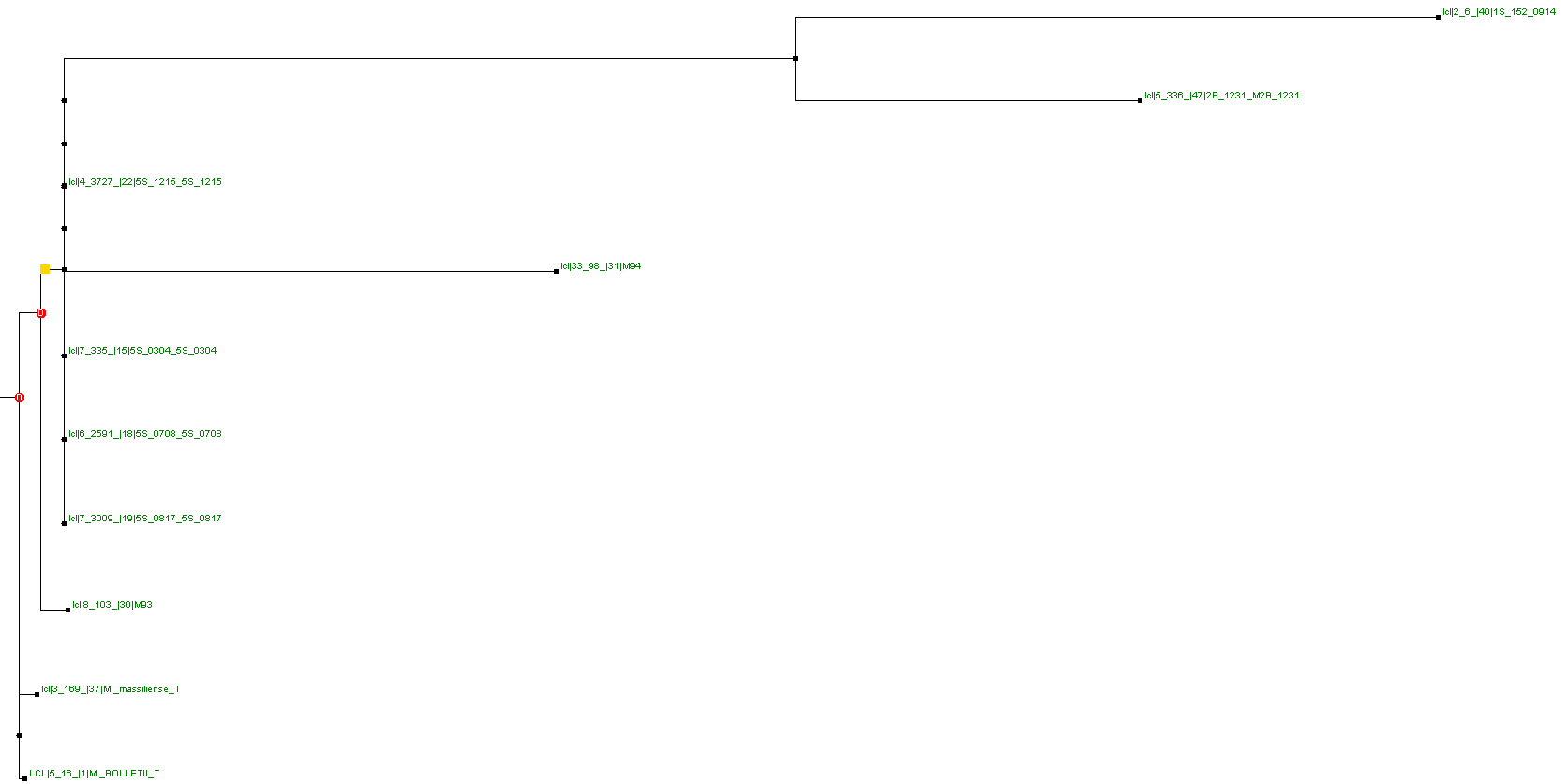

Supplement: Additional file 3 — The reconstructed trees for HGT events. Each tree contains one to six HGT events. The yellow squares represent the HGT event. [file 1745-6150-9-19-S3.zip › Tree_29.png]

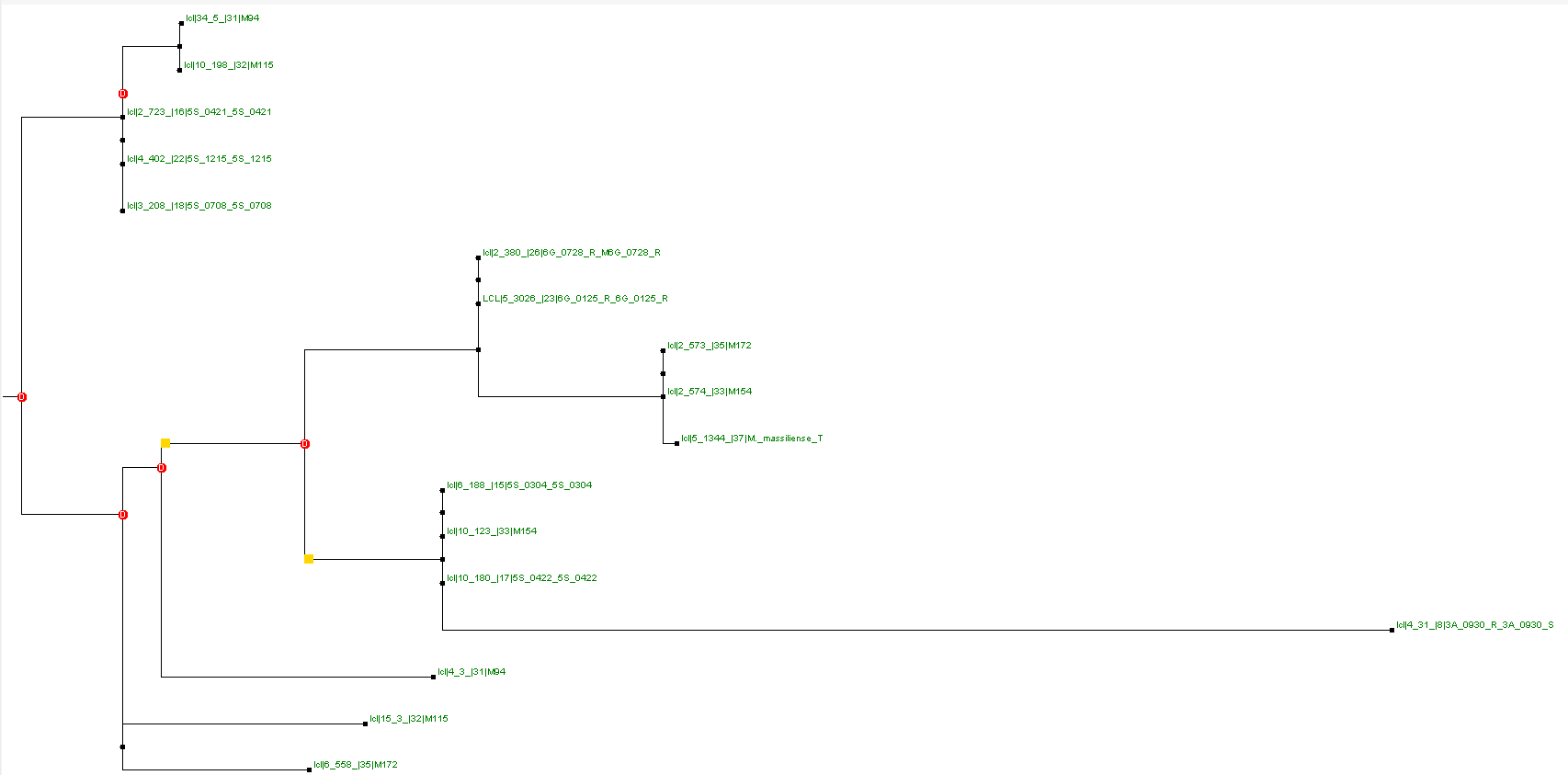

Supplement: Additional file 3 — The reconstructed trees for HGT events. Each tree contains one to six HGT events. The yellow squares represent the HGT event. [file 1745-6150-9-19-S3.zip › Tree_3.png]

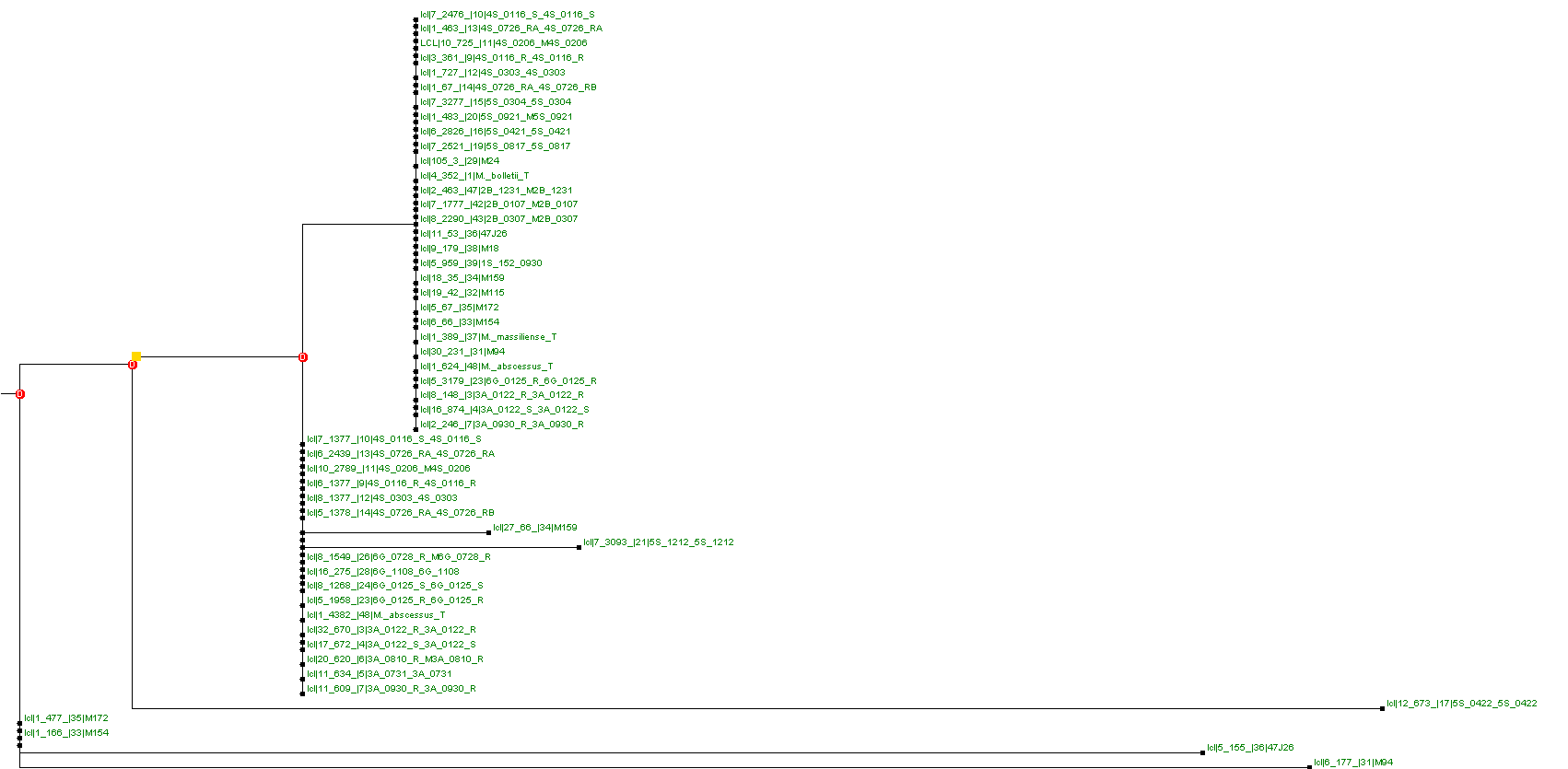

Supplement: Additional file 3 — The reconstructed trees for HGT events. Each tree contains one to six HGT events. The yellow squares represent the HGT event. [file 1745-6150-9-19-S3.zip › Tree_30.png]

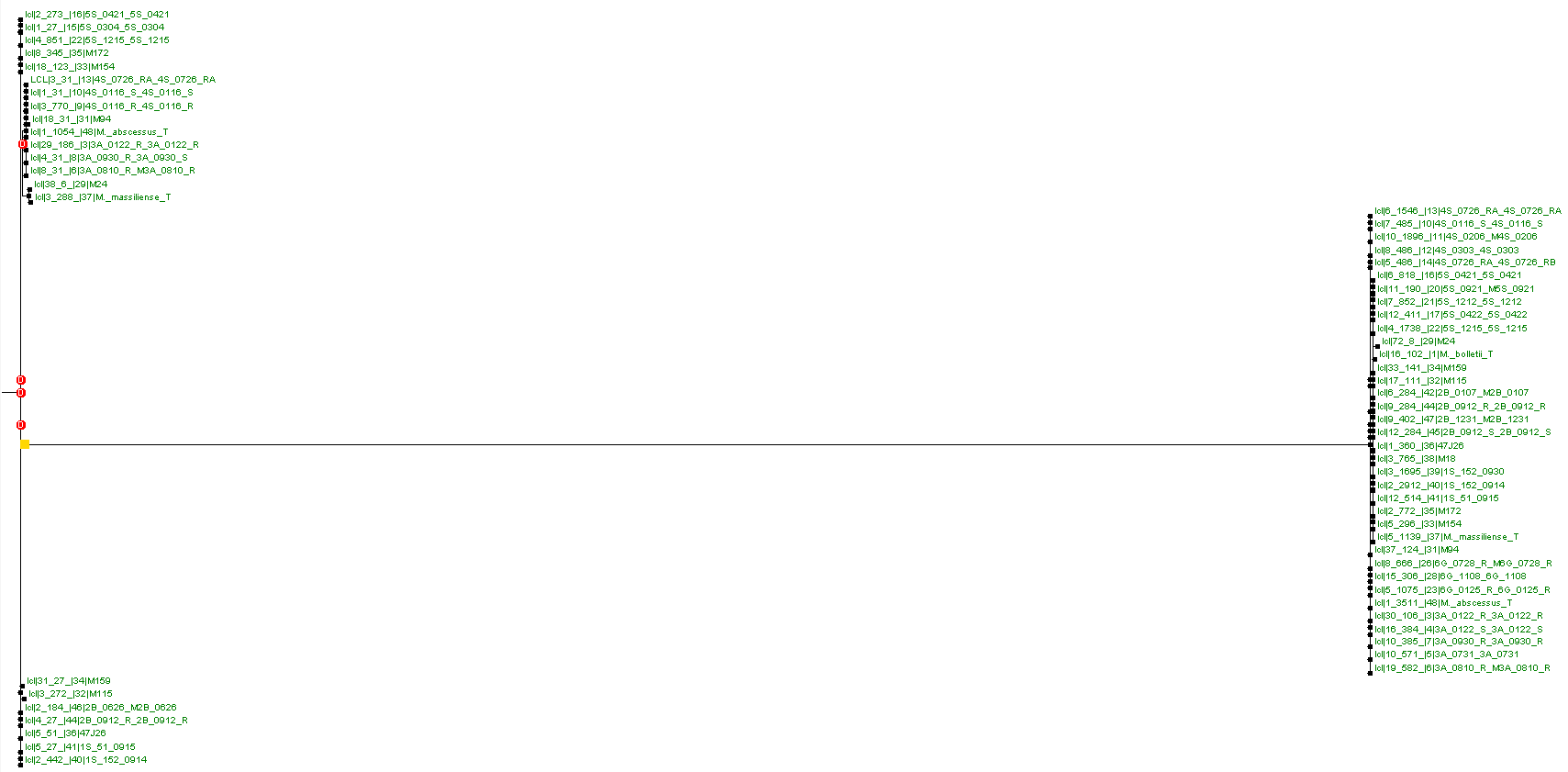

Supplement: Additional file 3 — The reconstructed trees for HGT events. Each tree contains one to six HGT events. The yellow squares represent the HGT event. [file 1745-6150-9-19-S3.zip › Tree_31.png]

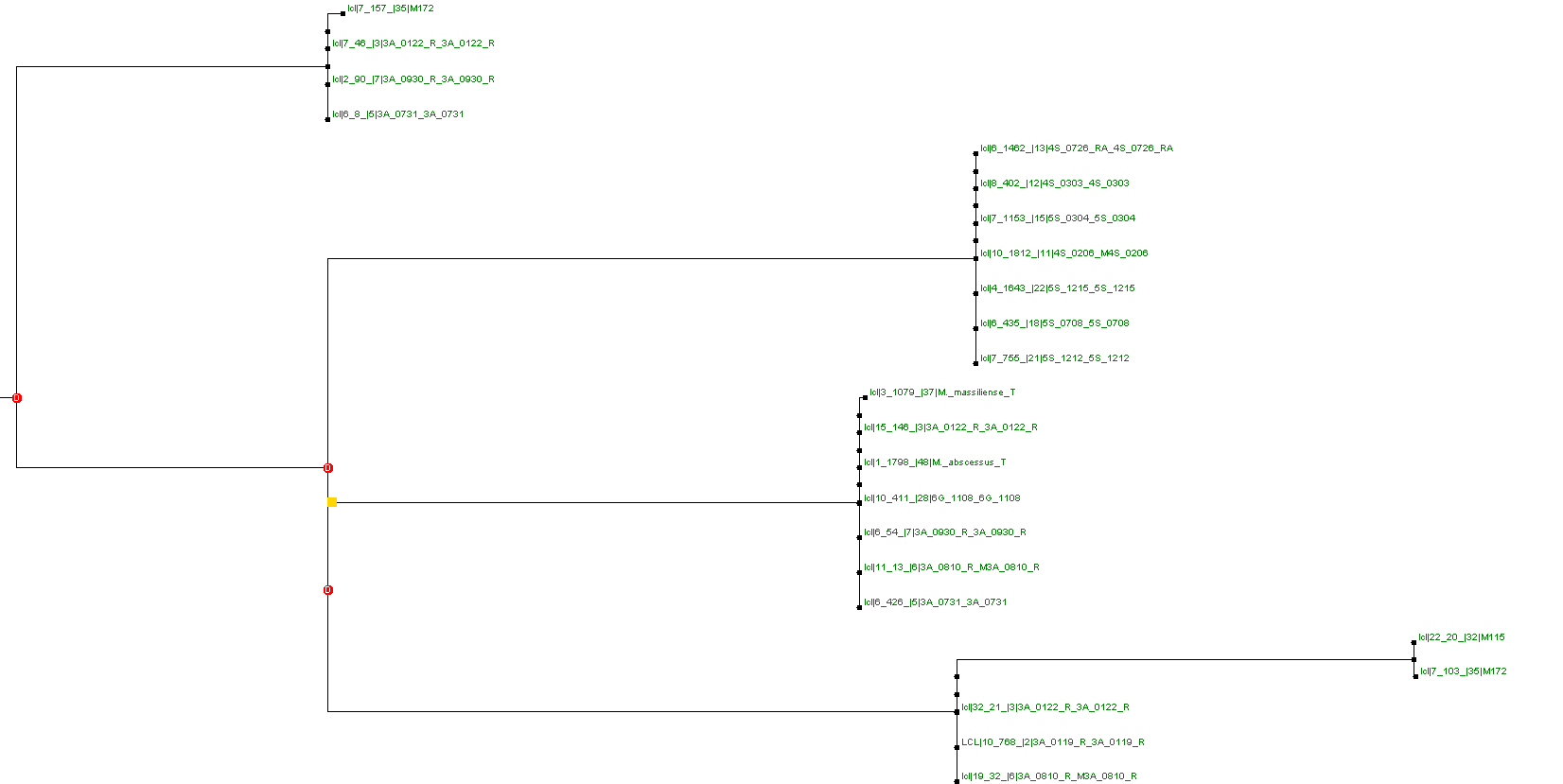

Supplement: Additional file 3 — The reconstructed trees for HGT events. Each tree contains one to six HGT events. The yellow squares represent the HGT event. [file 1745-6150-9-19-S3.zip › Tree_32.png]

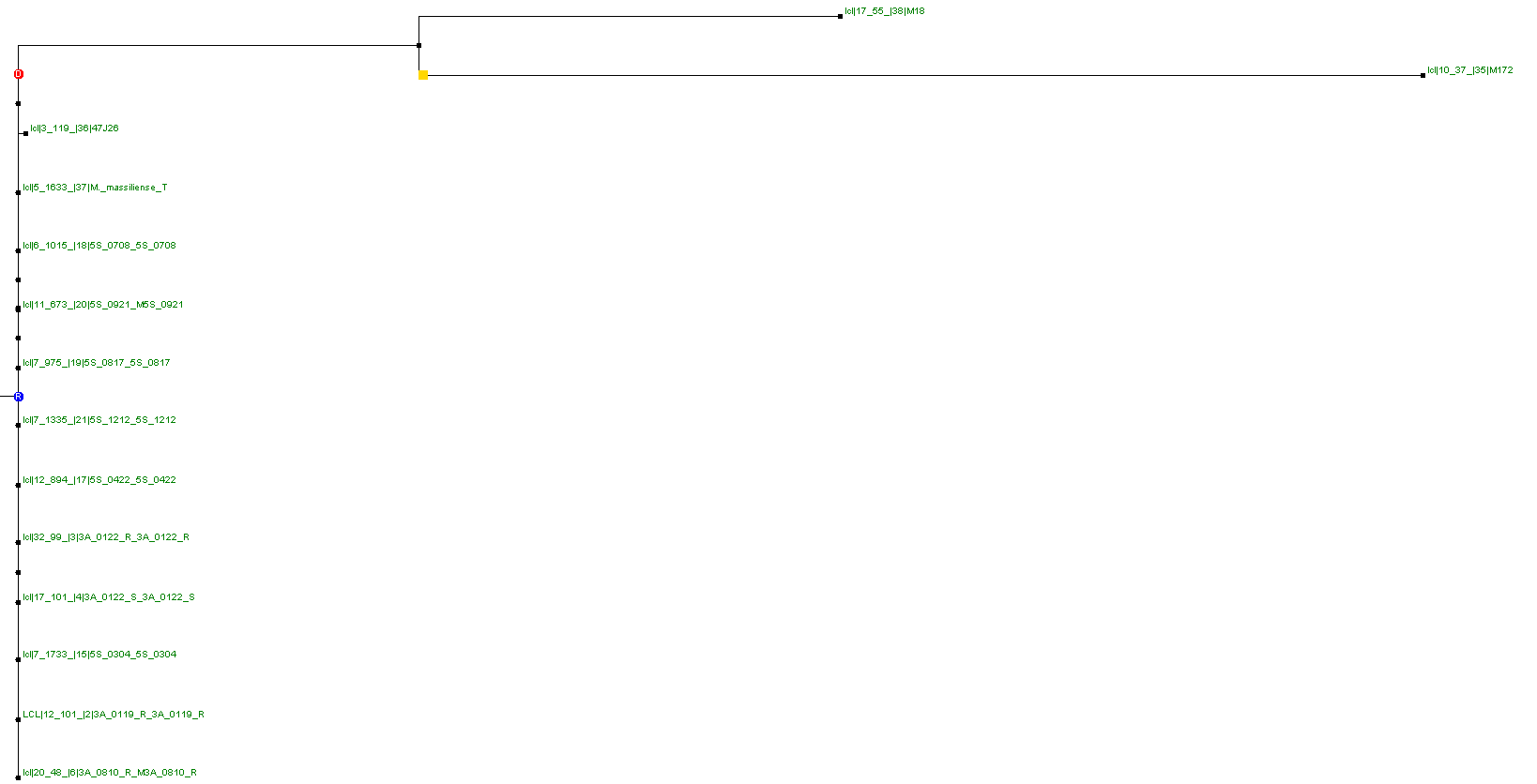

Supplement: Additional file 3 — The reconstructed trees for HGT events. Each tree contains one to six HGT events. The yellow squares represent the HGT event. [file 1745-6150-9-19-S3.zip › Tree_33.png]

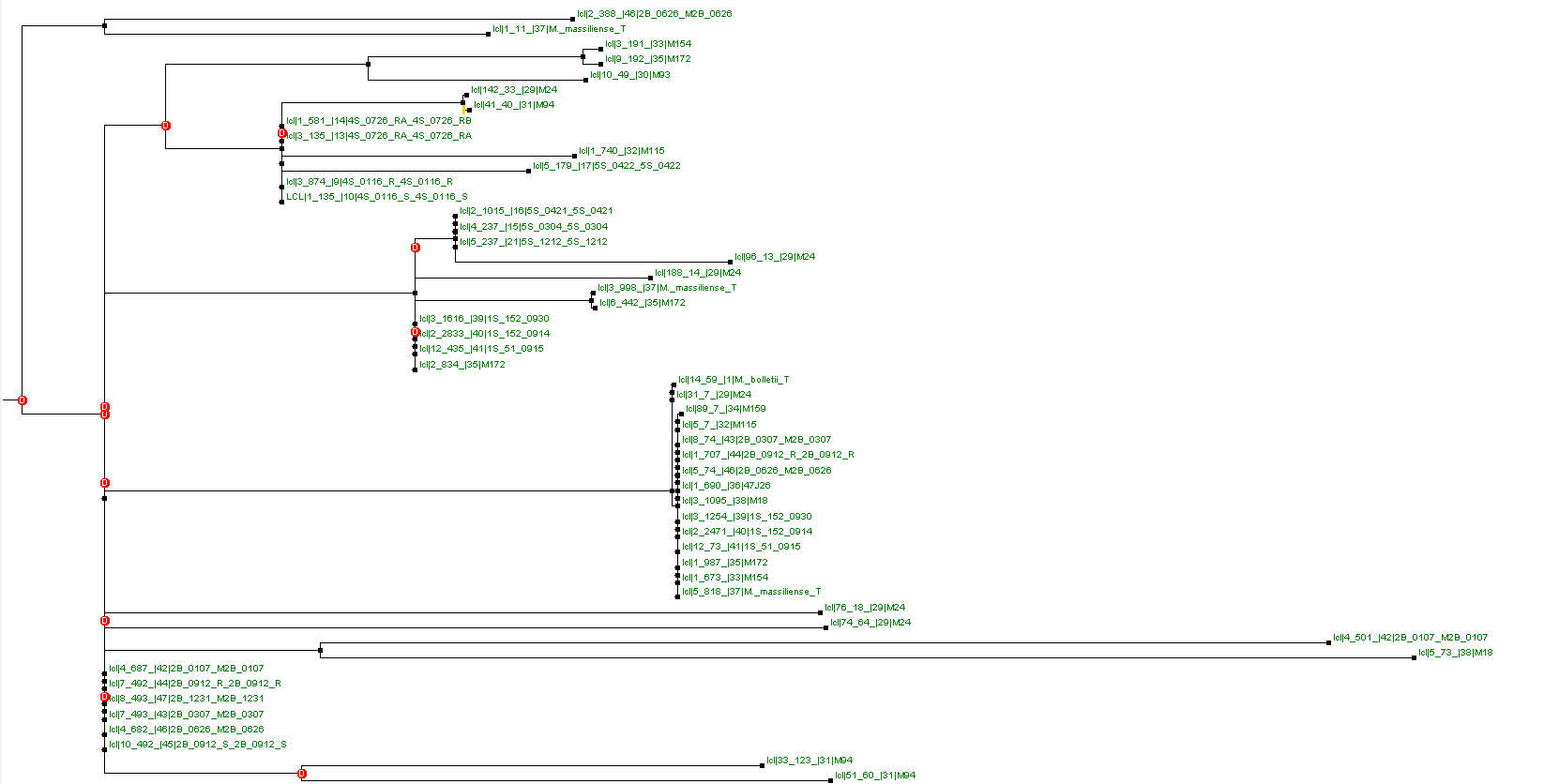

Supplement: Additional file 3 — The reconstructed trees for HGT events. Each tree contains one to six HGT events. The yellow squares represent the HGT event. [file 1745-6150-9-19-S3.zip › Tree_34.png]

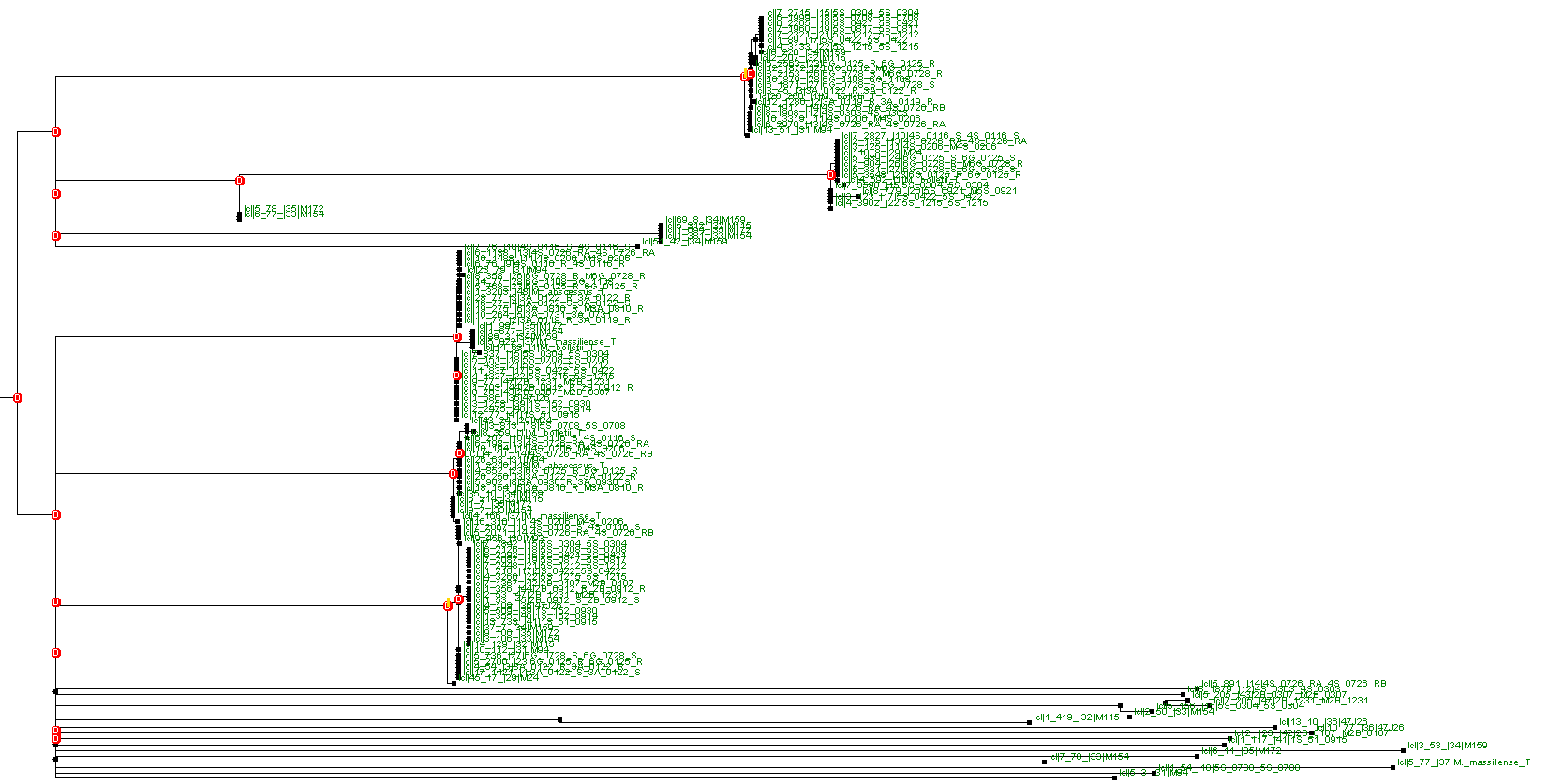

Supplement: Additional file 3 — The reconstructed trees for HGT events. Each tree contains one to six HGT events. The yellow squares represent the HGT event. [file 1745-6150-9-19-S3.zip › Tree_35.png]

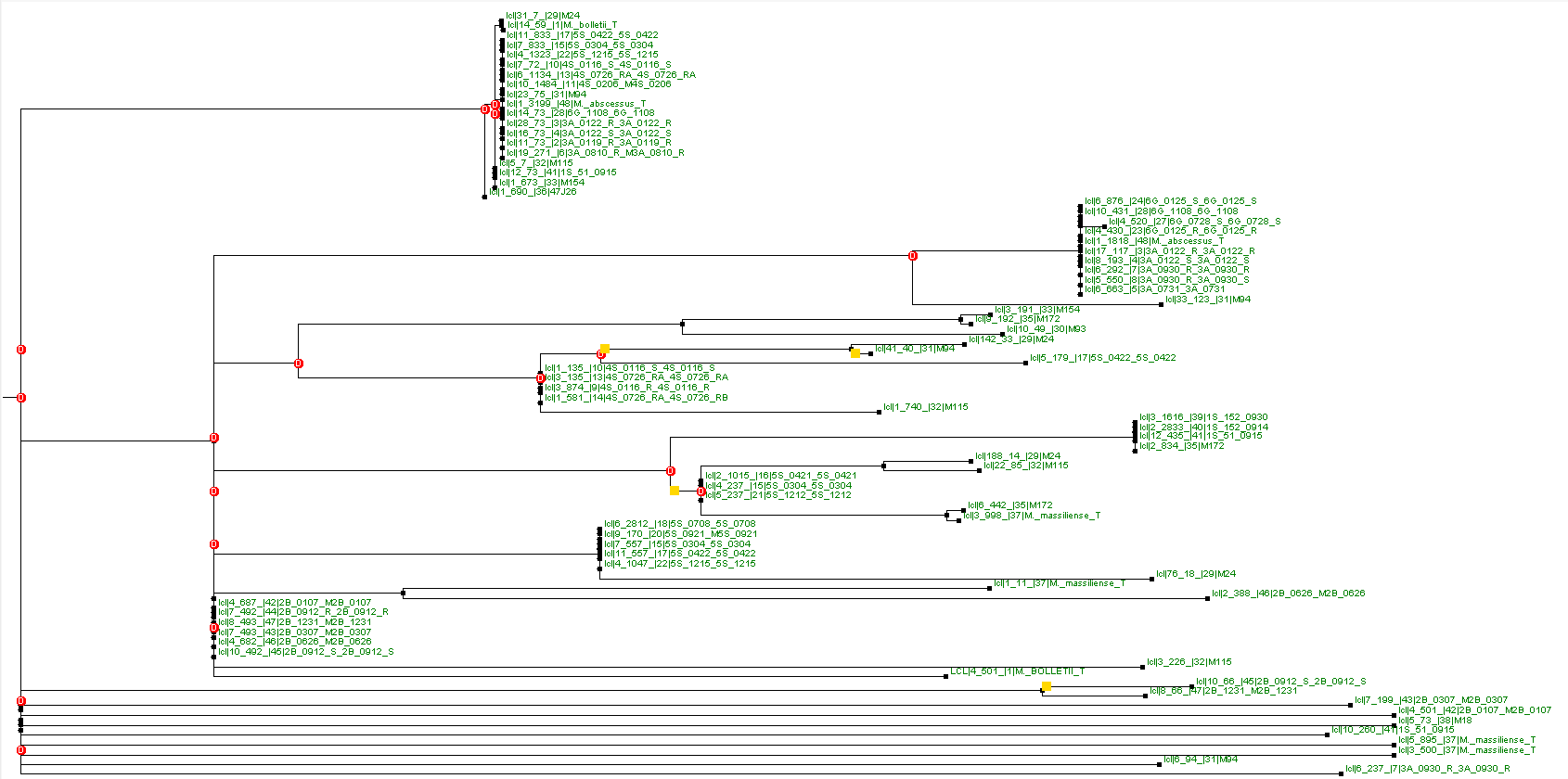

Supplement: Additional file 3 — The reconstructed trees for HGT events. Each tree contains one to six HGT events. The yellow squares represent the HGT event. [file 1745-6150-9-19-S3.zip › Tree_36.png]

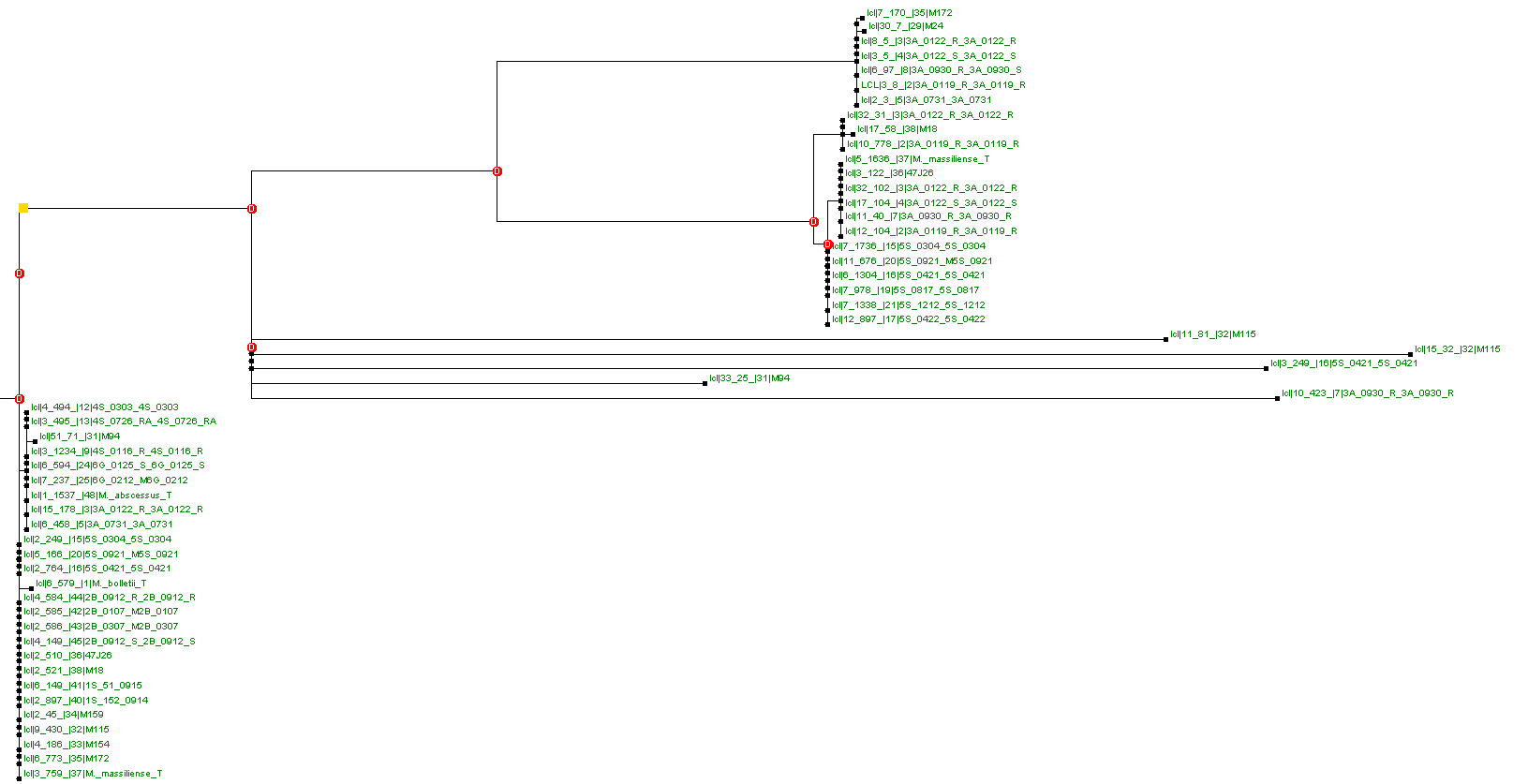

Supplement: Additional file 3 — The reconstructed trees for HGT events. Each tree contains one to six HGT events. The yellow squares represent the HGT event. [file 1745-6150-9-19-S3.zip › Tree_37.png]

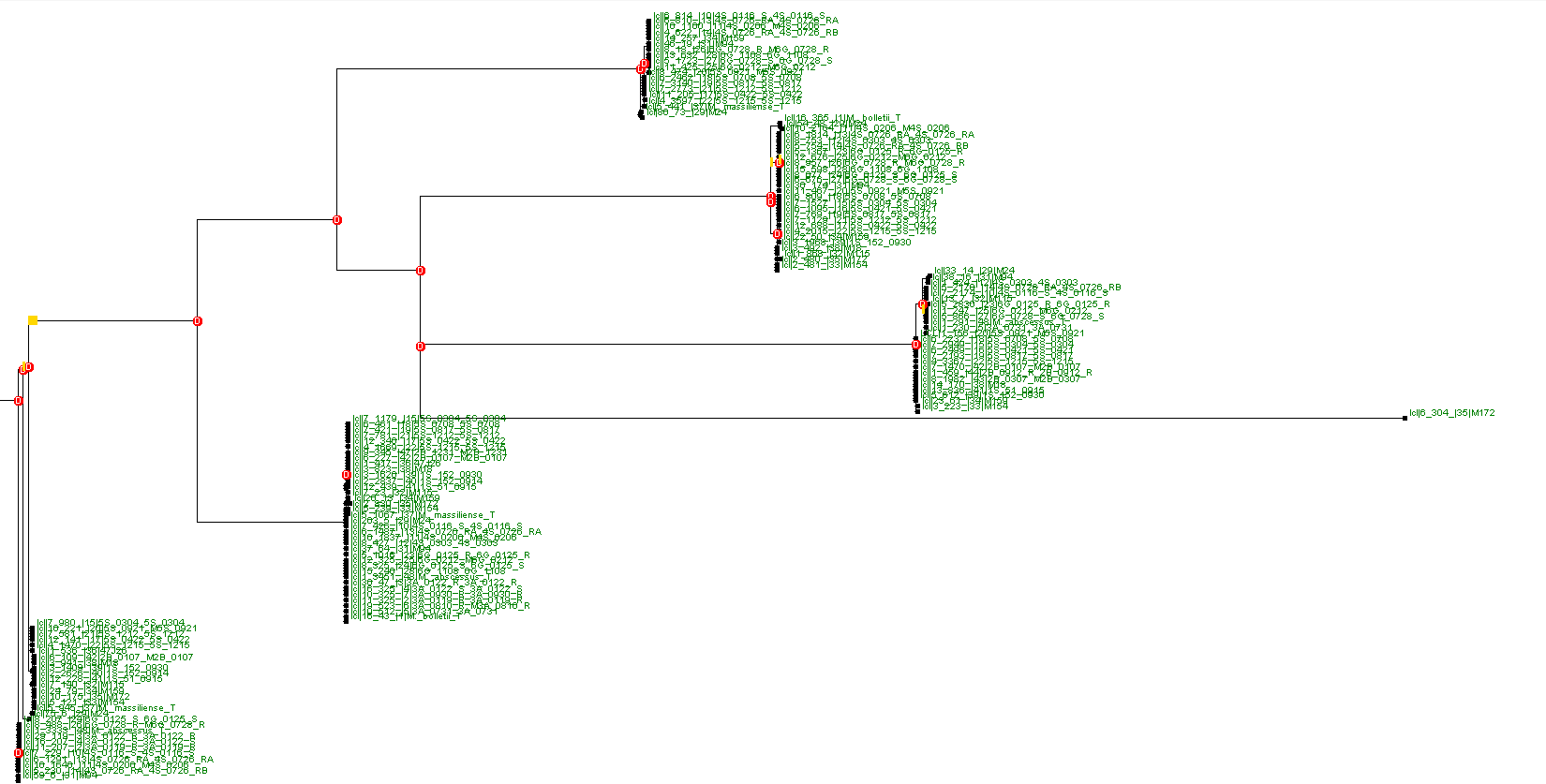

Supplement: Additional file 3 — The reconstructed trees for HGT events. Each tree contains one to six HGT events. The yellow squares represent the HGT event. [file 1745-6150-9-19-S3.zip › Tree_38.png]

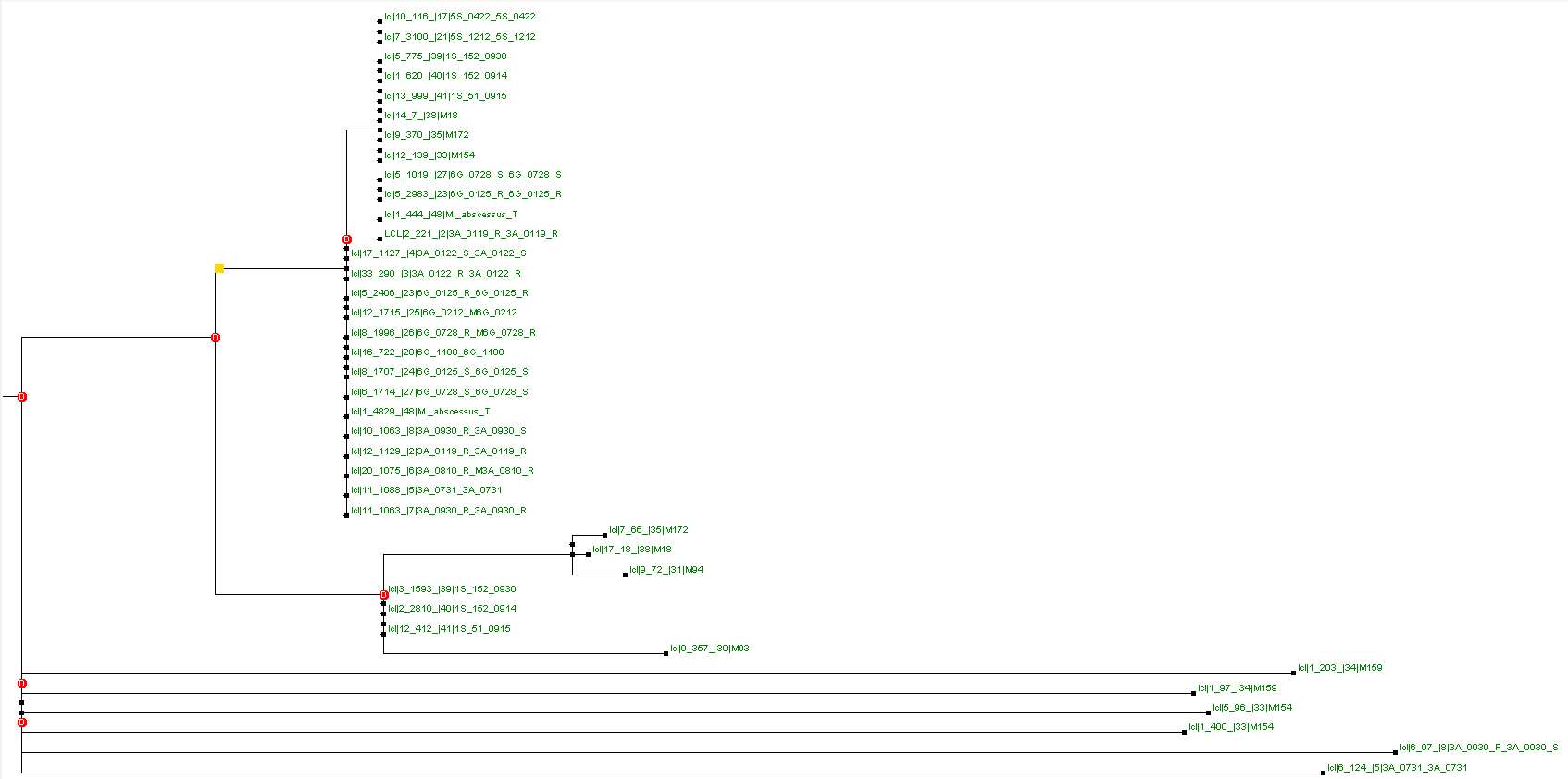

Supplement: Additional file 3 — The reconstructed trees for HGT events. Each tree contains one to six HGT events. The yellow squares represent the HGT event. [file 1745-6150-9-19-S3.zip › Tree_39.png]

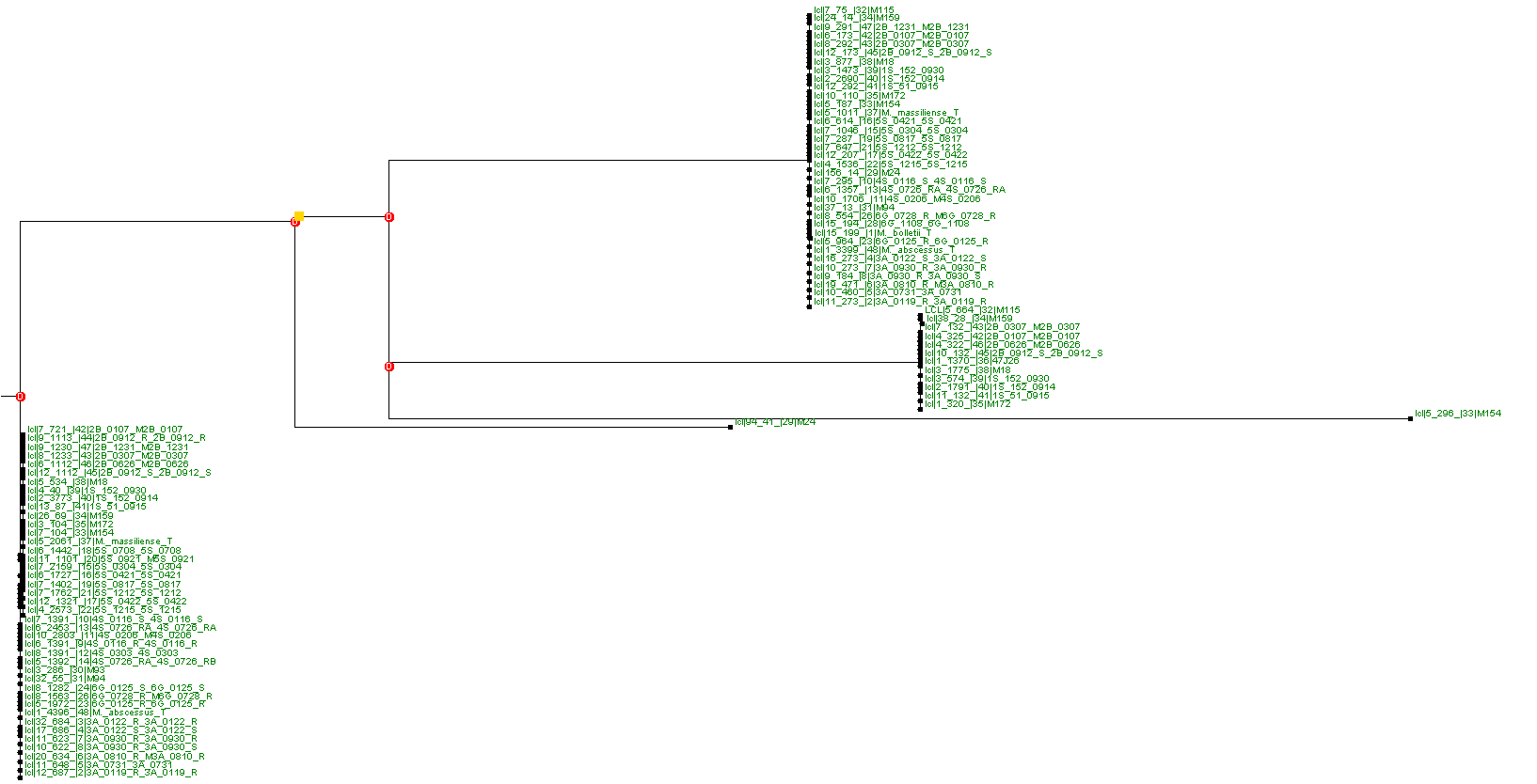

Supplement: Additional file 3 — The reconstructed trees for HGT events. Each tree contains one to six HGT events. The yellow squares represent the HGT event. [file 1745-6150-9-19-S3.zip › Tree_4.png]

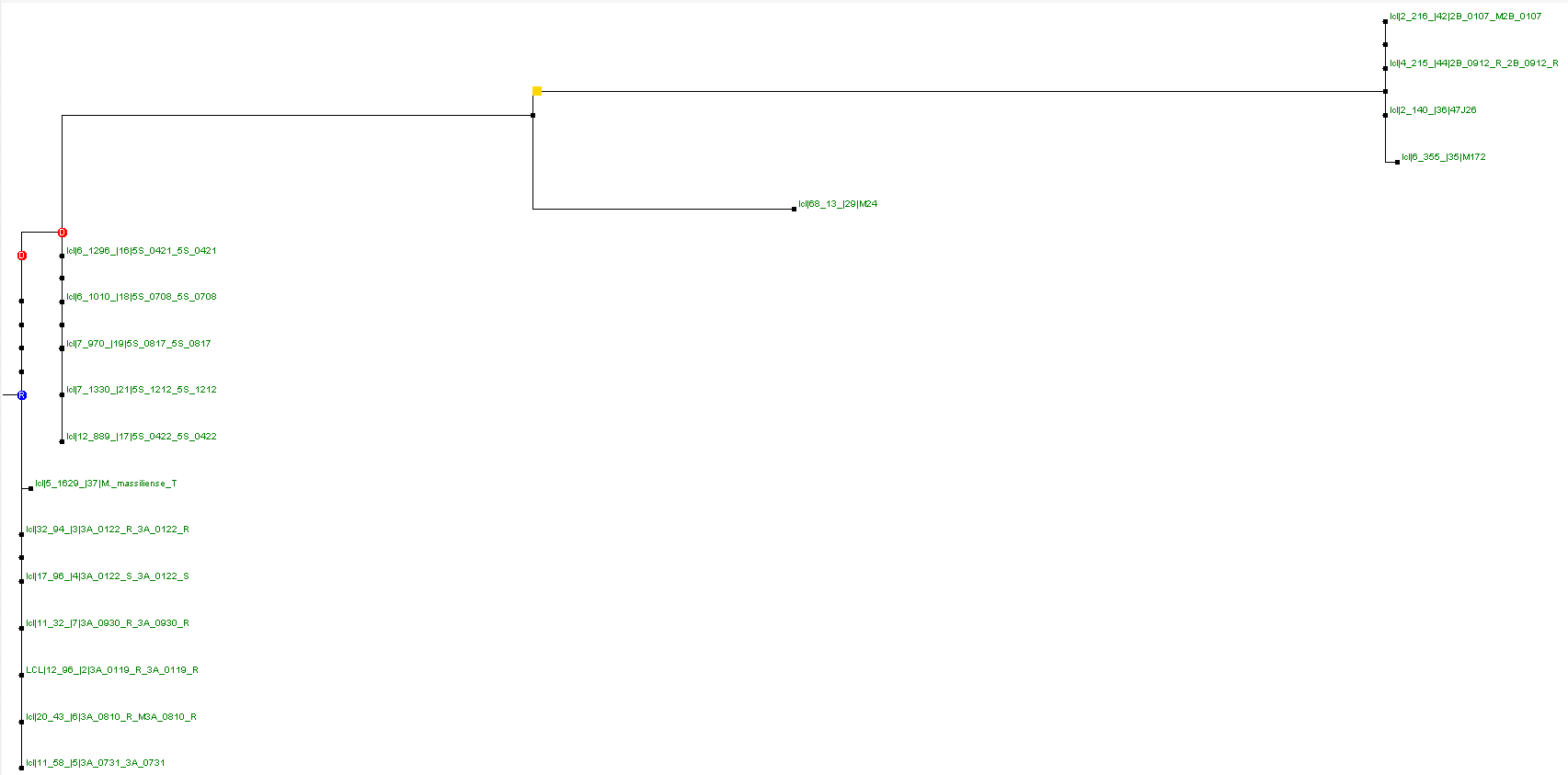

Supplement: Additional file 3 — The reconstructed trees for HGT events. Each tree contains one to six HGT events. The yellow squares represent the HGT event. [file 1745-6150-9-19-S3.zip › Tree_40.png]

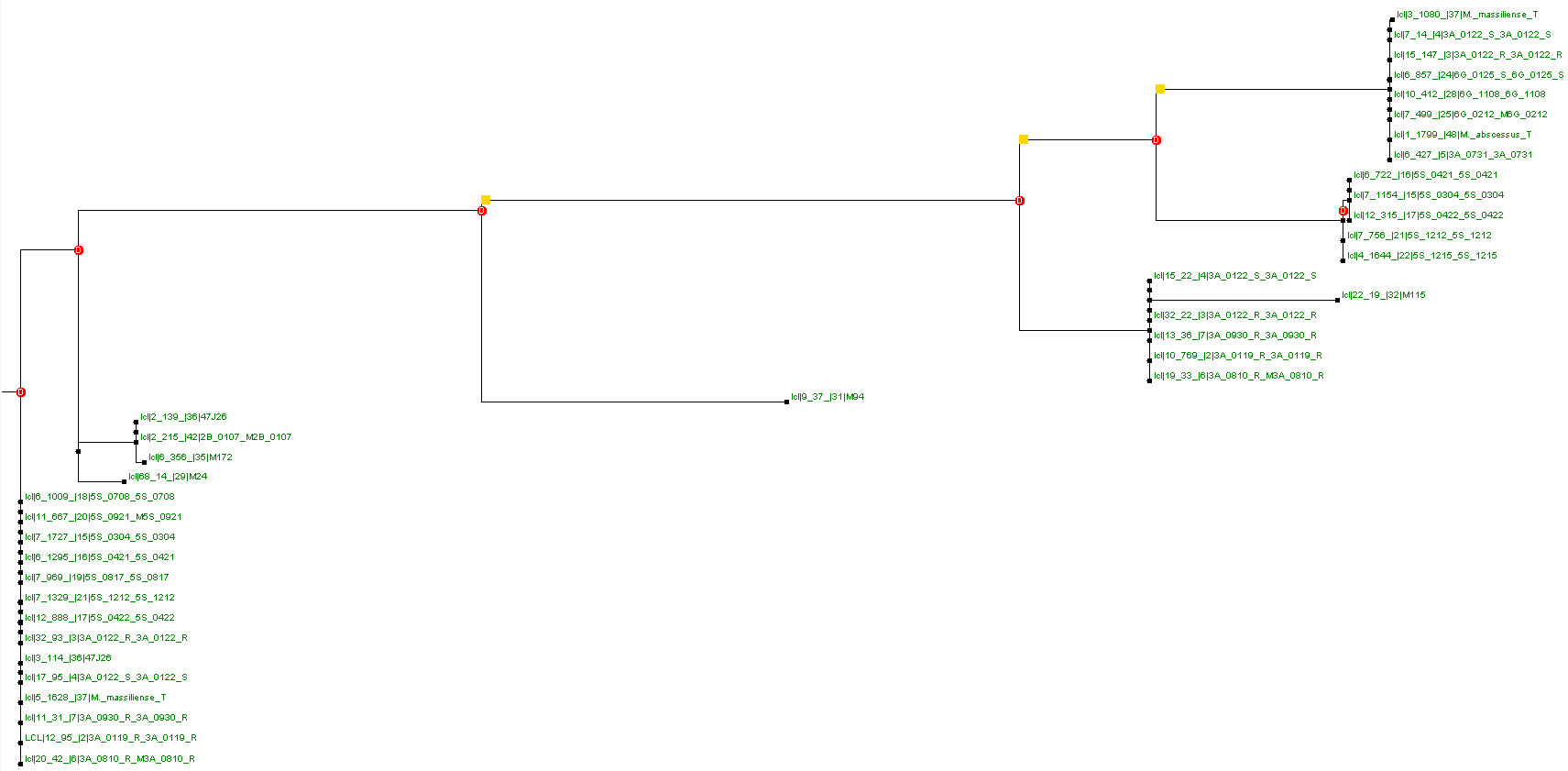

Supplement: Additional file 3 — The reconstructed trees for HGT events. Each tree contains one to six HGT events. The yellow squares represent the HGT event. [file 1745-6150-9-19-S3.zip › Tree_41.png]

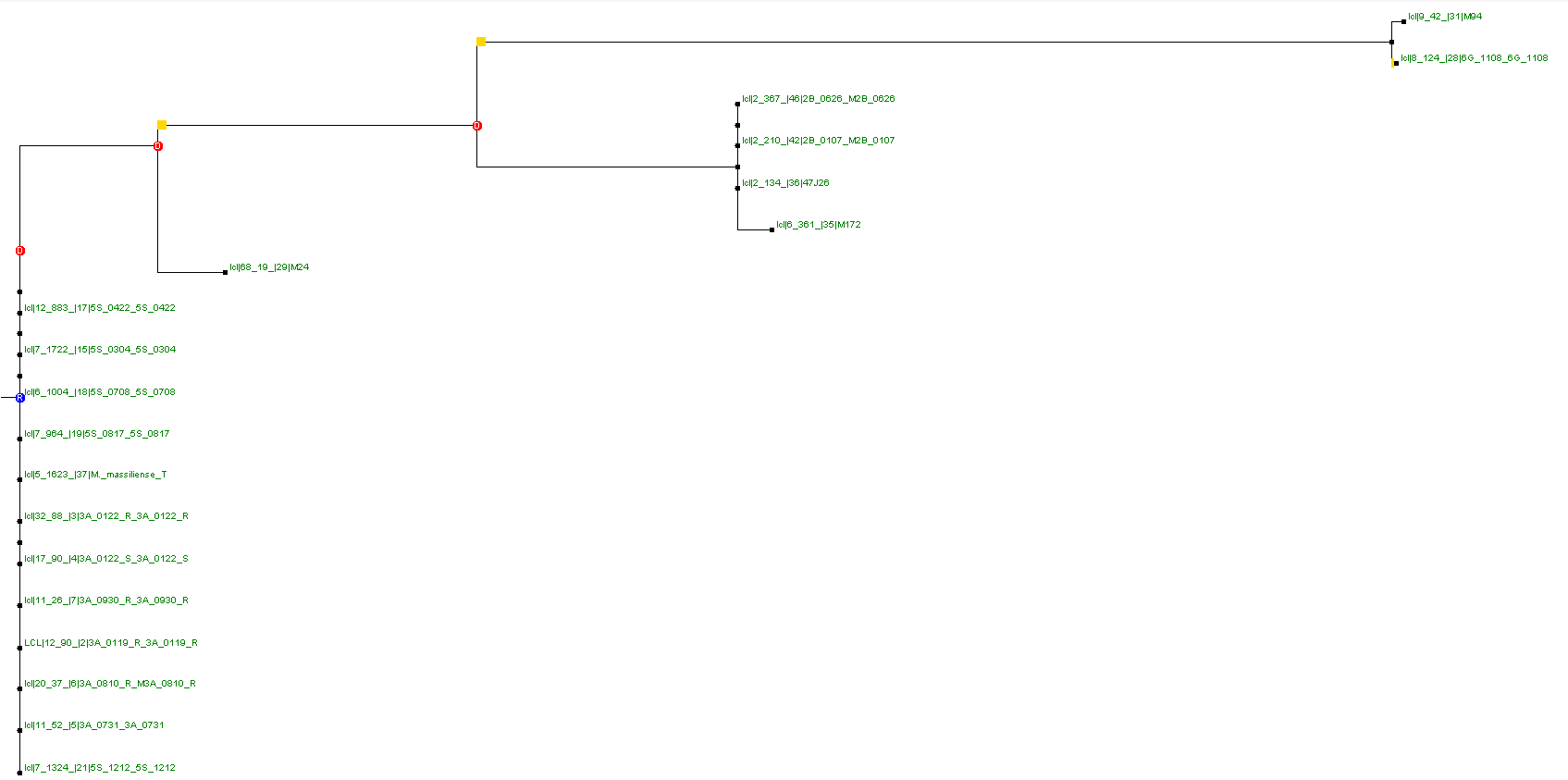

Supplement: Additional file 3 — The reconstructed trees for HGT events. Each tree contains one to six HGT events. The yellow squares represent the HGT event. [file 1745-6150-9-19-S3.zip › Tree_42.png]

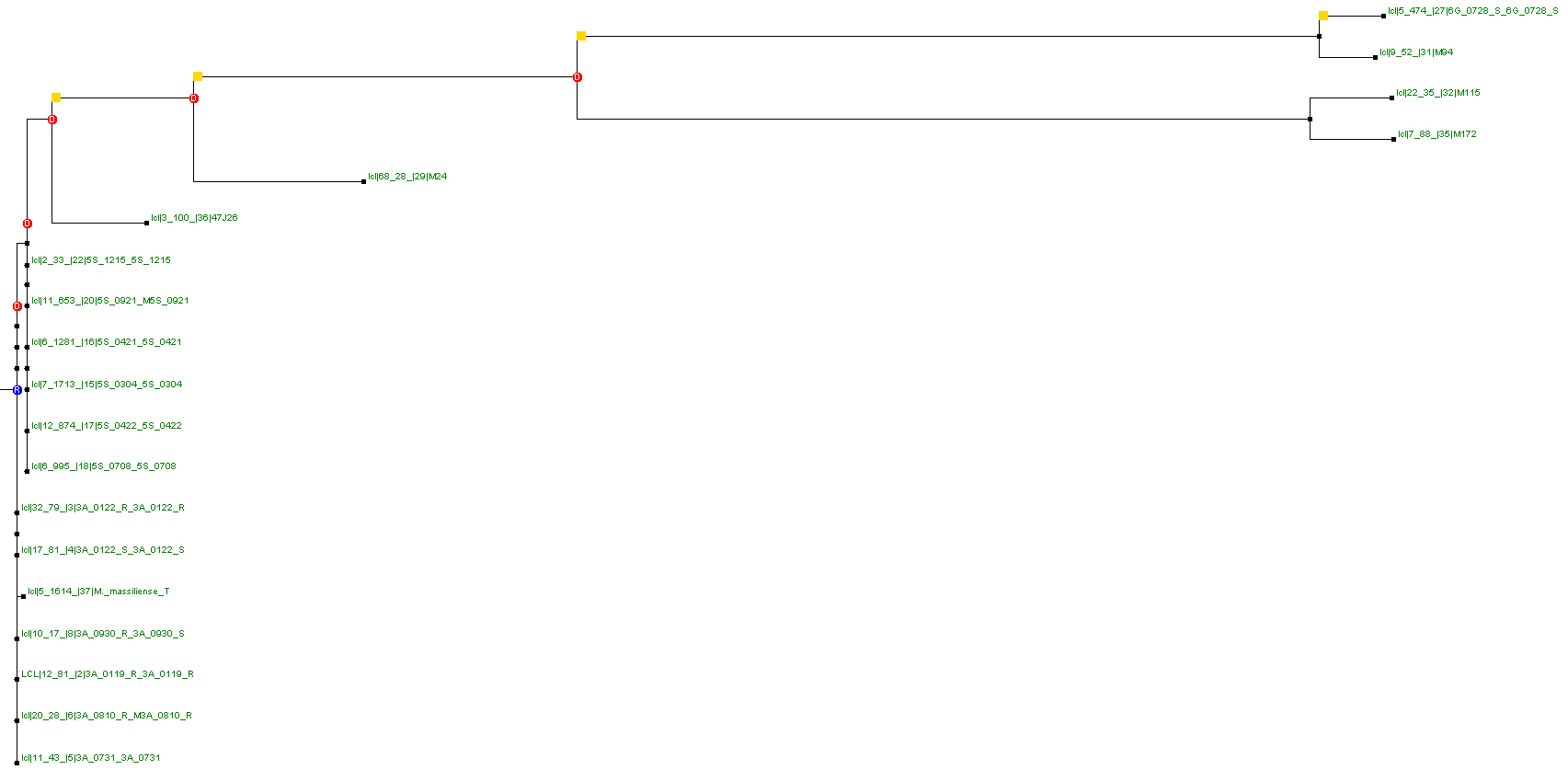

Supplement: Additional file 3 — The reconstructed trees for HGT events. Each tree contains one to six HGT events. The yellow squares represent the HGT event. [file 1745-6150-9-19-S3.zip › Tree_43.png]

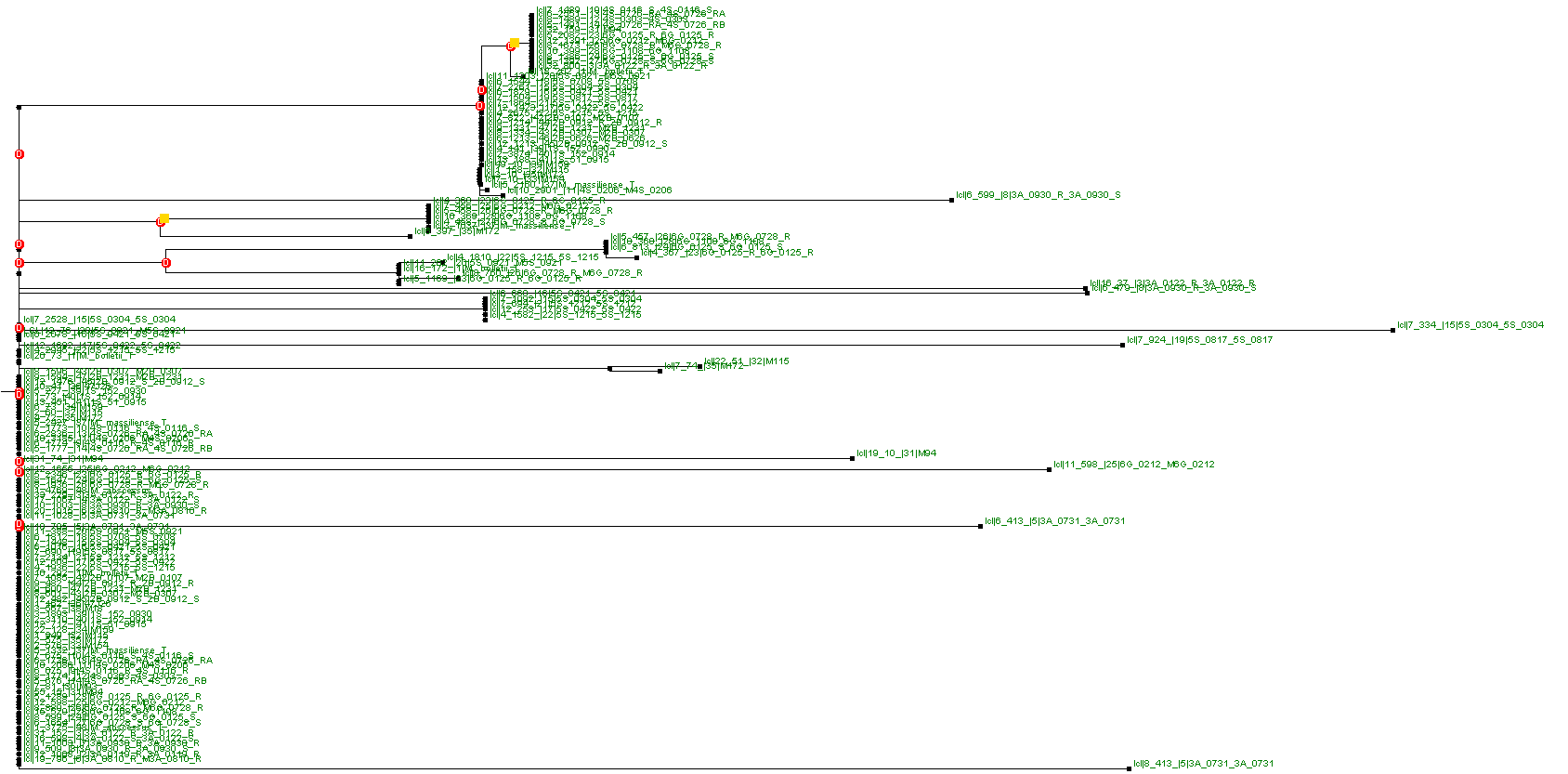

Supplement: Additional file 3 — The reconstructed trees for HGT events. Each tree contains one to six HGT events. The yellow squares represent the HGT event. [file 1745-6150-9-19-S3.zip › Tree_44.png]

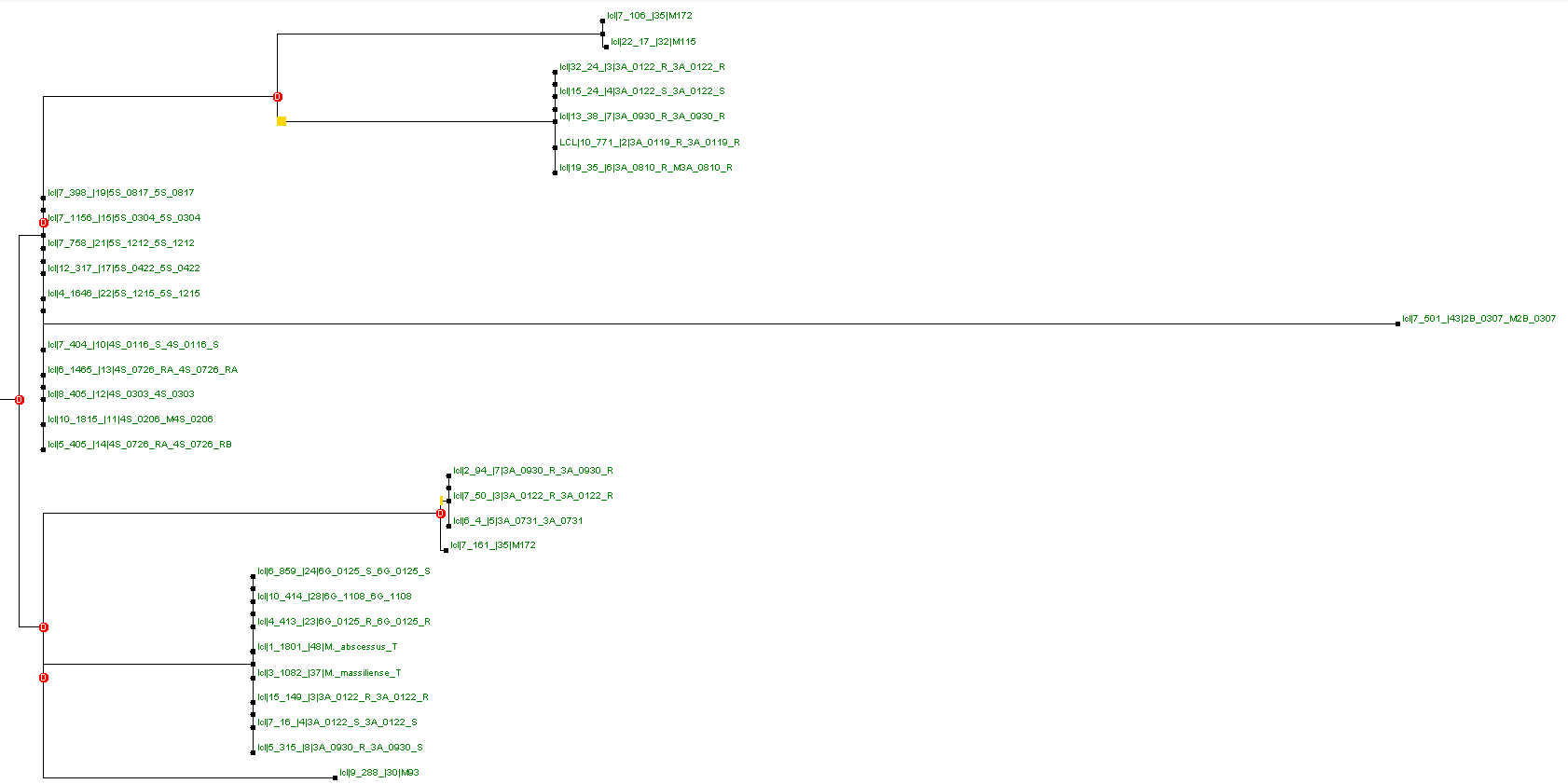

Supplement: Additional file 3 — The reconstructed trees for HGT events. Each tree contains one to six HGT events. The yellow squares represent the HGT event. [file 1745-6150-9-19-S3.zip › Tree_45.png]

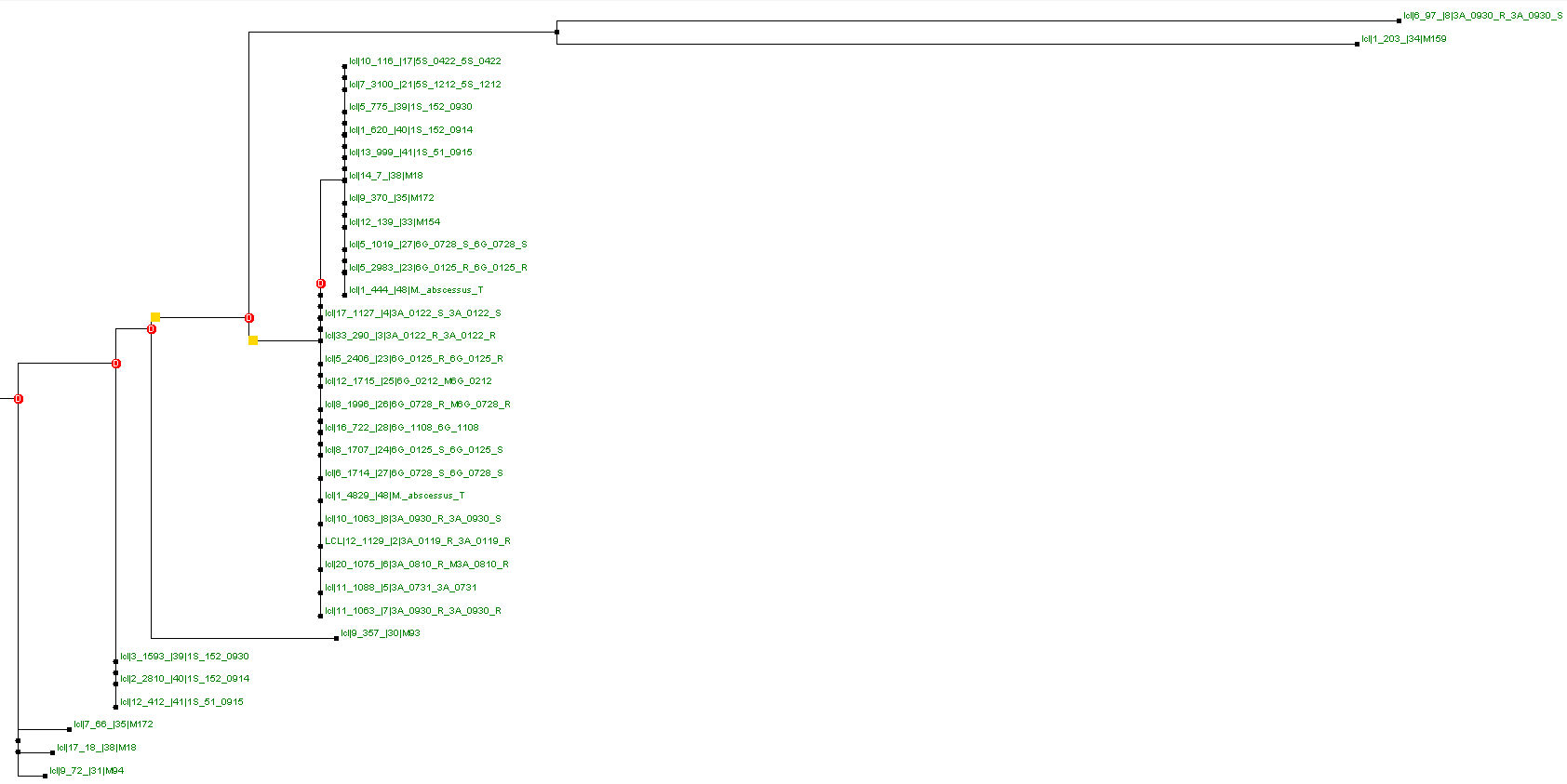

Supplement: Additional file 3 — The reconstructed trees for HGT events. Each tree contains one to six HGT events. The yellow squares represent the HGT event. [file 1745-6150-9-19-S3.zip › Tree_46.png]

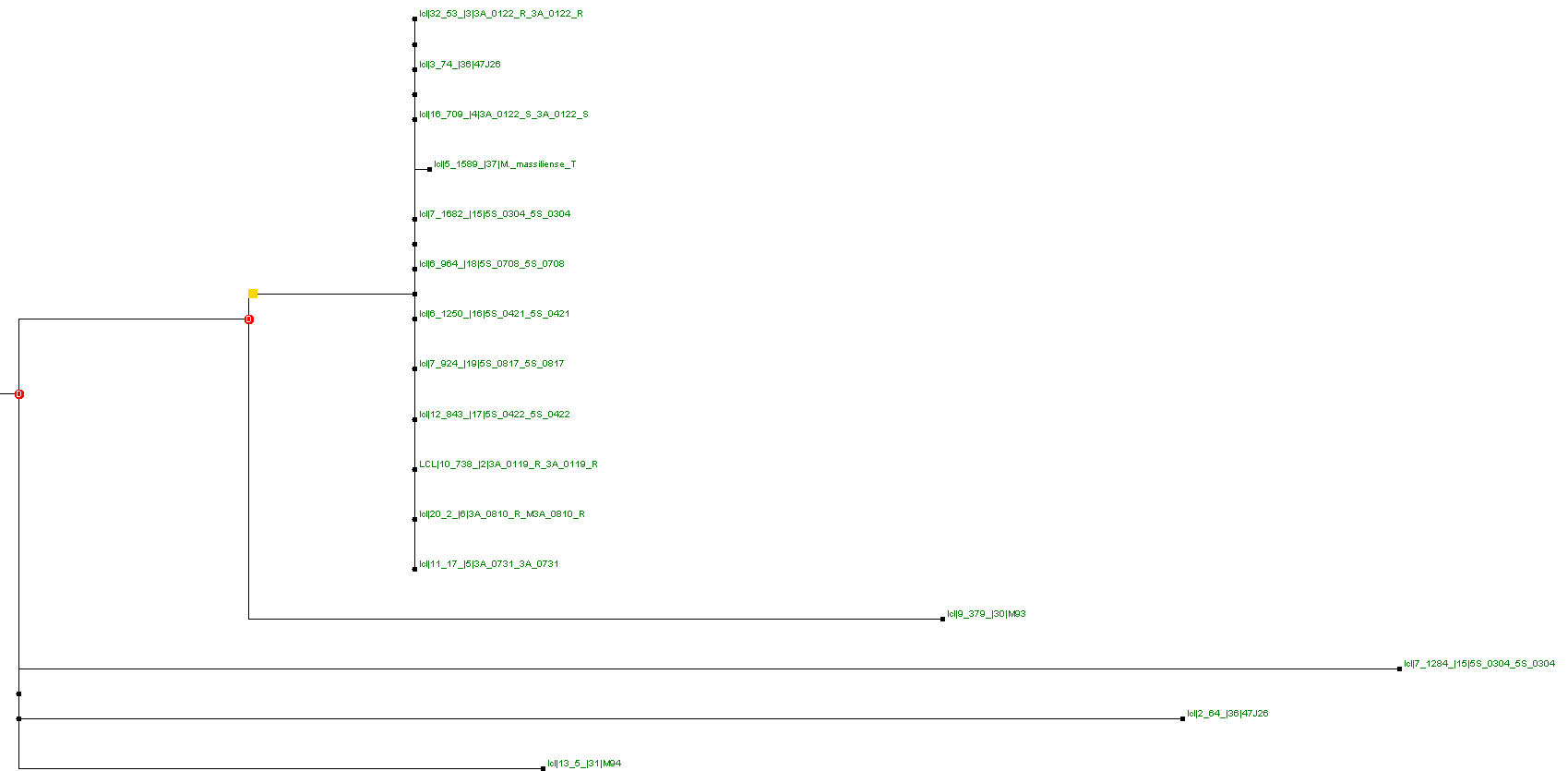

Supplement: Additional file 3 — The reconstructed trees for HGT events. Each tree contains one to six HGT events. The yellow squares represent the HGT event. [file 1745-6150-9-19-S3.zip › Tree_47.png]

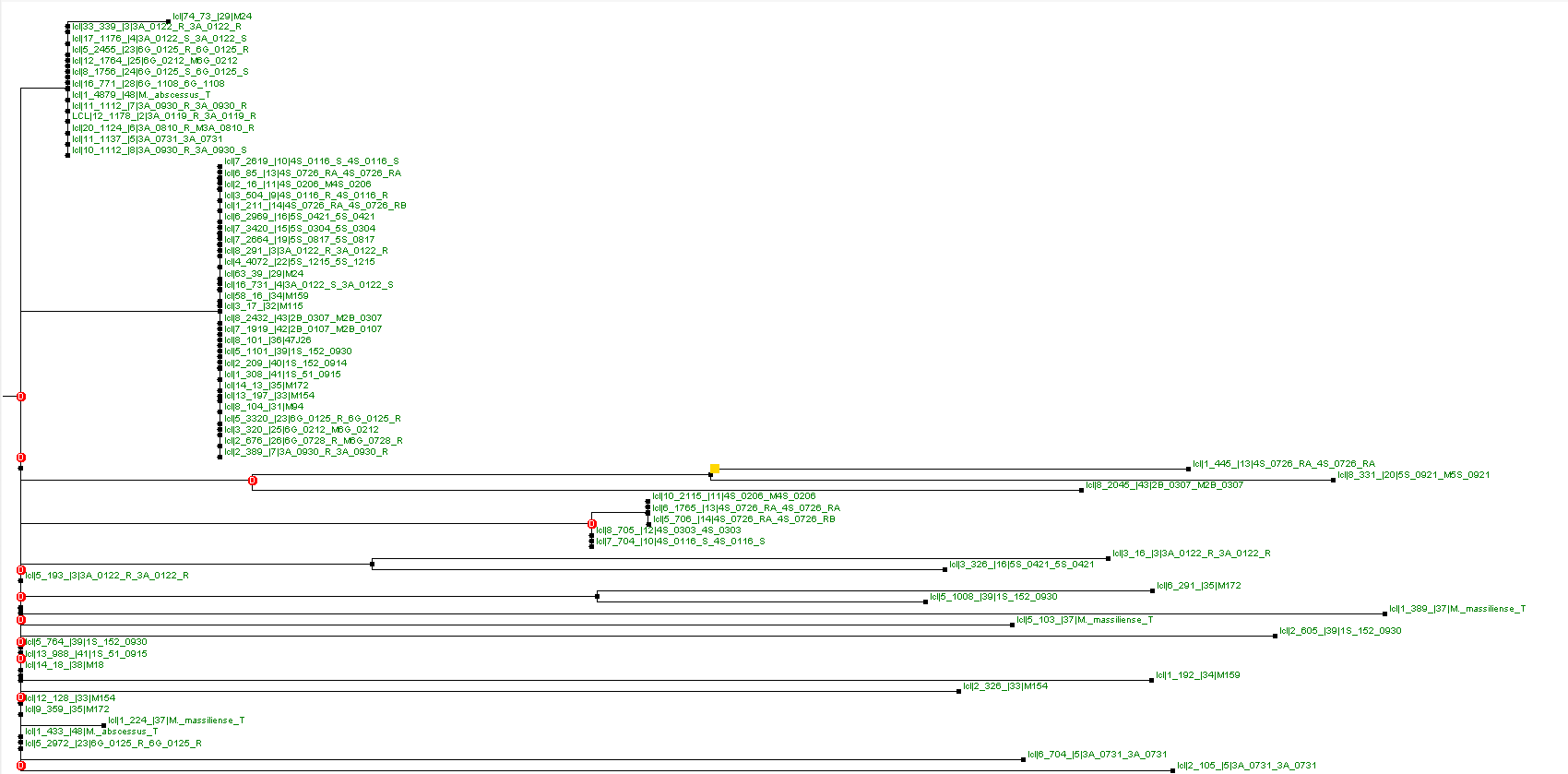

Supplement: Additional file 3 — The reconstructed trees for HGT events. Each tree contains one to six HGT events. The yellow squares represent the HGT event. [file 1745-6150-9-19-S3.zip › Tree_48.png]

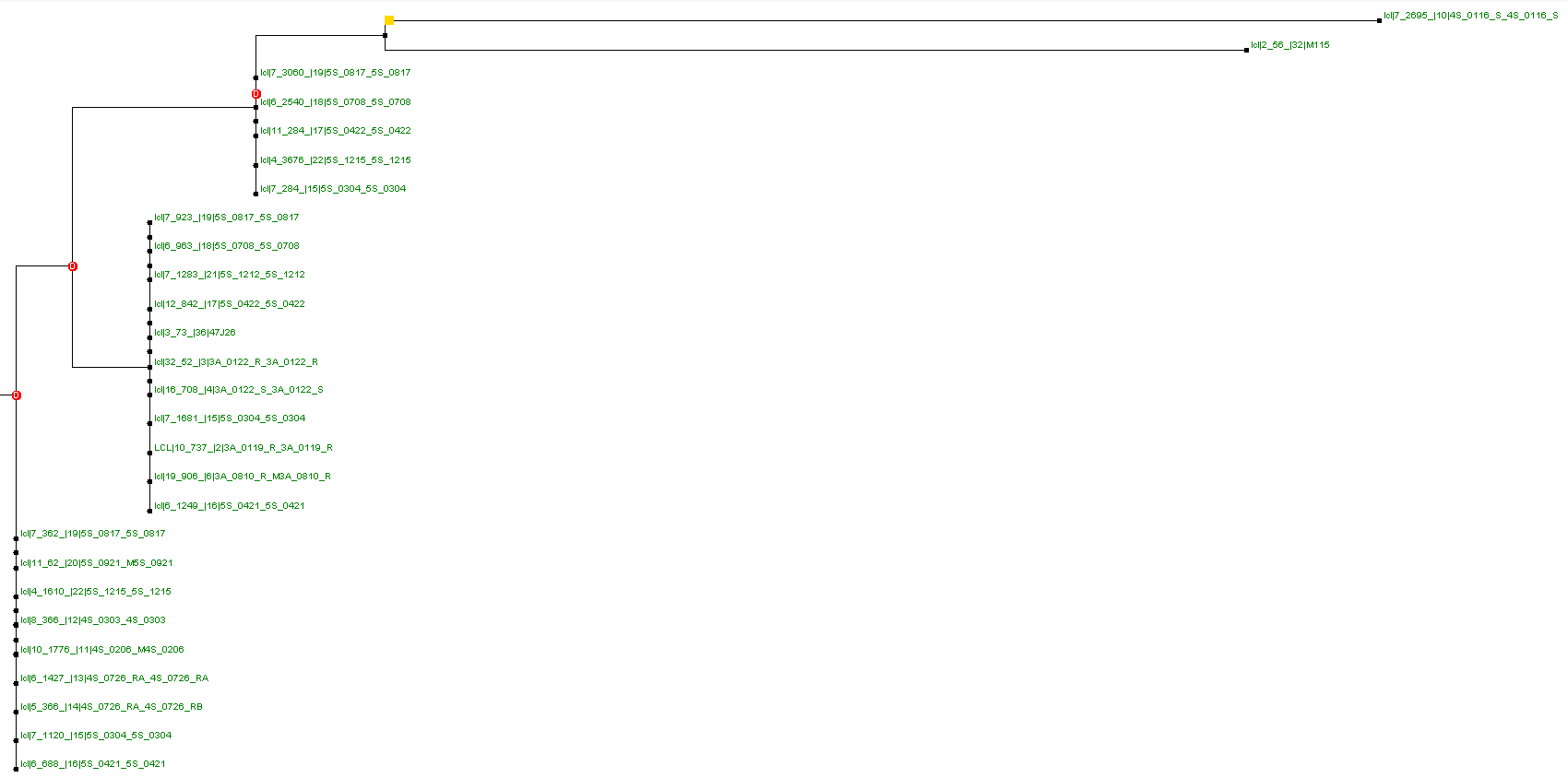

Supplement: Additional file 3 — The reconstructed trees for HGT events. Each tree contains one to six HGT events. The yellow squares represent the HGT event. [file 1745-6150-9-19-S3.zip › Tree_49.png]

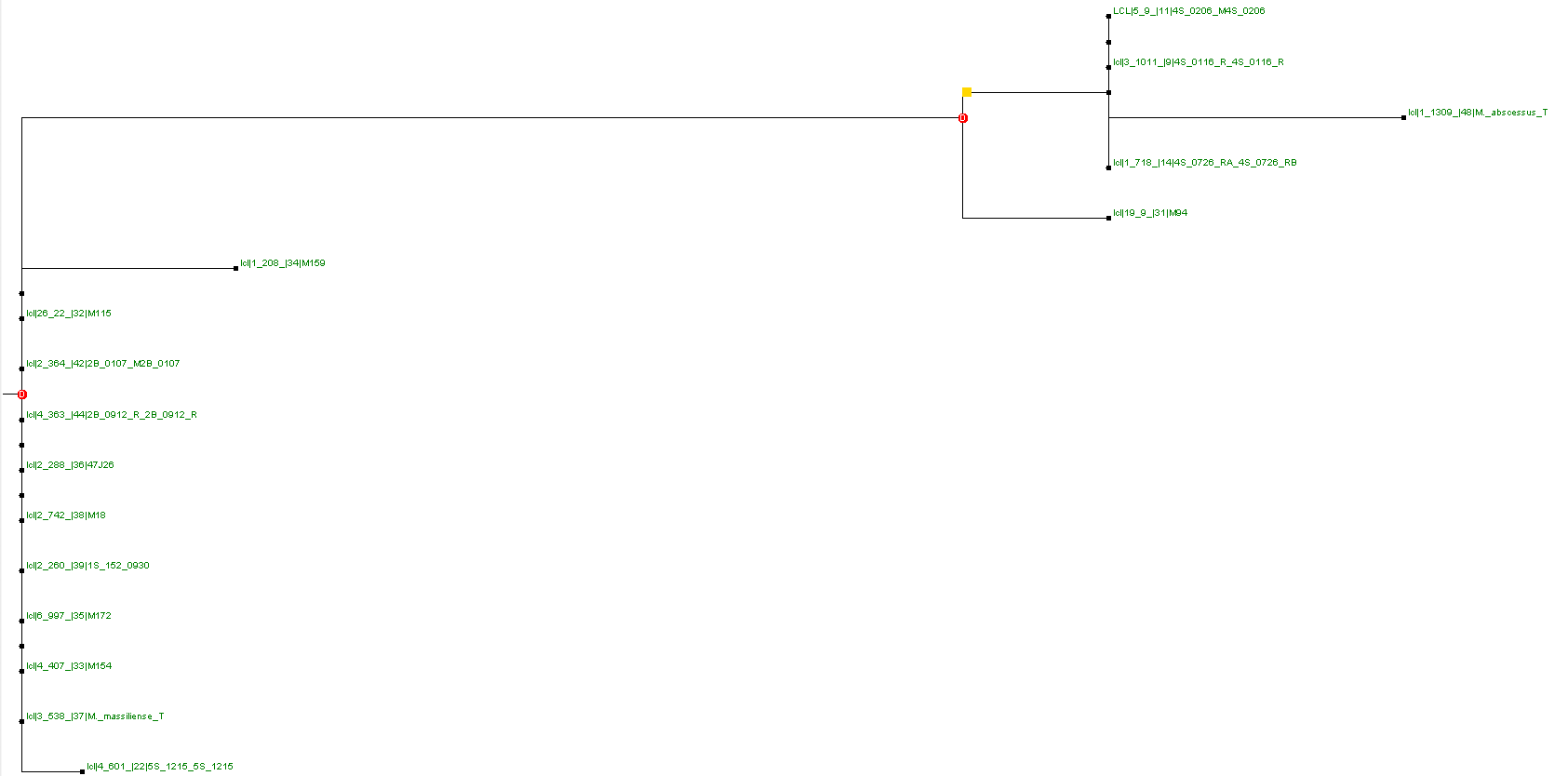

Supplement: Additional file 3 — The reconstructed trees for HGT events. Each tree contains one to six HGT events. The yellow squares represent the HGT event. [file 1745-6150-9-19-S3.zip › Tree_5.png]

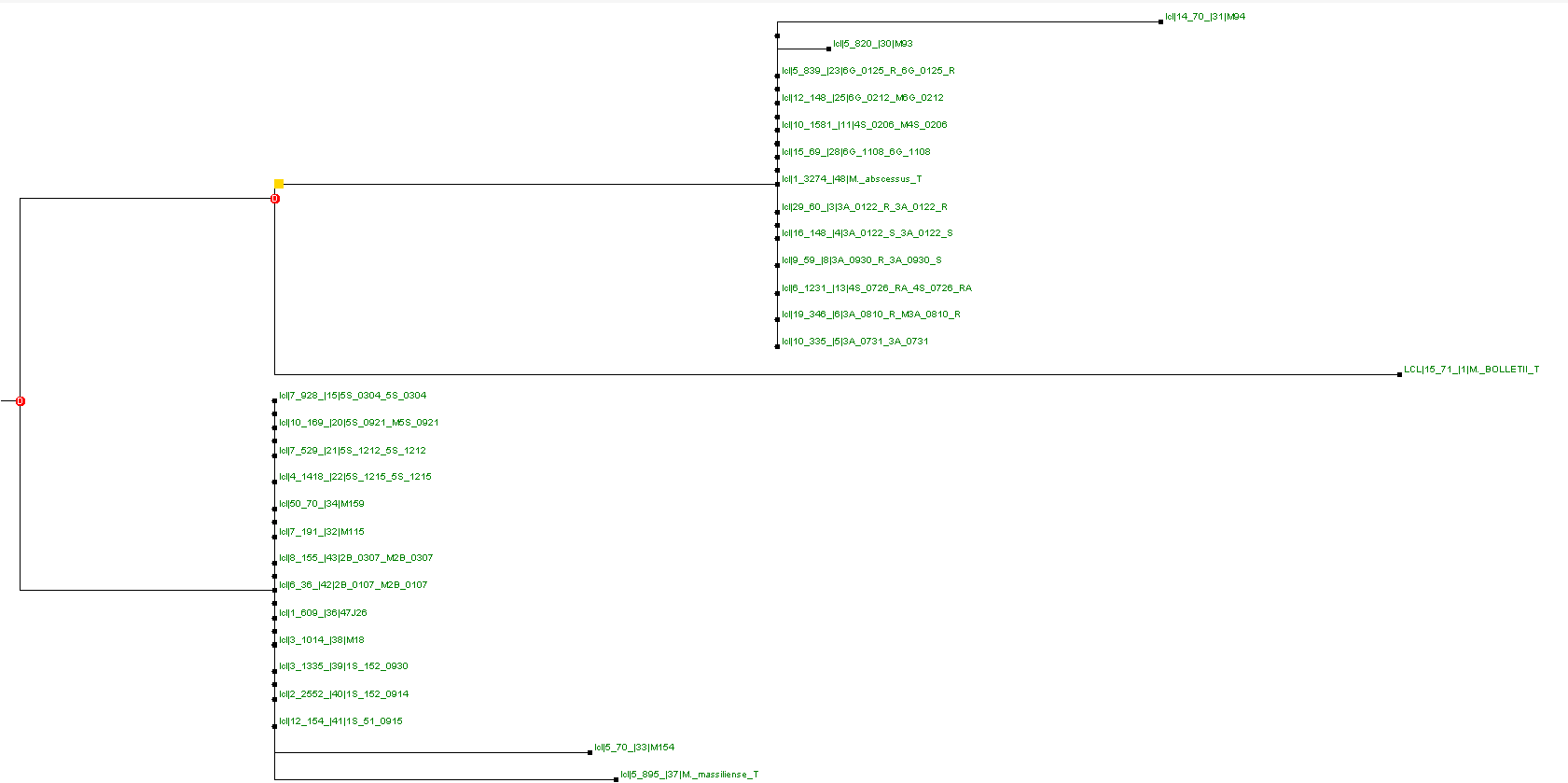

Supplement: Additional file 3 — The reconstructed trees for HGT events. Each tree contains one to six HGT events. The yellow squares represent the HGT event. [file 1745-6150-9-19-S3.zip › Tree_50.png]

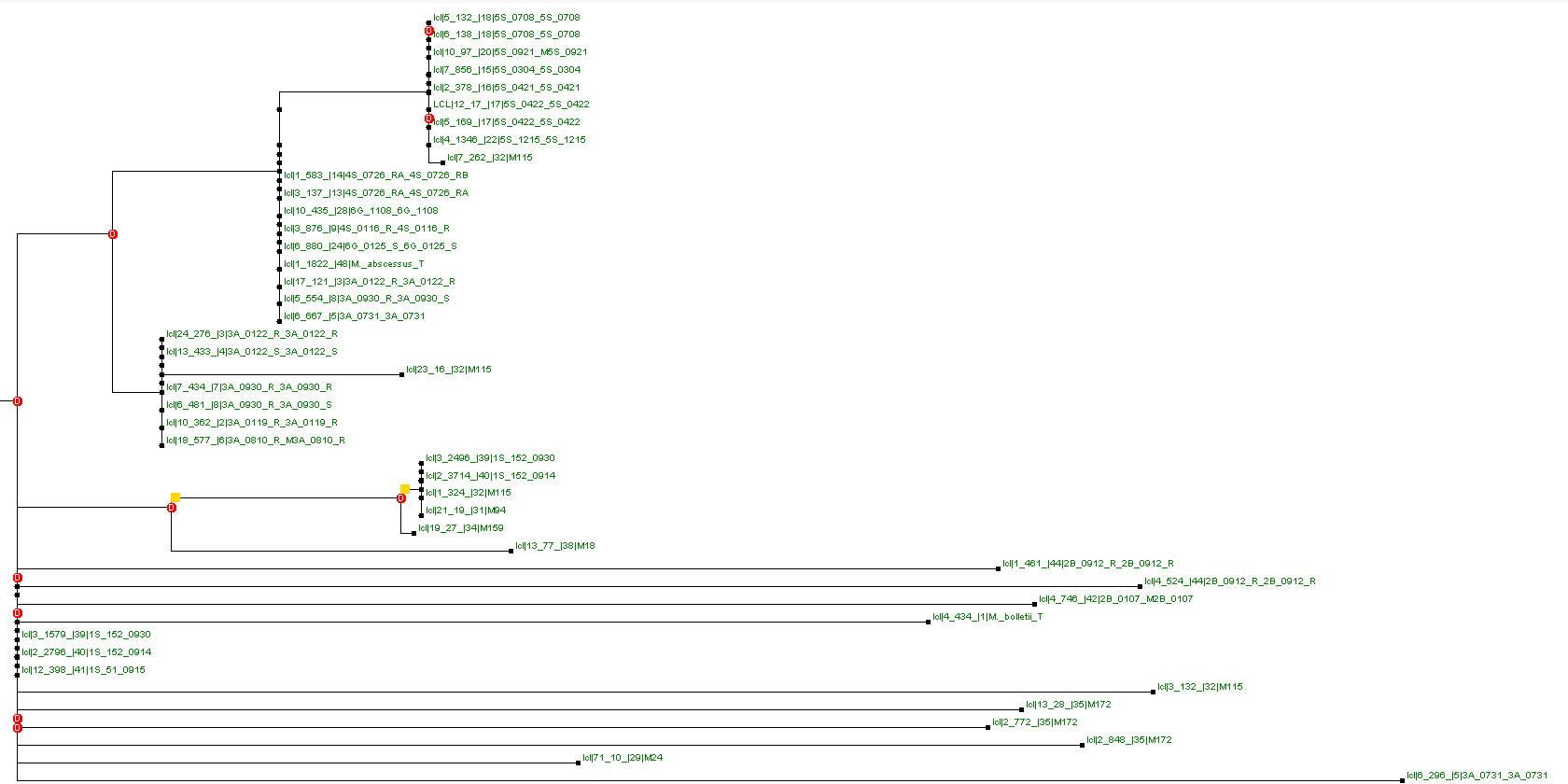

Supplement: Additional file 3 — The reconstructed trees for HGT events. Each tree contains one to six HGT events. The yellow squares represent the HGT event. [file 1745-6150-9-19-S3.zip › Tree_51.png]

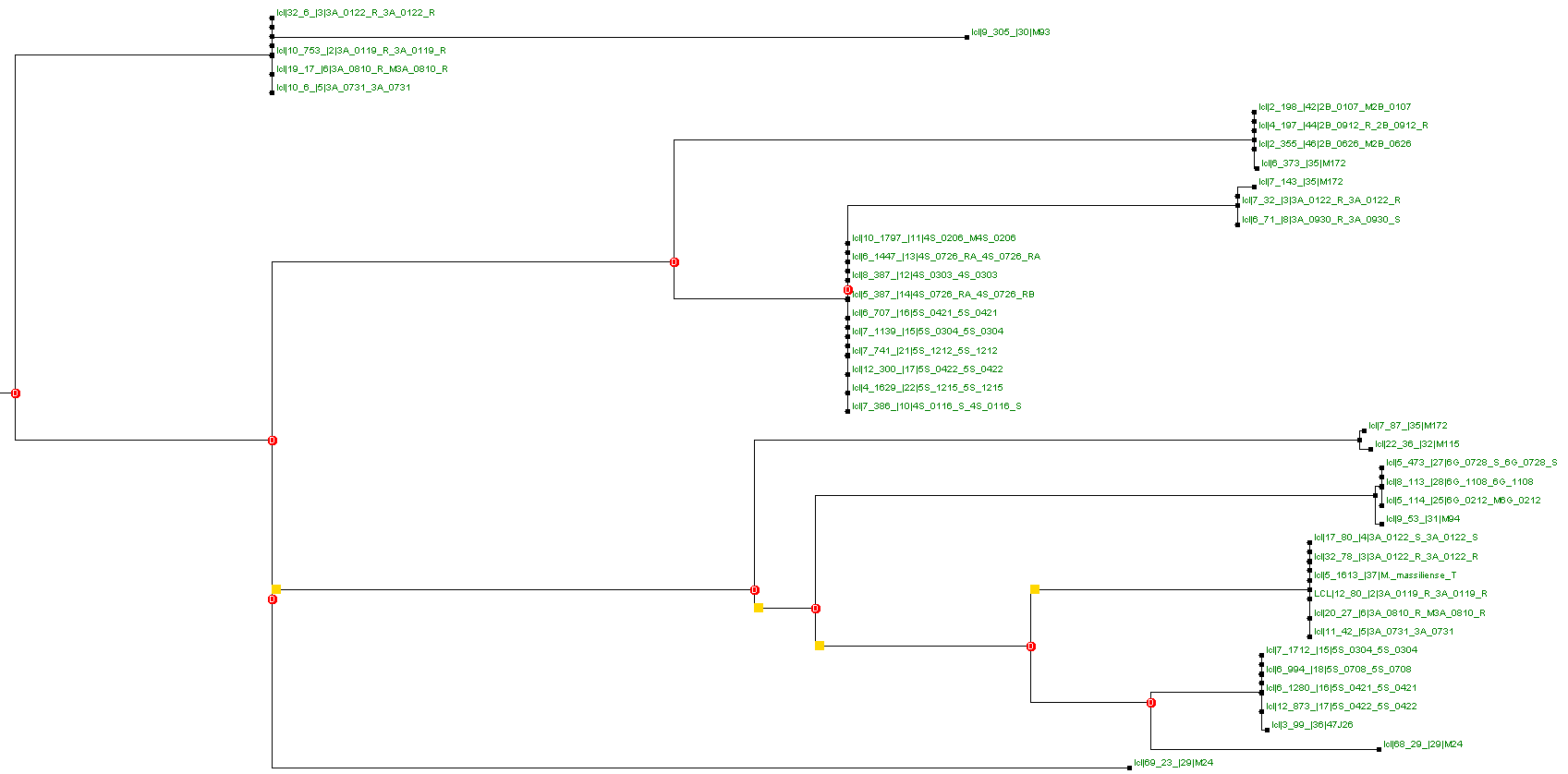

Supplement: Additional file 3 — The reconstructed trees for HGT events. Each tree contains one to six HGT events. The yellow squares represent the HGT event. [file 1745-6150-9-19-S3.zip › Tree_52.png]

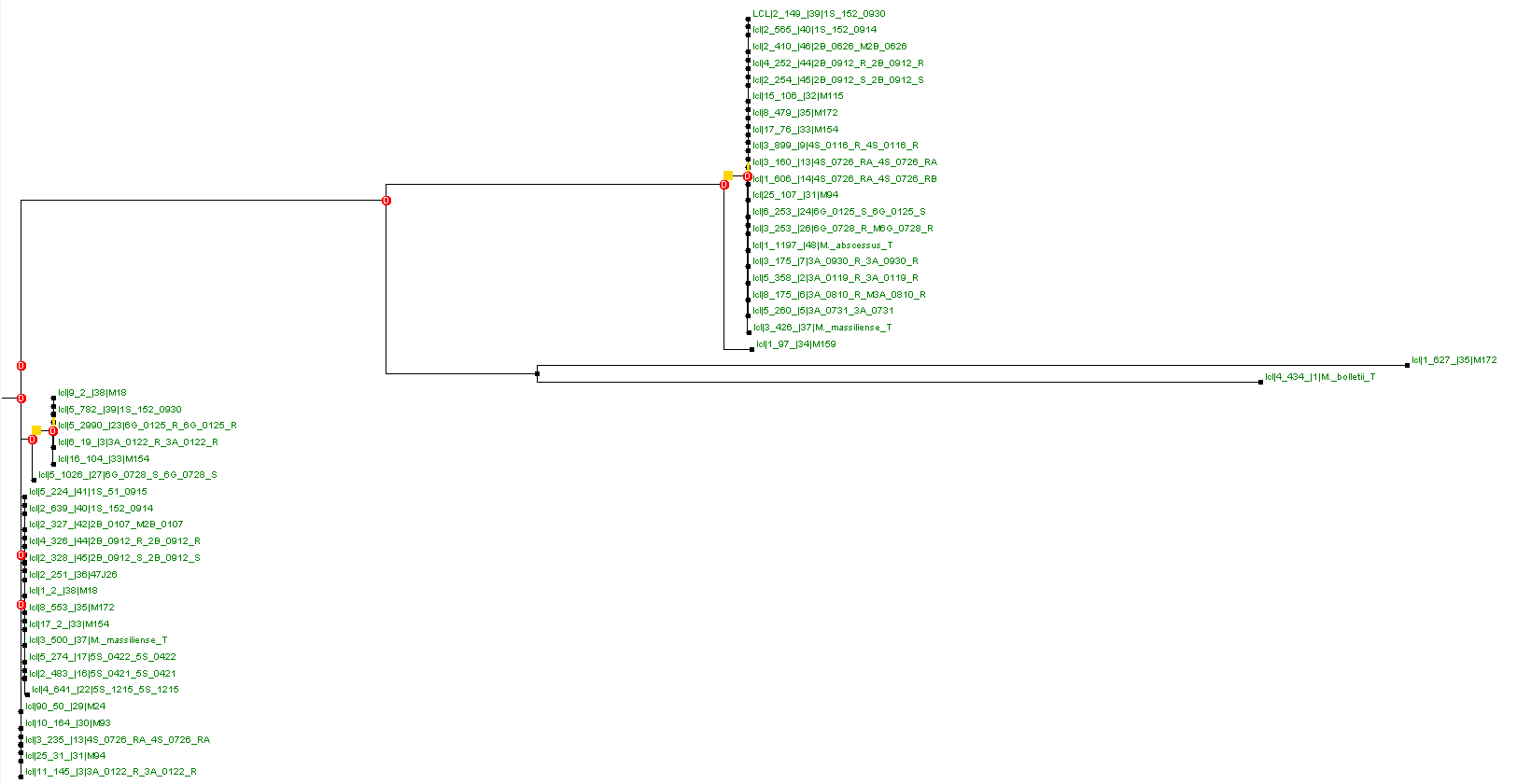

Supplement: Additional file 3 — The reconstructed trees for HGT events. Each tree contains one to six HGT events. The yellow squares represent the HGT event. [file 1745-6150-9-19-S3.zip › Tree_53.png]

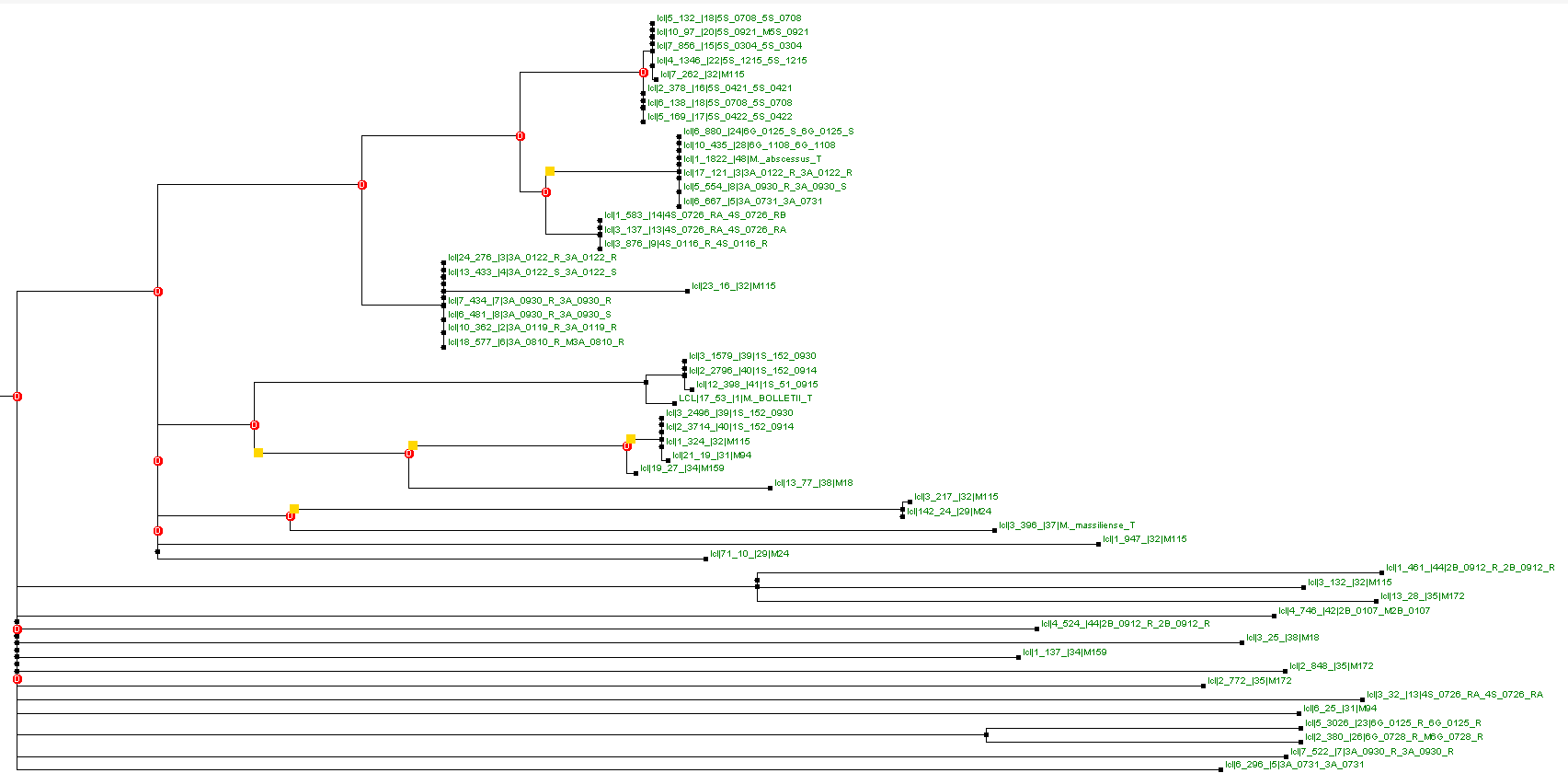

Supplement: Additional file 3 — The reconstructed trees for HGT events. Each tree contains one to six HGT events. The yellow squares represent the HGT event. [file 1745-6150-9-19-S3.zip › Tree_54.png]

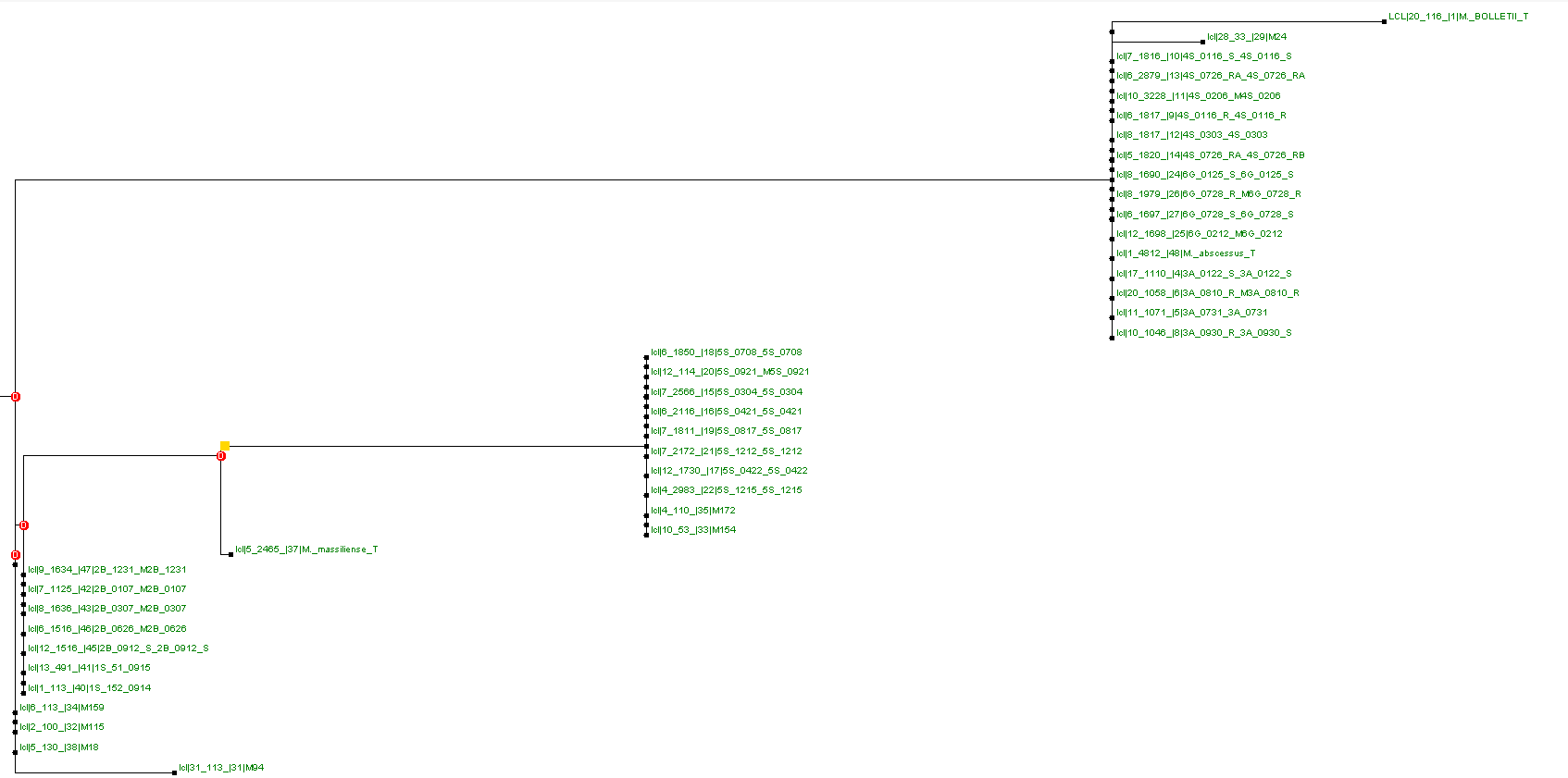

Supplement: Additional file 3 — The reconstructed trees for HGT events. Each tree contains one to six HGT events. The yellow squares represent the HGT event. [file 1745-6150-9-19-S3.zip › Tree_55.png]

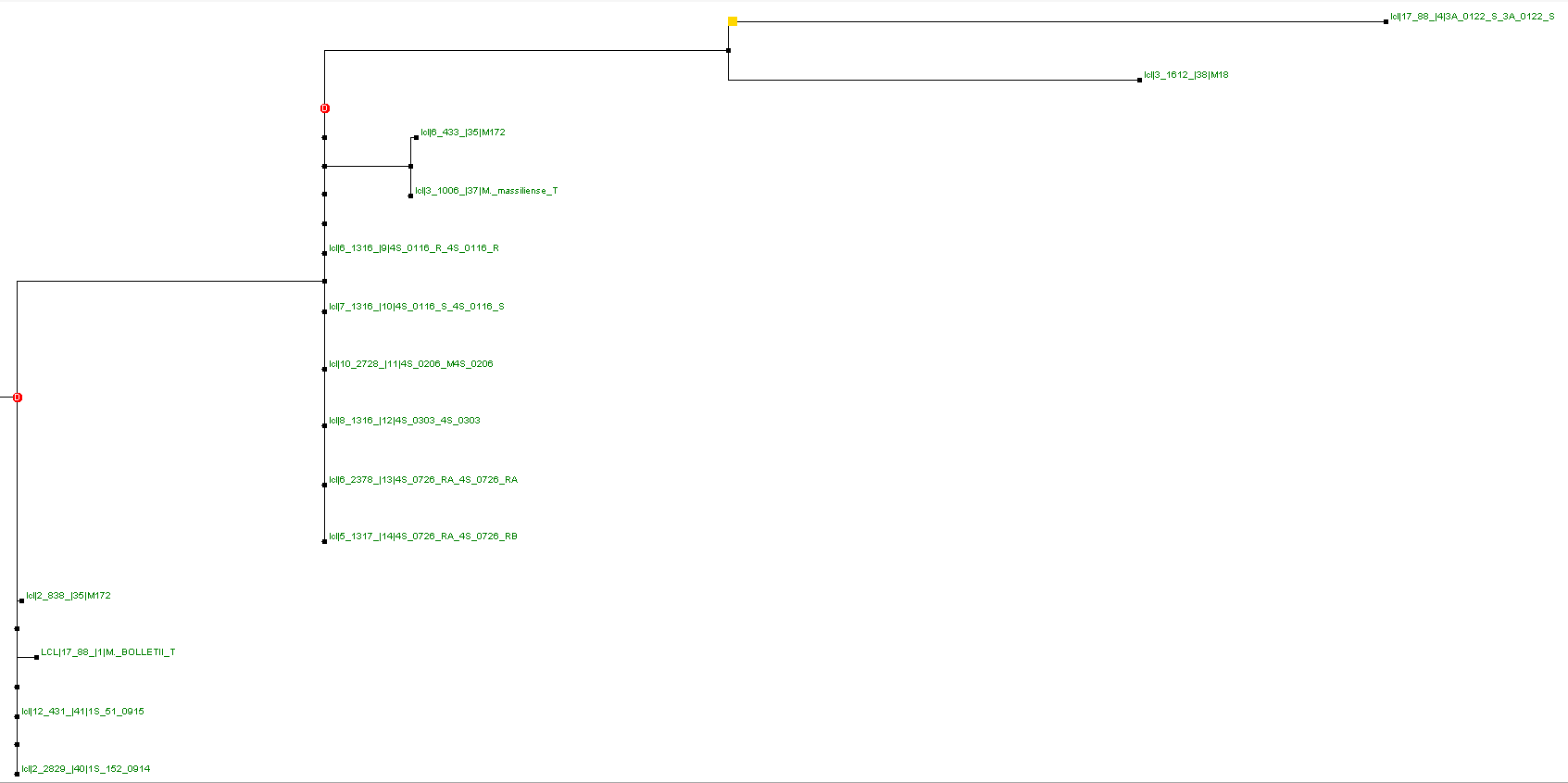

Supplement: Additional file 3 — The reconstructed trees for HGT events. Each tree contains one to six HGT events. The yellow squares represent the HGT event. [file 1745-6150-9-19-S3.zip › Tree_56.png]

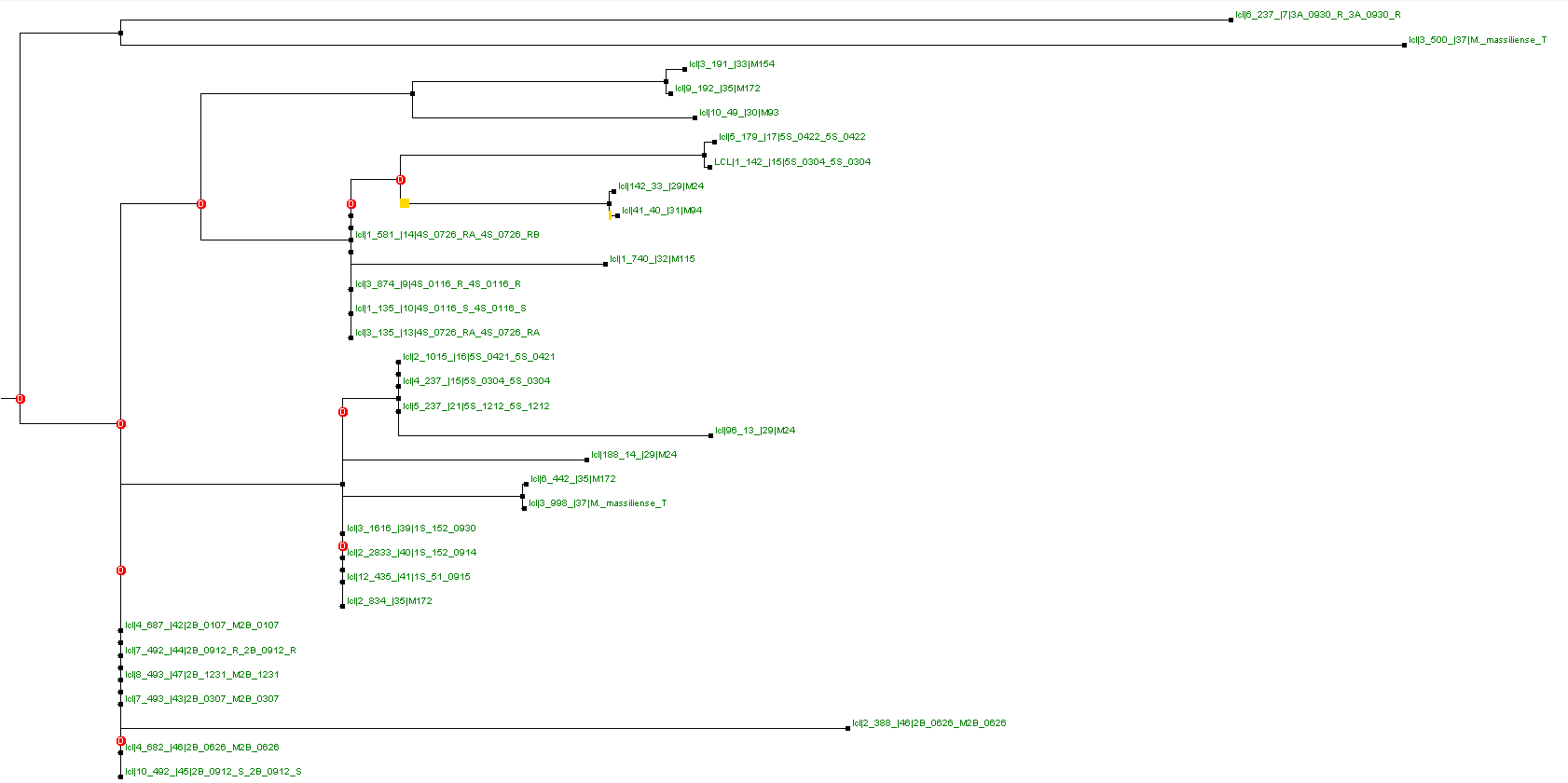

Supplement: Additional file 3 — The reconstructed trees for HGT events. Each tree contains one to six HGT events. The yellow squares represent the HGT event. [file 1745-6150-9-19-S3.zip › Tree_57.png]

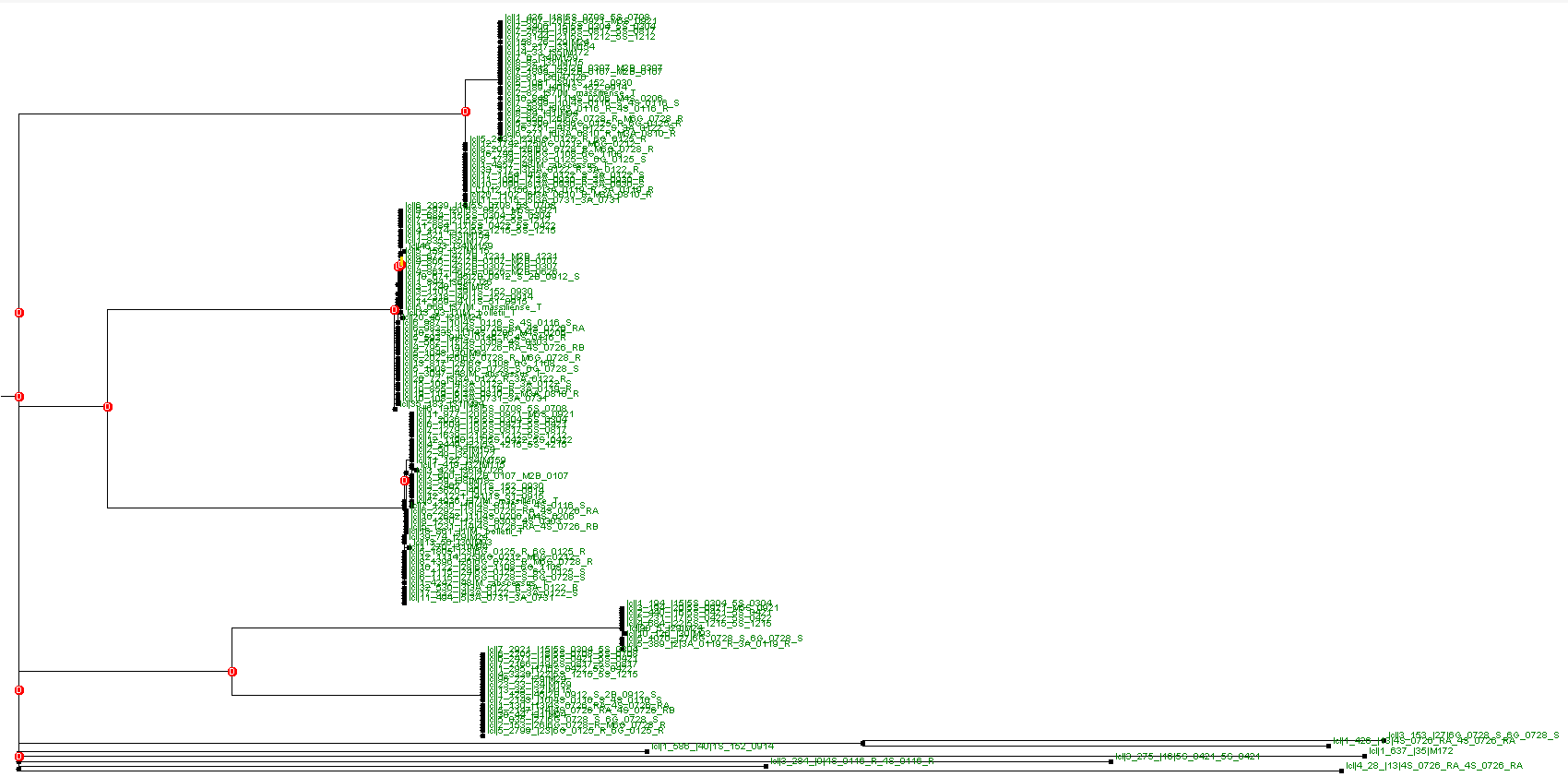

Supplement: Additional file 3 — The reconstructed trees for HGT events. Each tree contains one to six HGT events. The yellow squares represent the HGT event. [file 1745-6150-9-19-S3.zip › Tree_58.png]

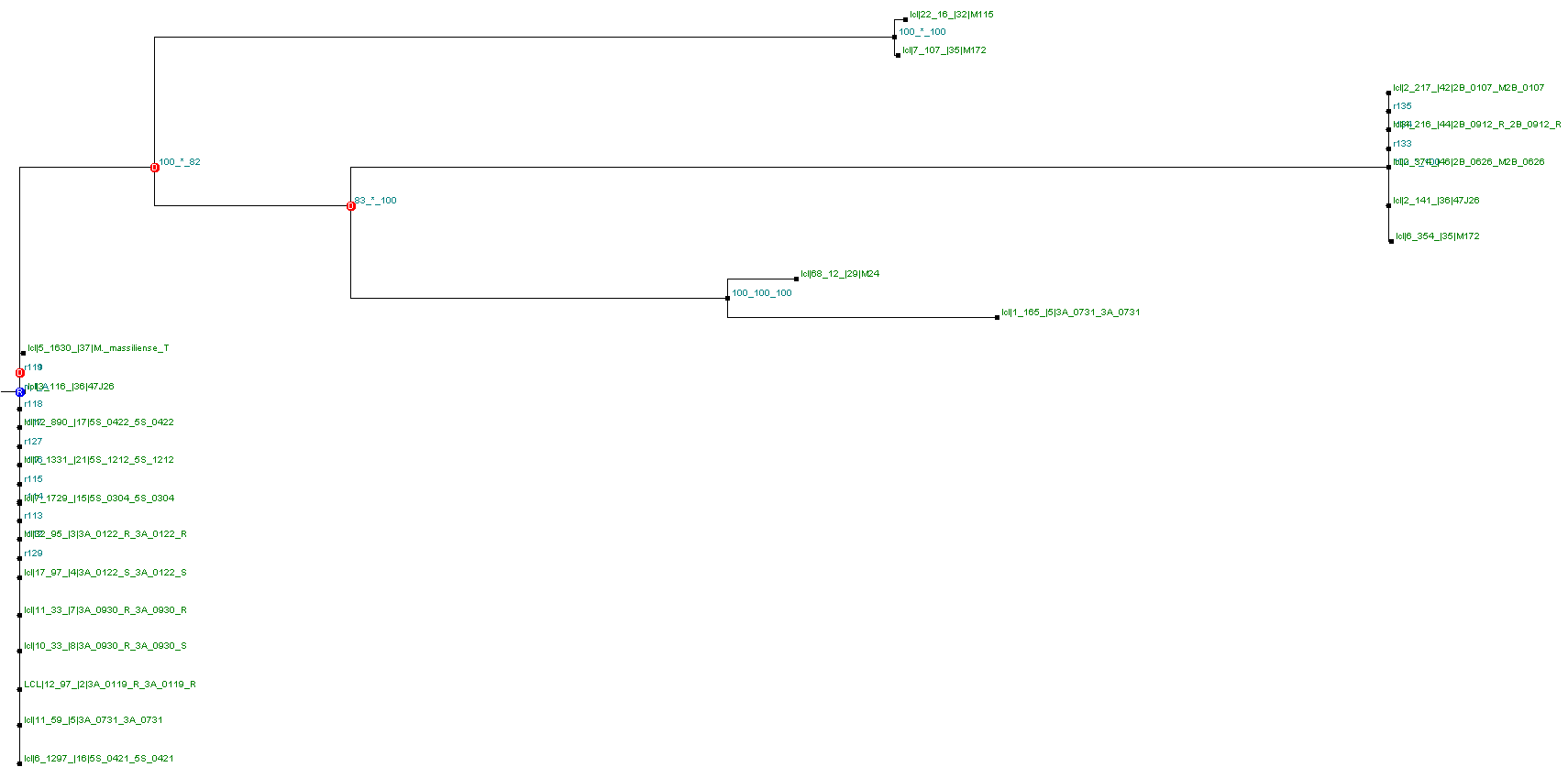

Supplement: Additional file 3 — The reconstructed trees for HGT events. Each tree contains one to six HGT events. The yellow squares represent the HGT event. [file 1745-6150-9-19-S3.zip › Tree_59.png]

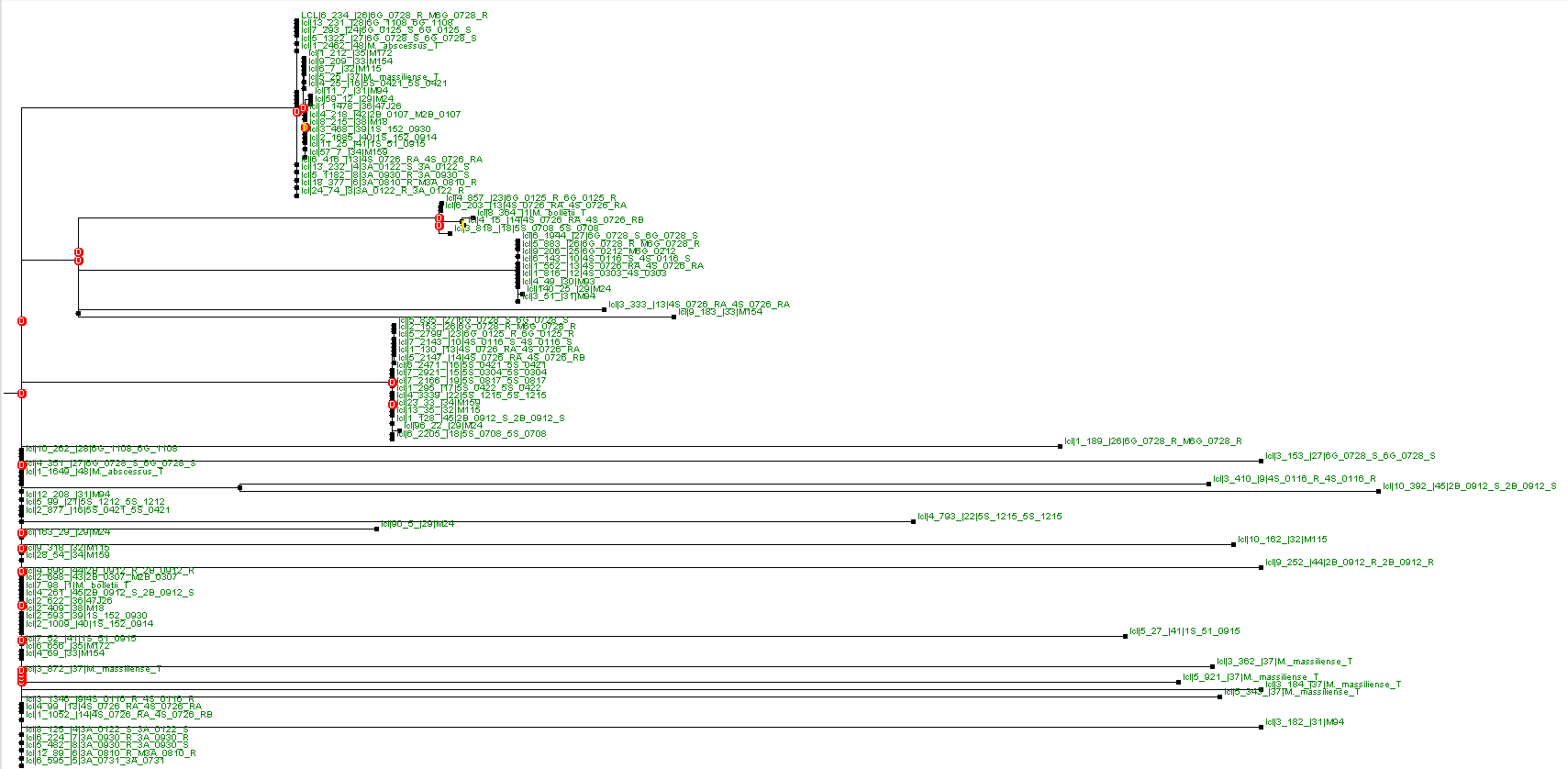

Supplement: Additional file 3 — The reconstructed trees for HGT events. Each tree contains one to six HGT events. The yellow squares represent the HGT event. [file 1745-6150-9-19-S3.zip › Tree_6.png]

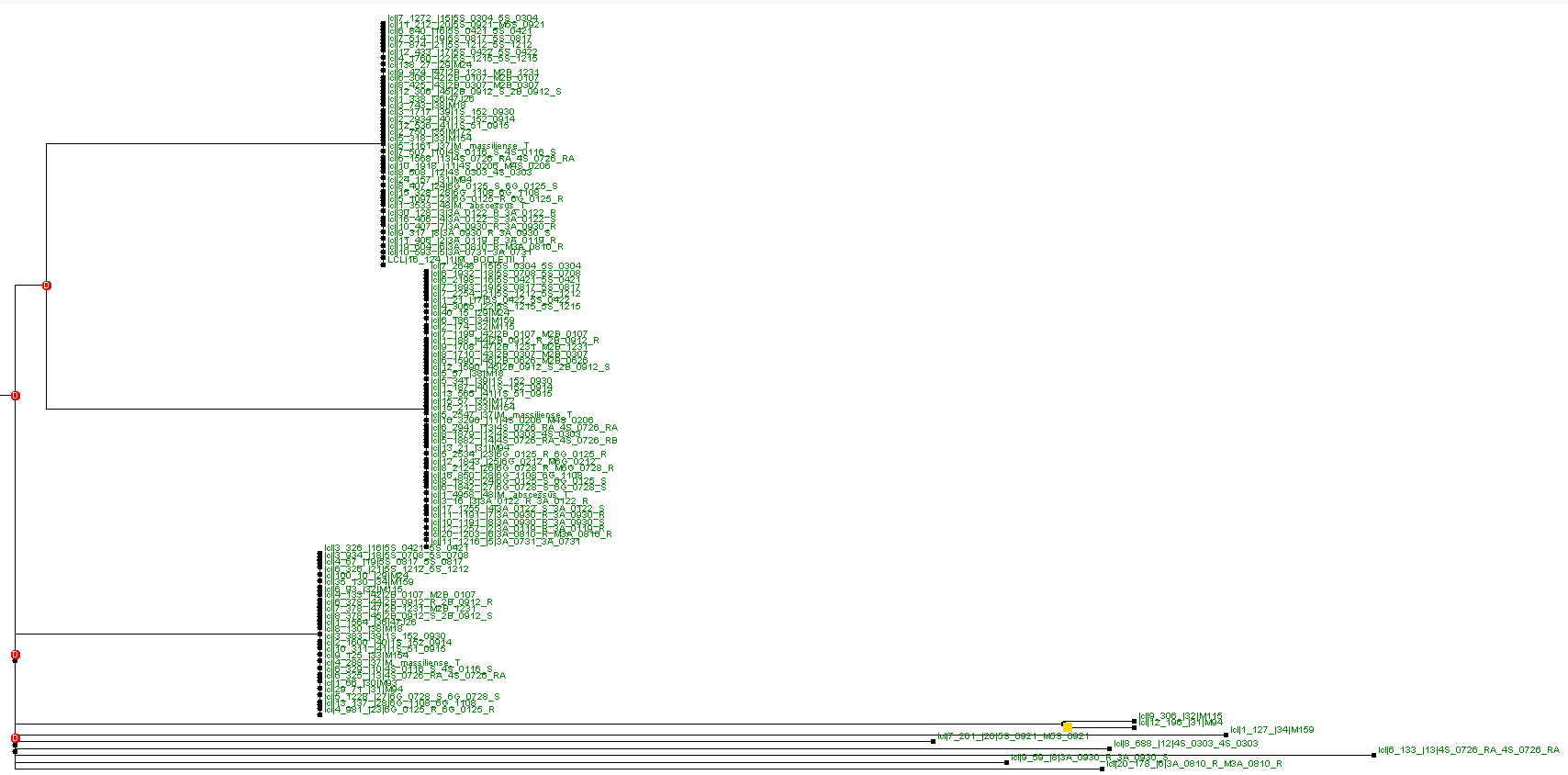

Supplement: Additional file 3 — The reconstructed trees for HGT events. Each tree contains one to six HGT events. The yellow squares represent the HGT event. [file 1745-6150-9-19-S3.zip › Tree_60.png]

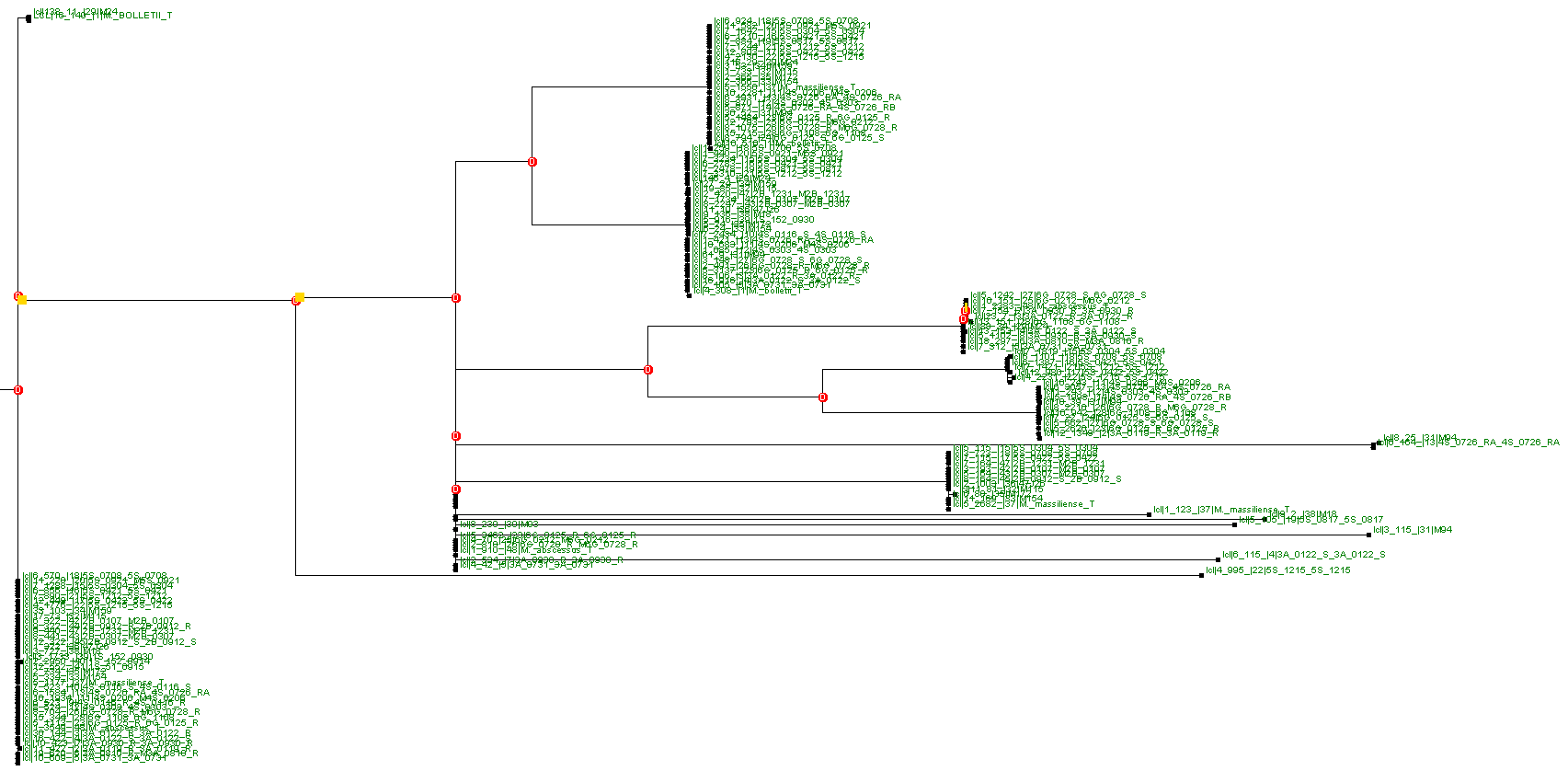

Supplement: Additional file 3 — The reconstructed trees for HGT events. Each tree contains one to six HGT events. The yellow squares represent the HGT event. [file 1745-6150-9-19-S3.zip › Tree_61.png]

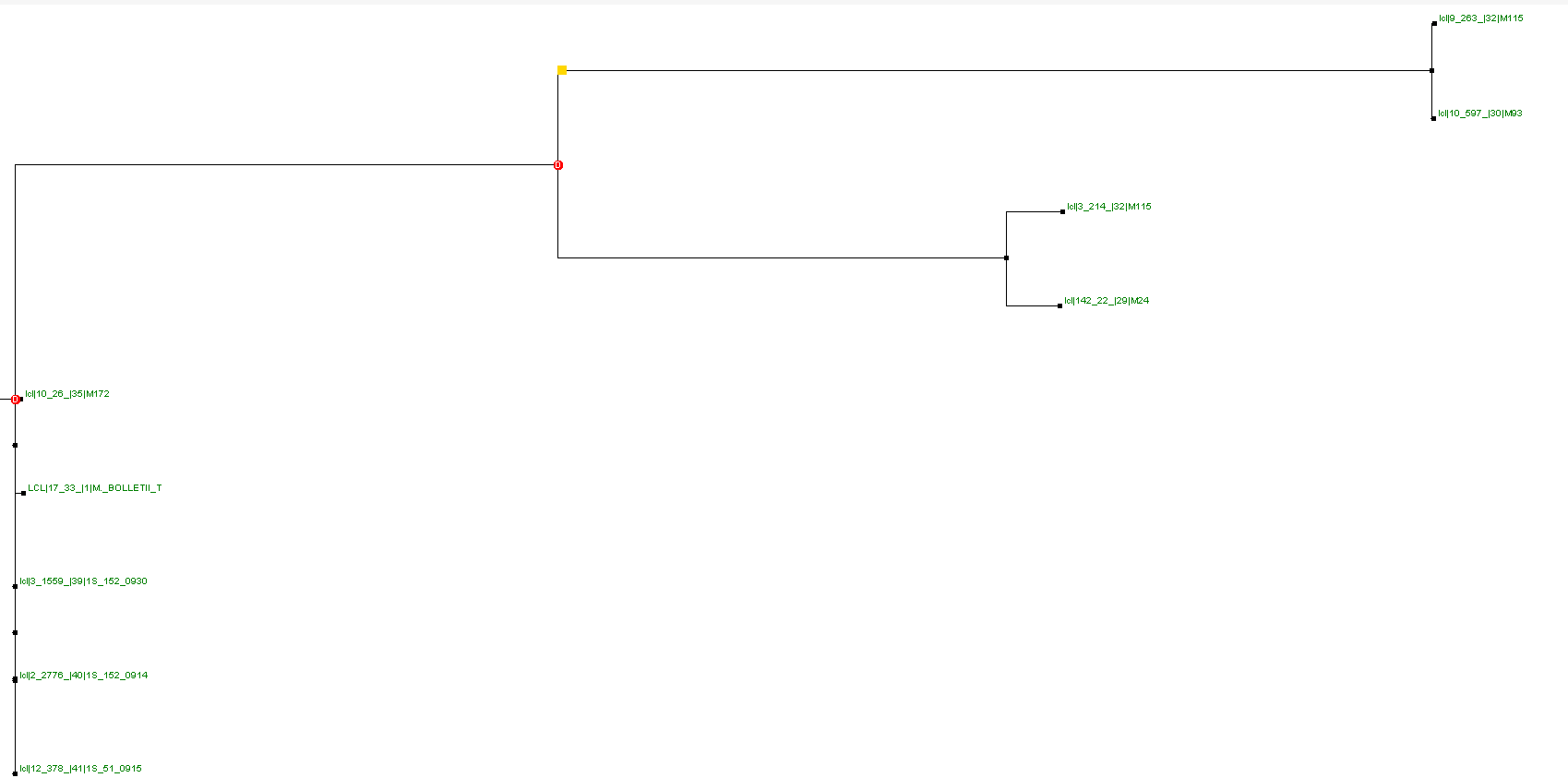

Supplement: Additional file 3 — The reconstructed trees for HGT events. Each tree contains one to six HGT events. The yellow squares represent the HGT event. [file 1745-6150-9-19-S3.zip › Tree_62.png]

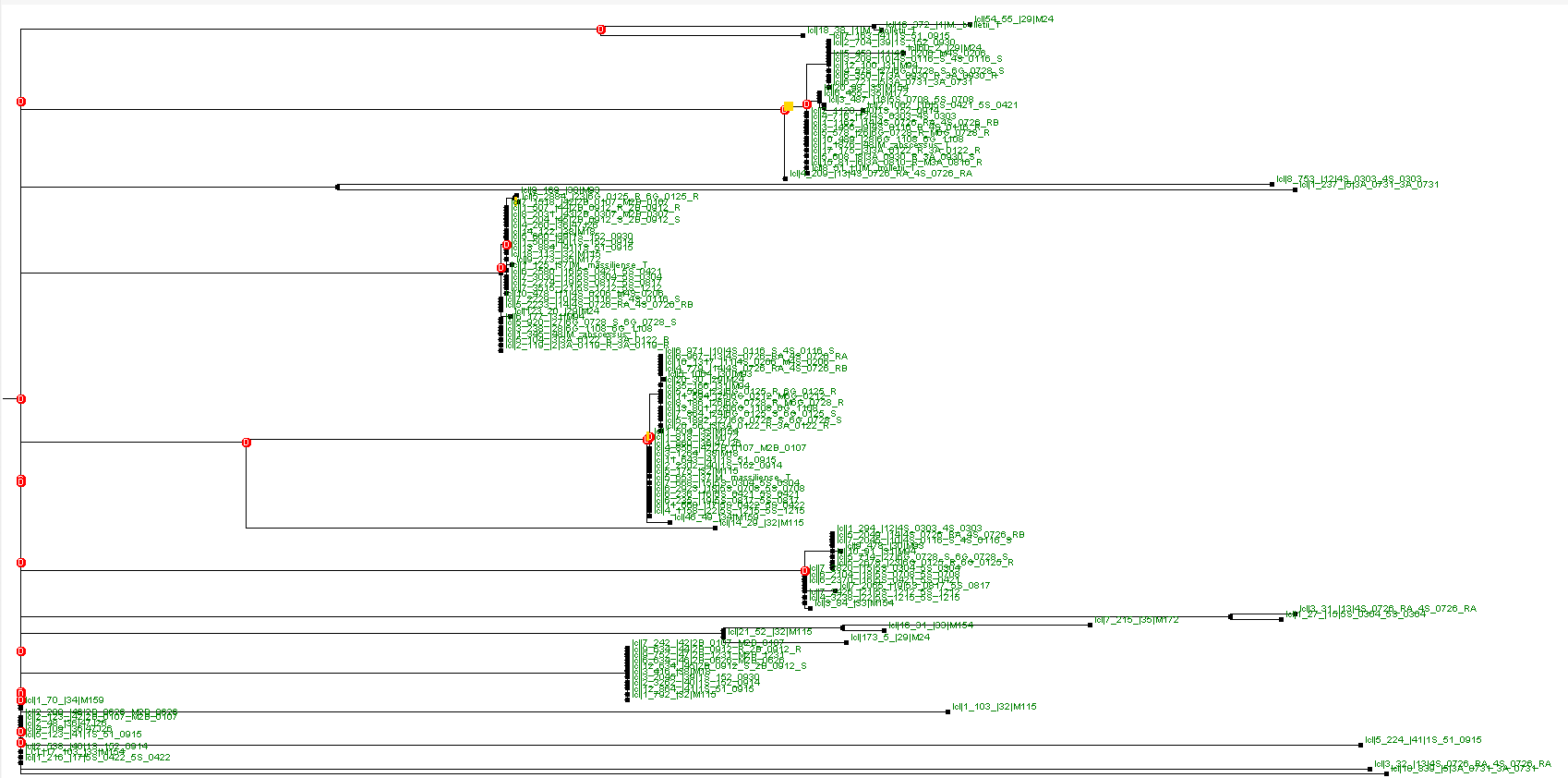

Supplement: Additional file 3 — The reconstructed trees for HGT events. Each tree contains one to six HGT events. The yellow squares represent the HGT event. [file 1745-6150-9-19-S3.zip › Tree_63.png]

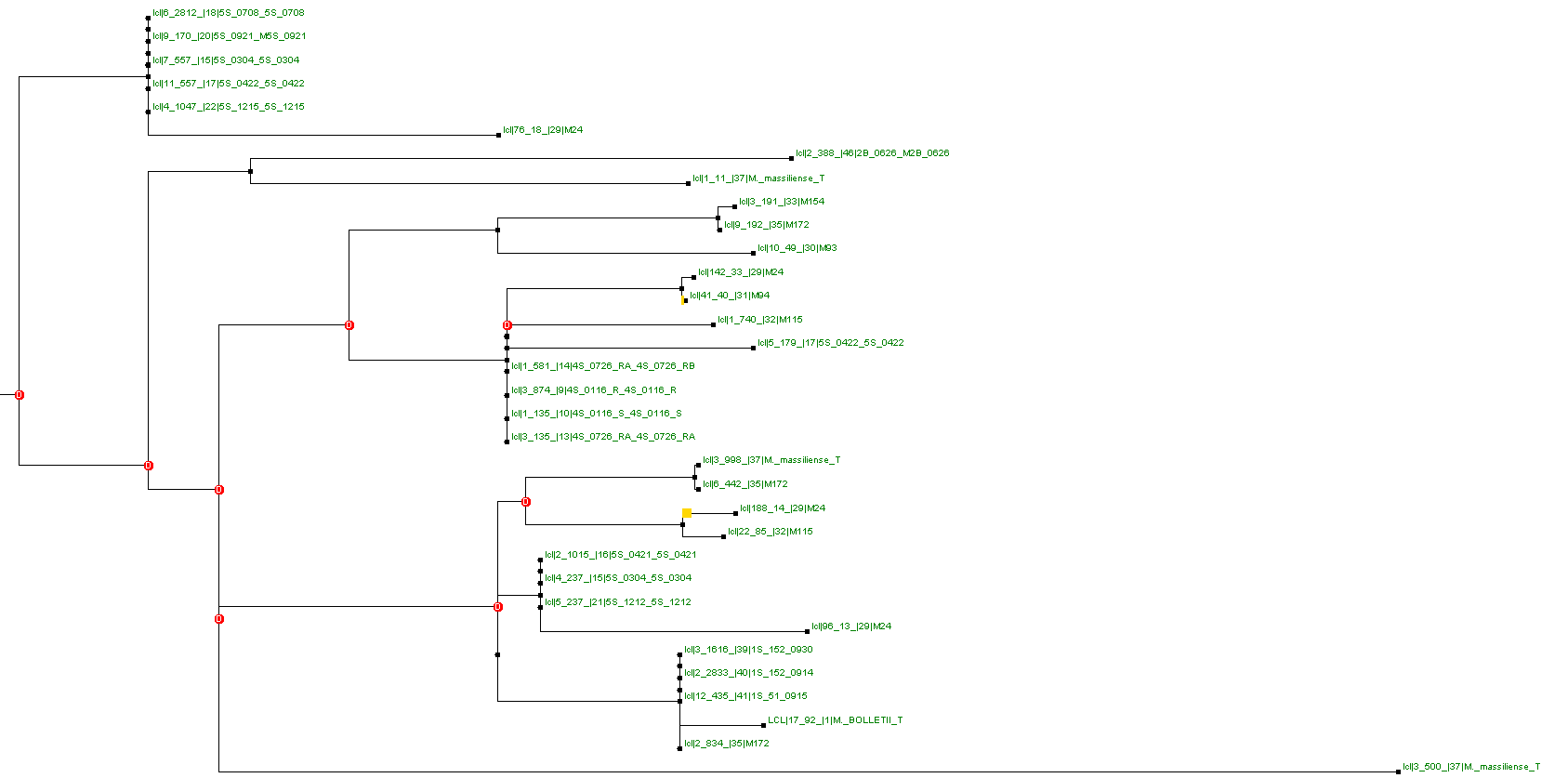

Supplement: Additional file 3 — The reconstructed trees for HGT events. Each tree contains one to six HGT events. The yellow squares represent the HGT event. [file 1745-6150-9-19-S3.zip › Tree_64.png]

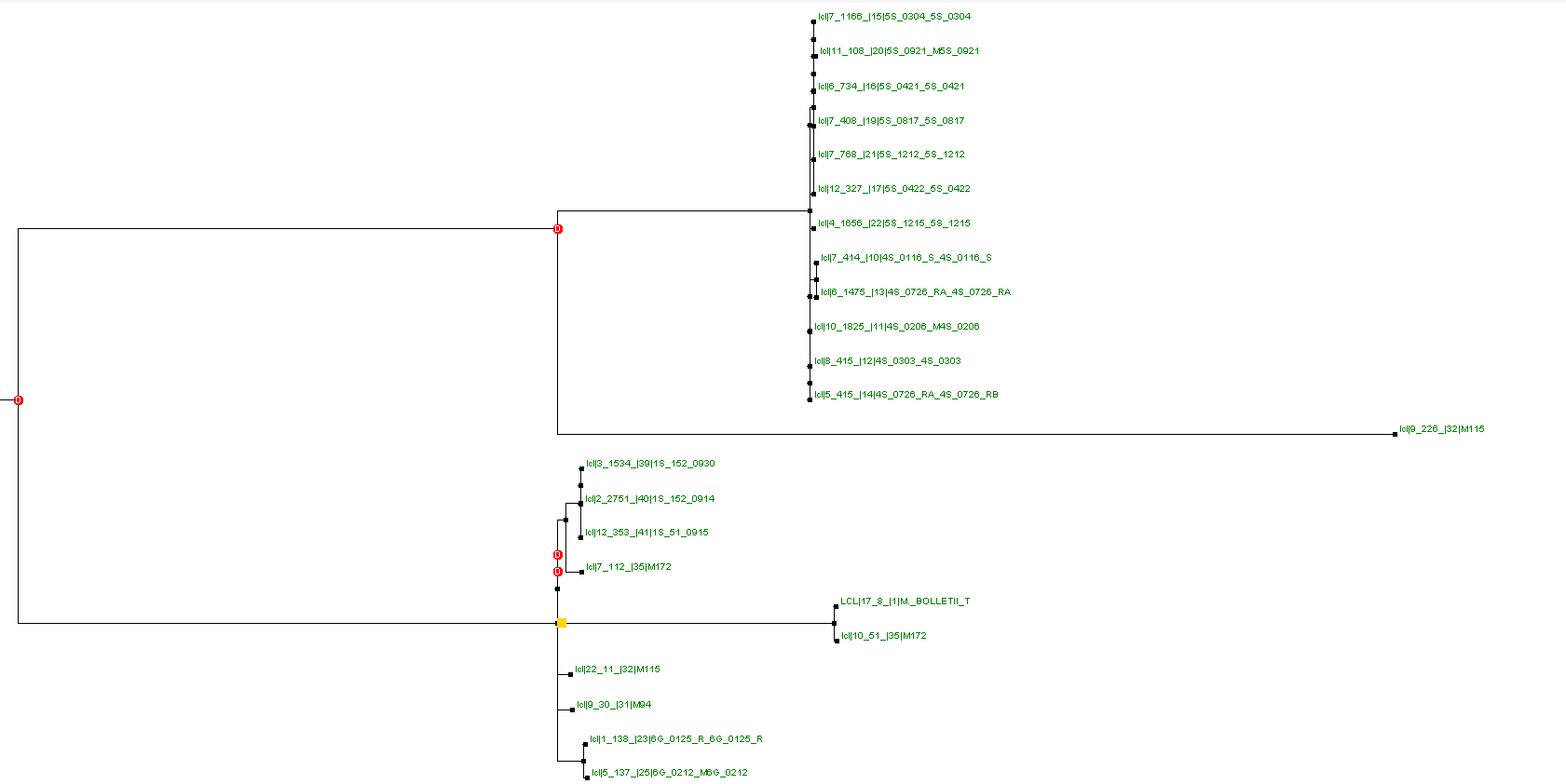

Supplement: Additional file 3 — The reconstructed trees for HGT events. Each tree contains one to six HGT events. The yellow squares represent the HGT event. [file 1745-6150-9-19-S3.zip › Tree_65.png]

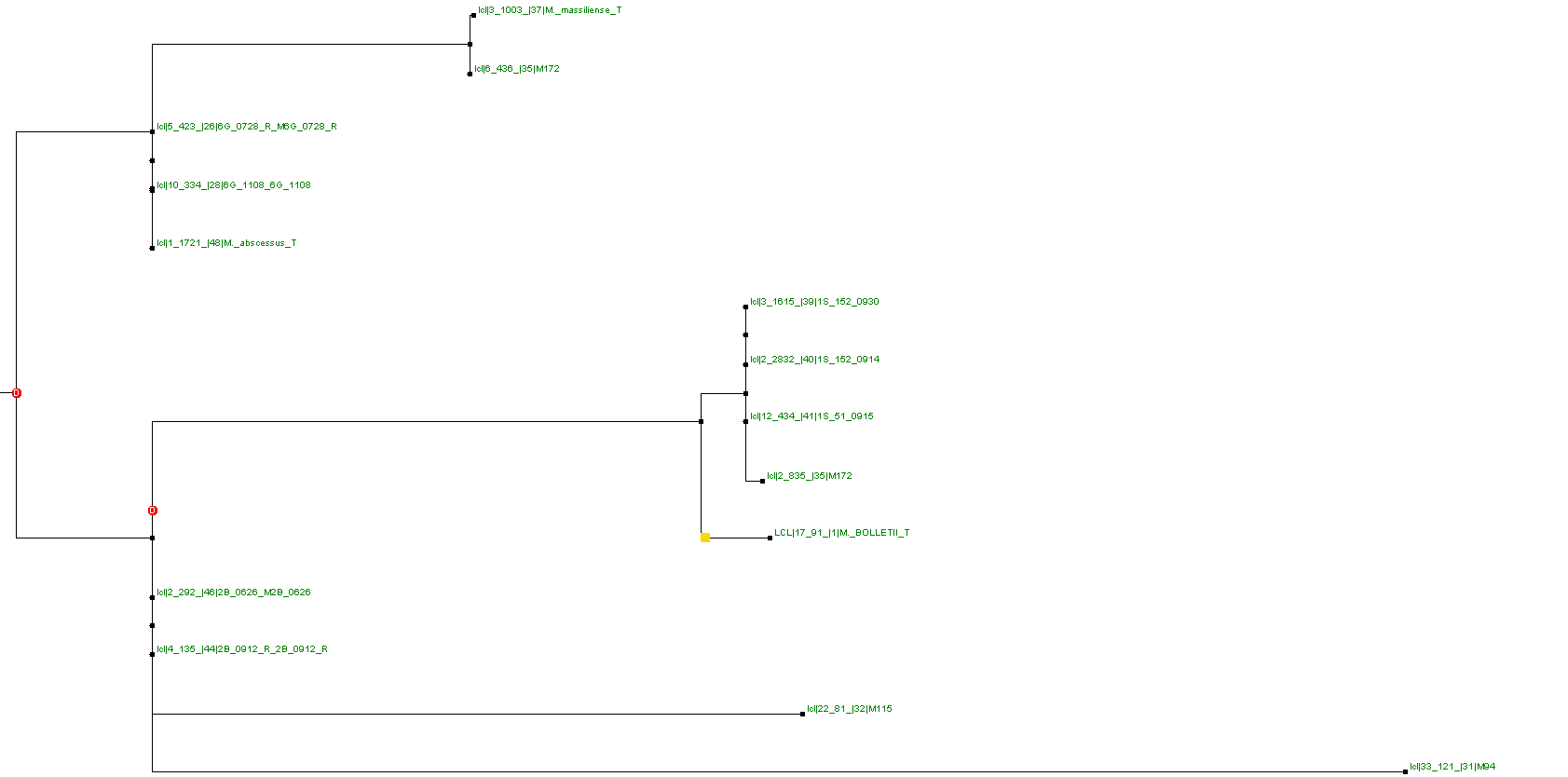

Supplement: Additional file 3 — The reconstructed trees for HGT events. Each tree contains one to six HGT events. The yellow squares represent the HGT event. [file 1745-6150-9-19-S3.zip › Tree_66.png]

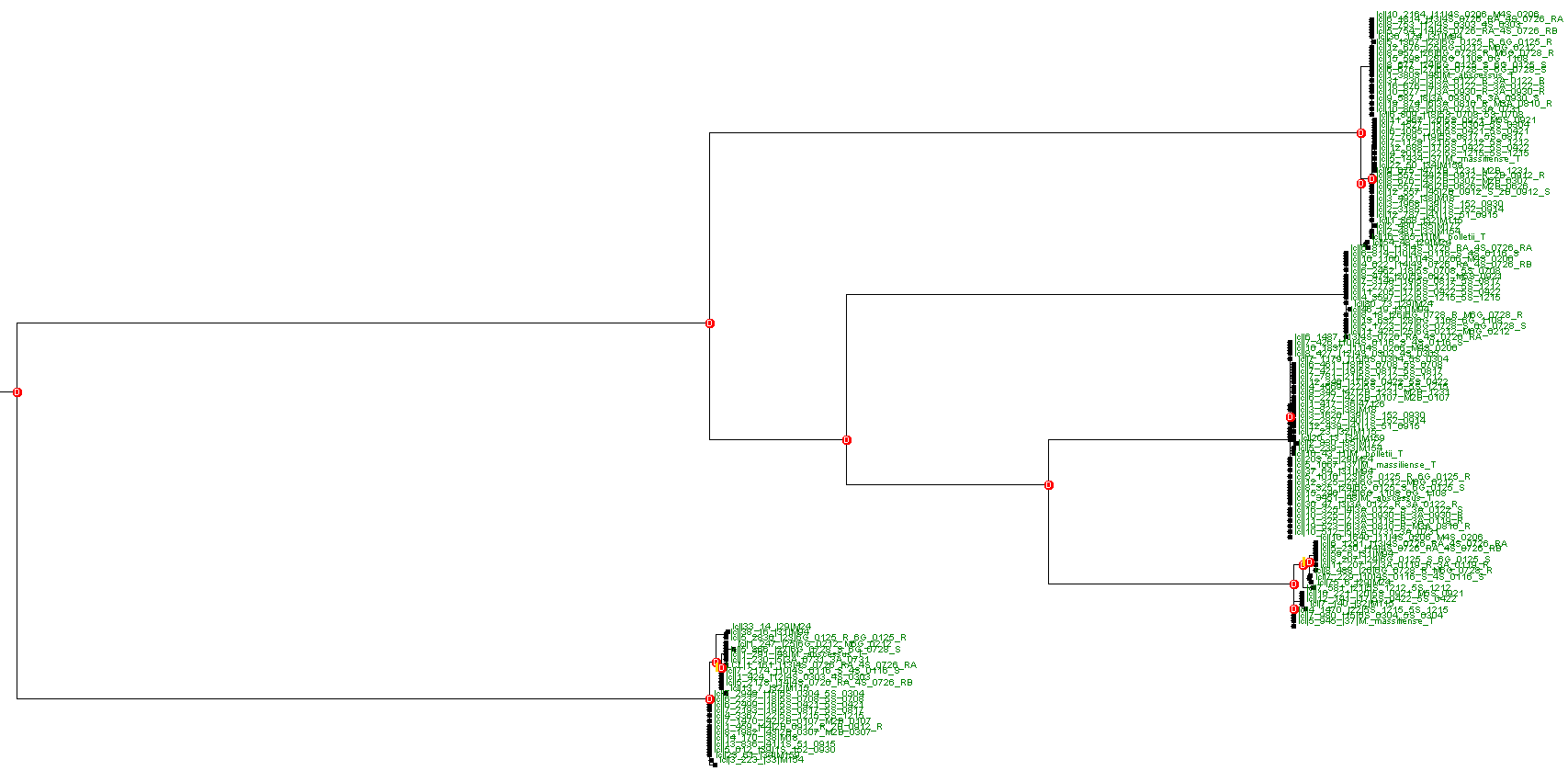

Supplement: Additional file 3 — The reconstructed trees for HGT events. Each tree contains one to six HGT events. The yellow squares represent the HGT event. [file 1745-6150-9-19-S3.zip › Tree_67.png]

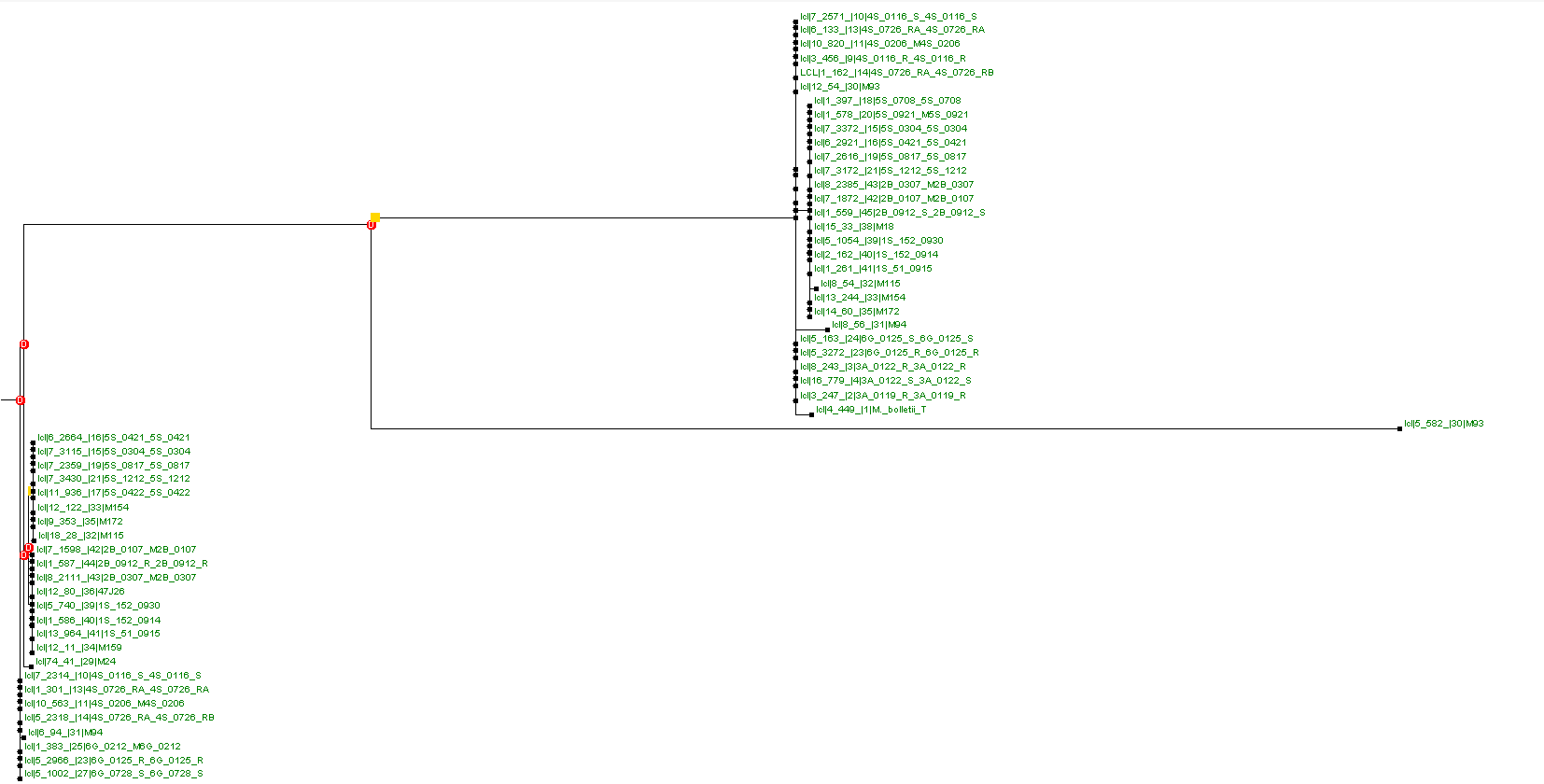

Supplement: Additional file 3 — The reconstructed trees for HGT events. Each tree contains one to six HGT events. The yellow squares represent the HGT event. [file 1745-6150-9-19-S3.zip › Tree_68.png]

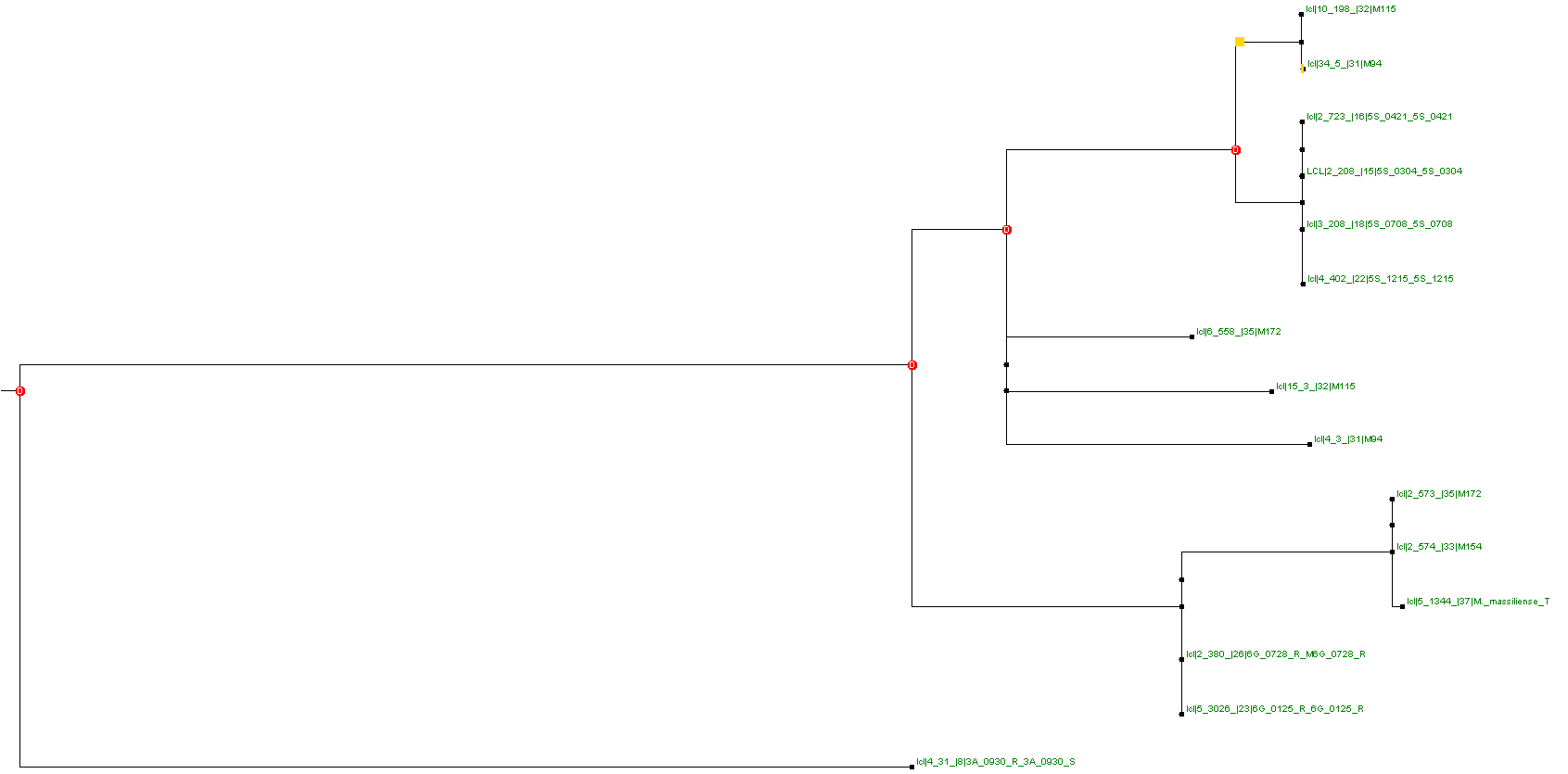

Supplement: Additional file 3 — The reconstructed trees for HGT events. Each tree contains one to six HGT events. The yellow squares represent the HGT event. [file 1745-6150-9-19-S3.zip › Tree_69.png]

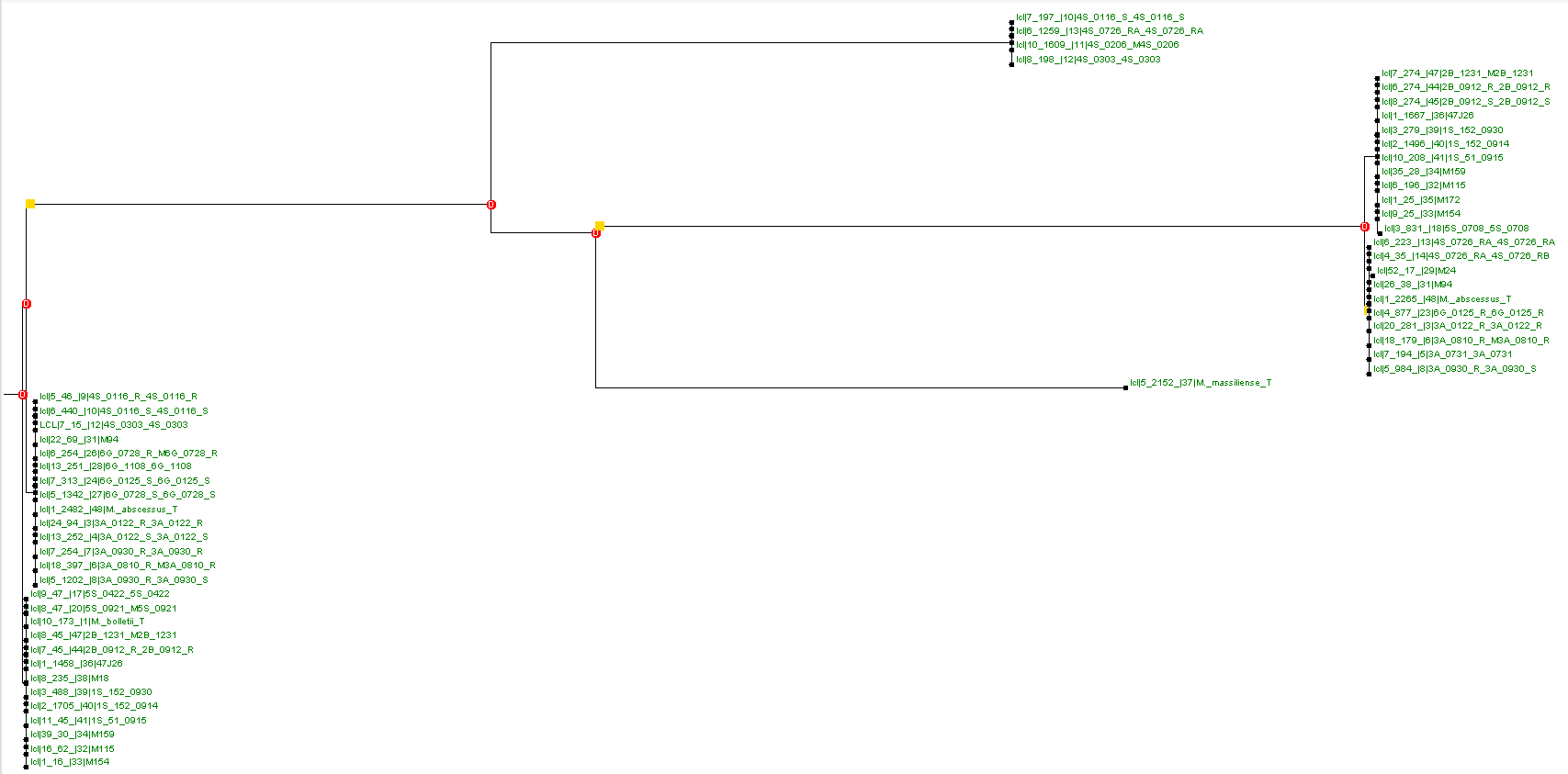

Supplement: Additional file 3 — The reconstructed trees for HGT events. Each tree contains one to six HGT events. The yellow squares represent the HGT event. [file 1745-6150-9-19-S3.zip › Tree_7.png]

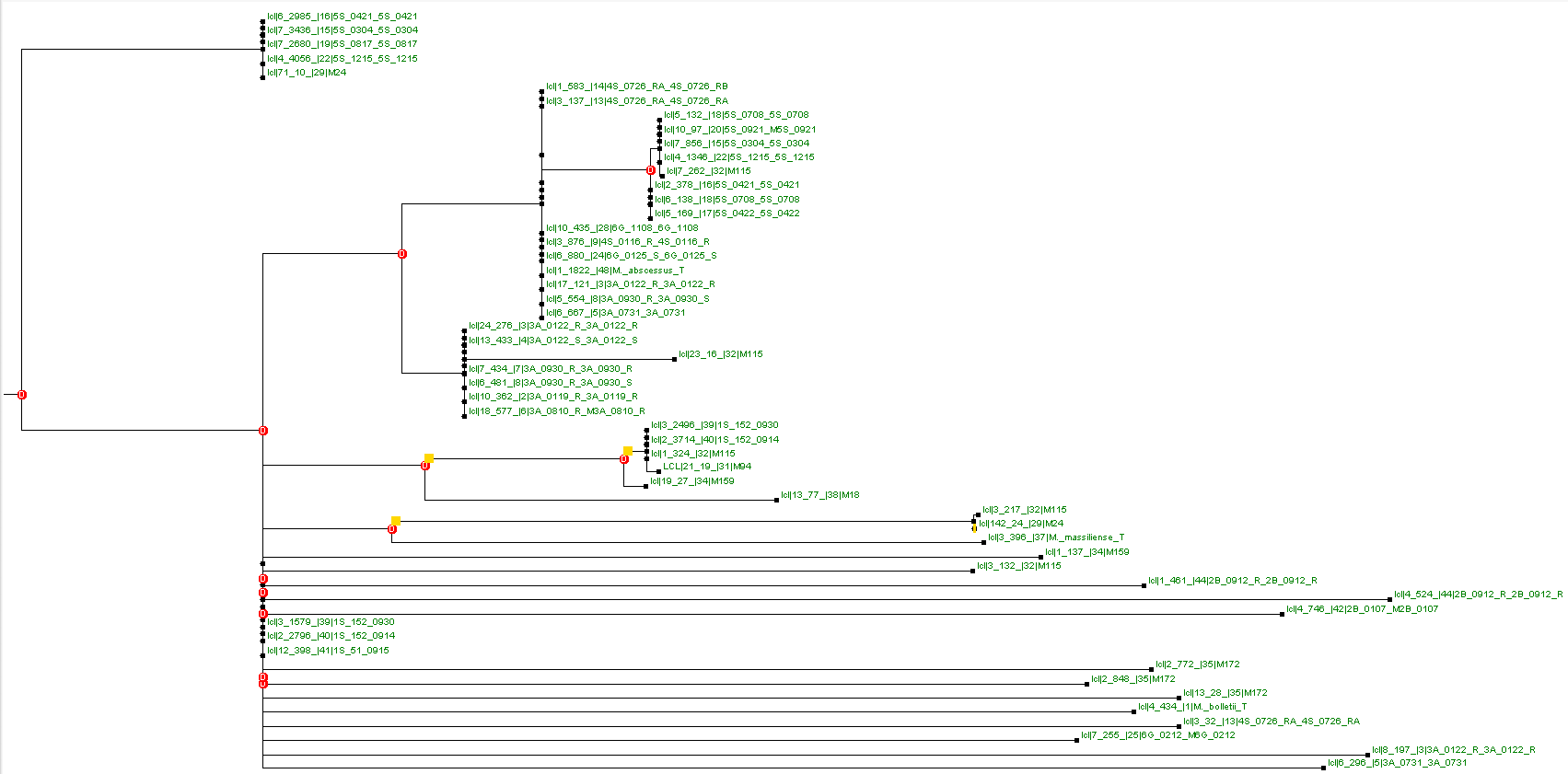

Supplement: Additional file 3 — The reconstructed trees for HGT events. Each tree contains one to six HGT events. The yellow squares represent the HGT event. [file 1745-6150-9-19-S3.zip › Tree_70.png]

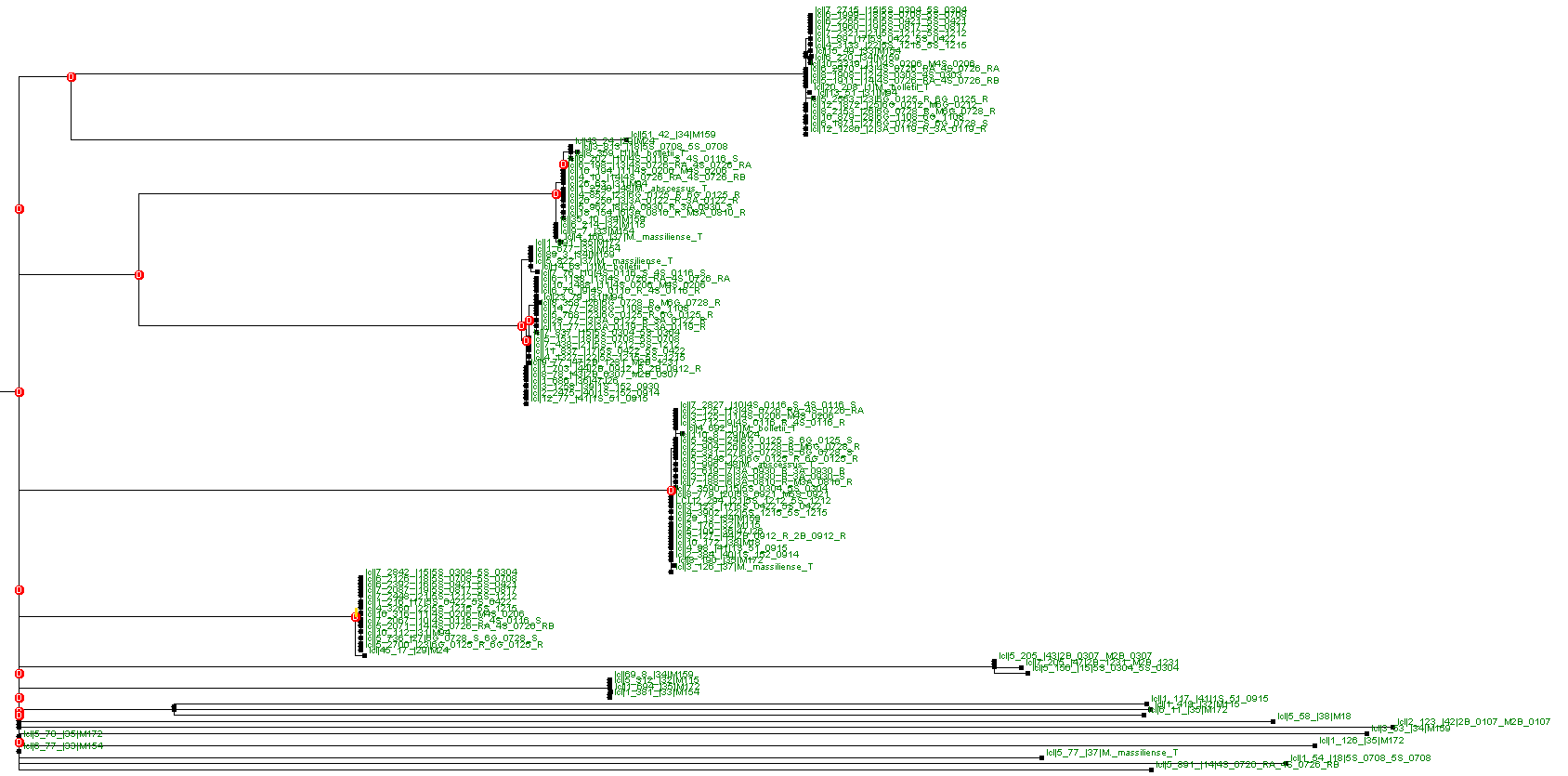

Supplement: Additional file 3 — The reconstructed trees for HGT events. Each tree contains one to six HGT events. The yellow squares represent the HGT event. [file 1745-6150-9-19-S3.zip › Tree_71.png]

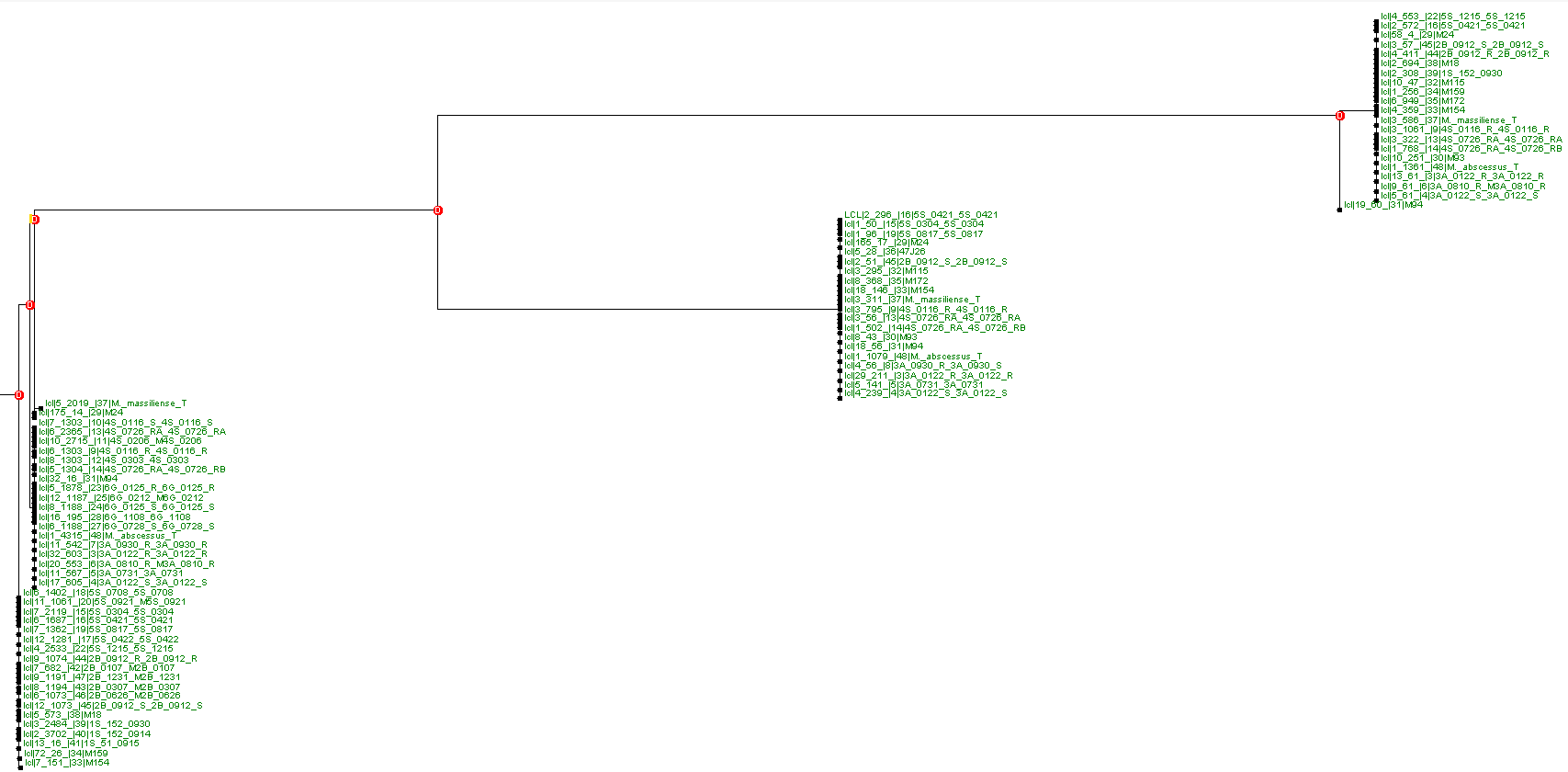

Supplement: Additional file 3 — The reconstructed trees for HGT events. Each tree contains one to six HGT events. The yellow squares represent the HGT event. [file 1745-6150-9-19-S3.zip › Tree_72.png]

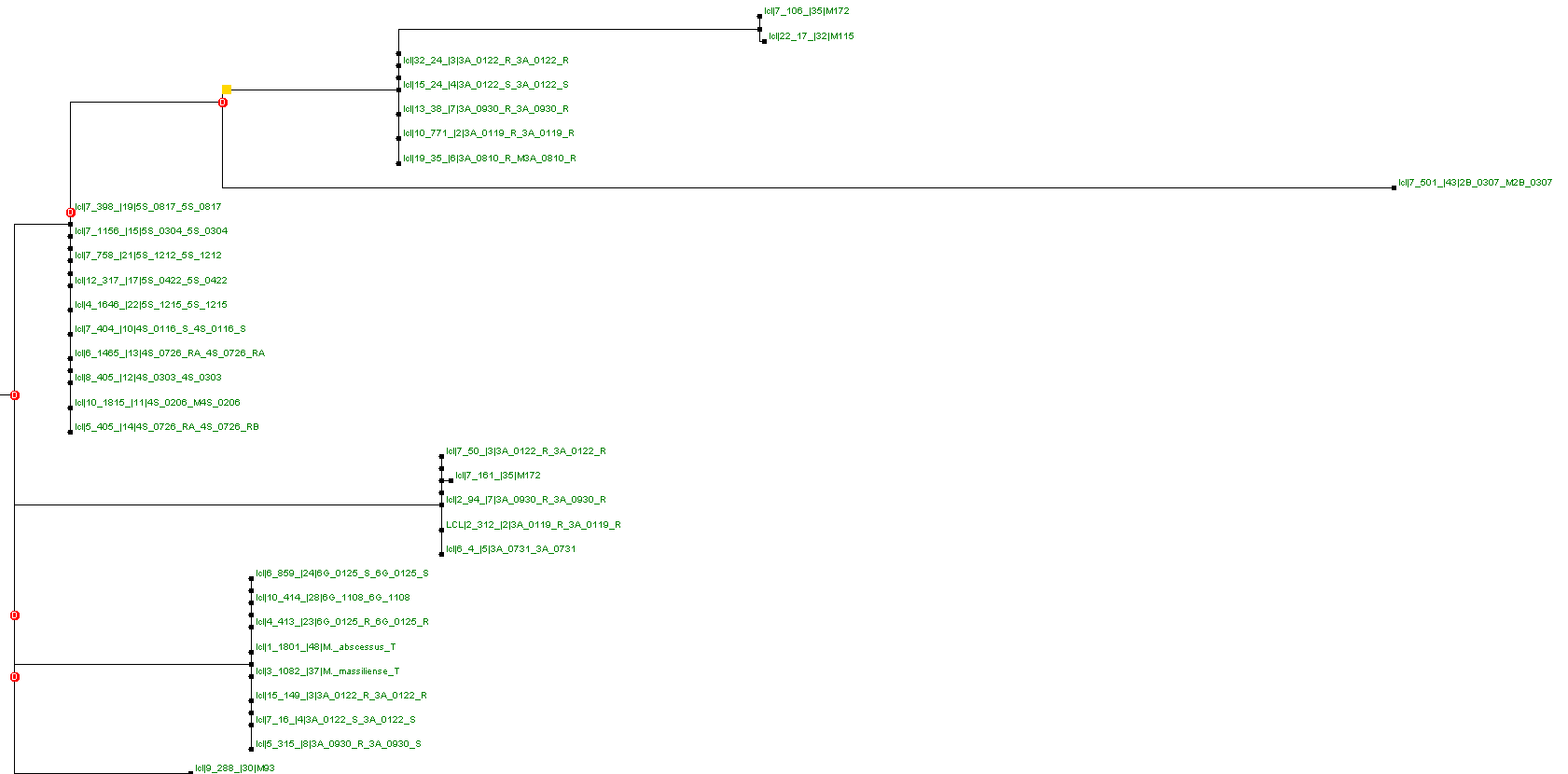

Supplement: Additional file 3 — The reconstructed trees for HGT events. Each tree contains one to six HGT events. The yellow squares represent the HGT event. [file 1745-6150-9-19-S3.zip › Tree_73.png]

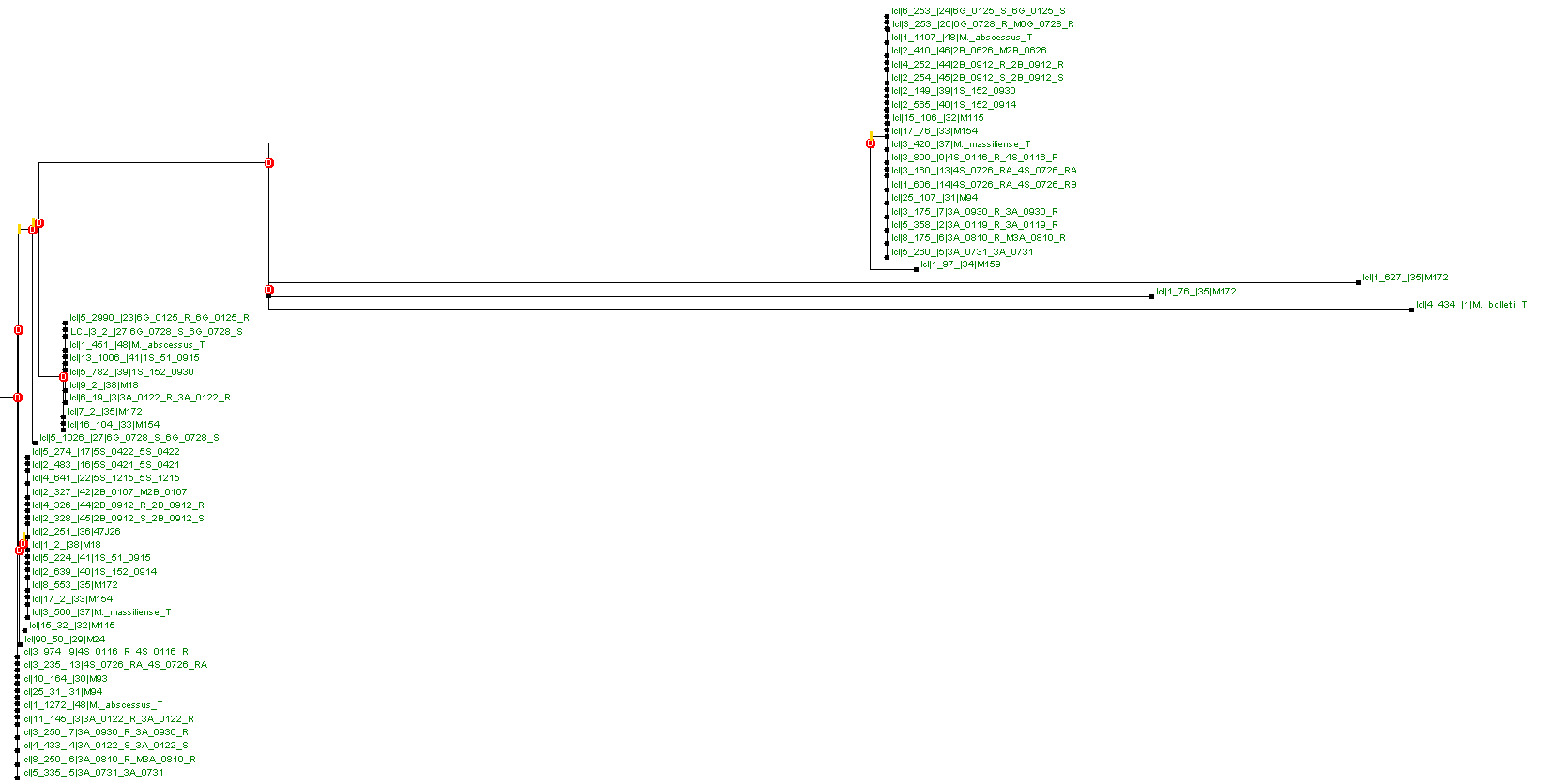

Supplement: Additional file 3 — The reconstructed trees for HGT events. Each tree contains one to six HGT events. The yellow squares represent the HGT event. [file 1745-6150-9-19-S3.zip › Tree_74.png]

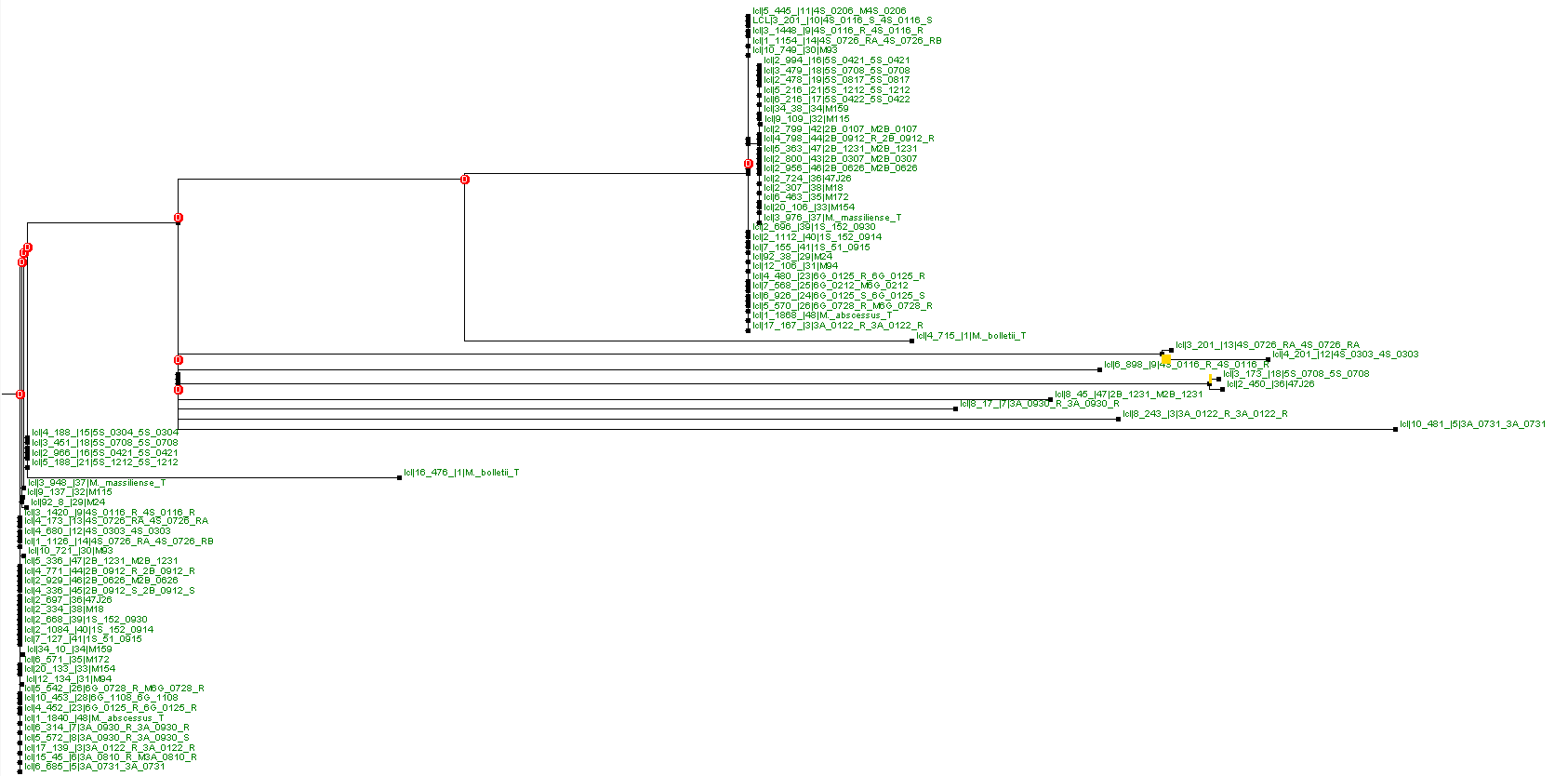

Supplement: Additional file 3 — The reconstructed trees for HGT events. Each tree contains one to six HGT events. The yellow squares represent the HGT event. [file 1745-6150-9-19-S3.zip › Tree_75.png]

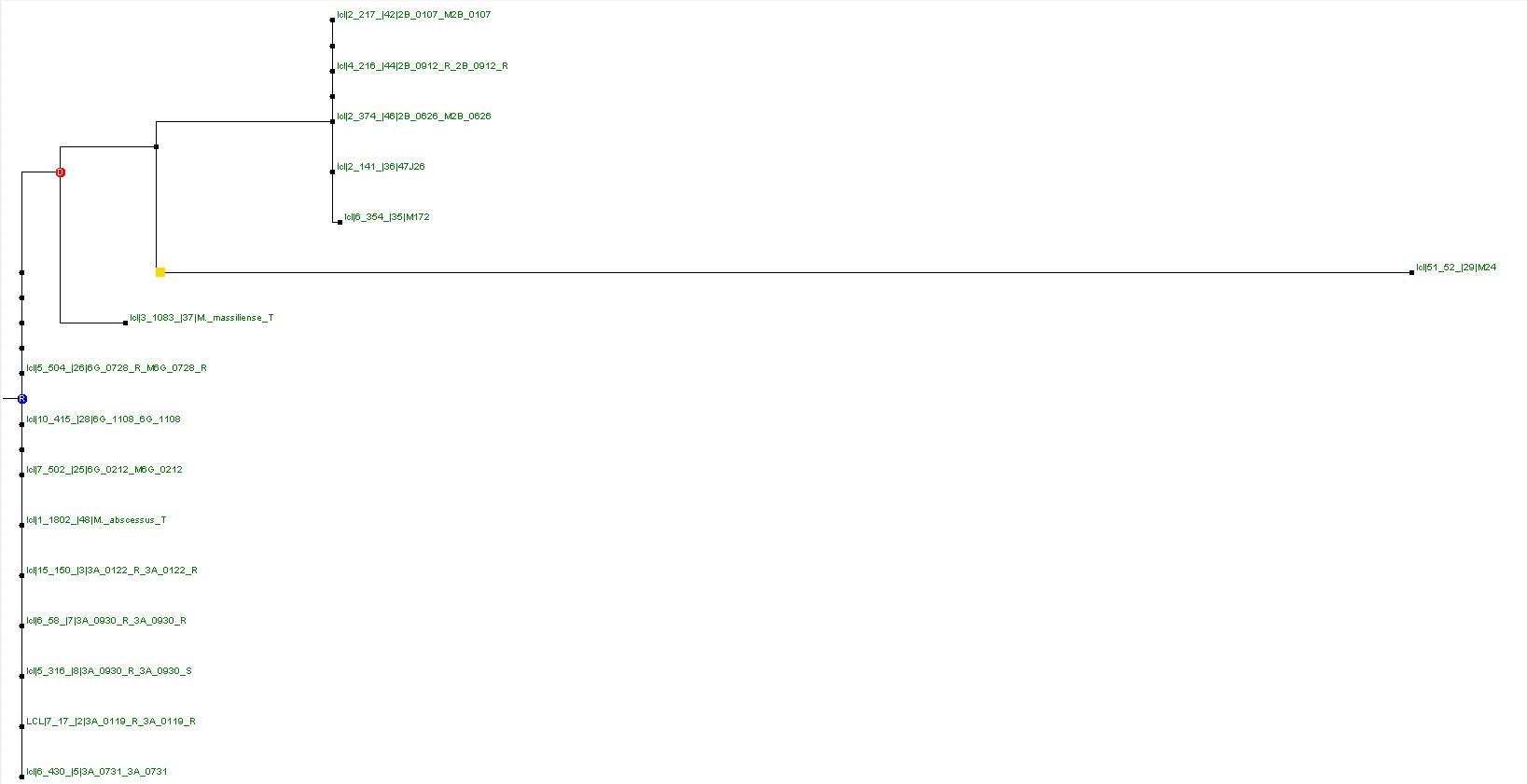

Supplement: Additional file 3 — The reconstructed trees for HGT events. Each tree contains one to six HGT events. The yellow squares represent the HGT event. [file 1745-6150-9-19-S3.zip › Tree_8.png]

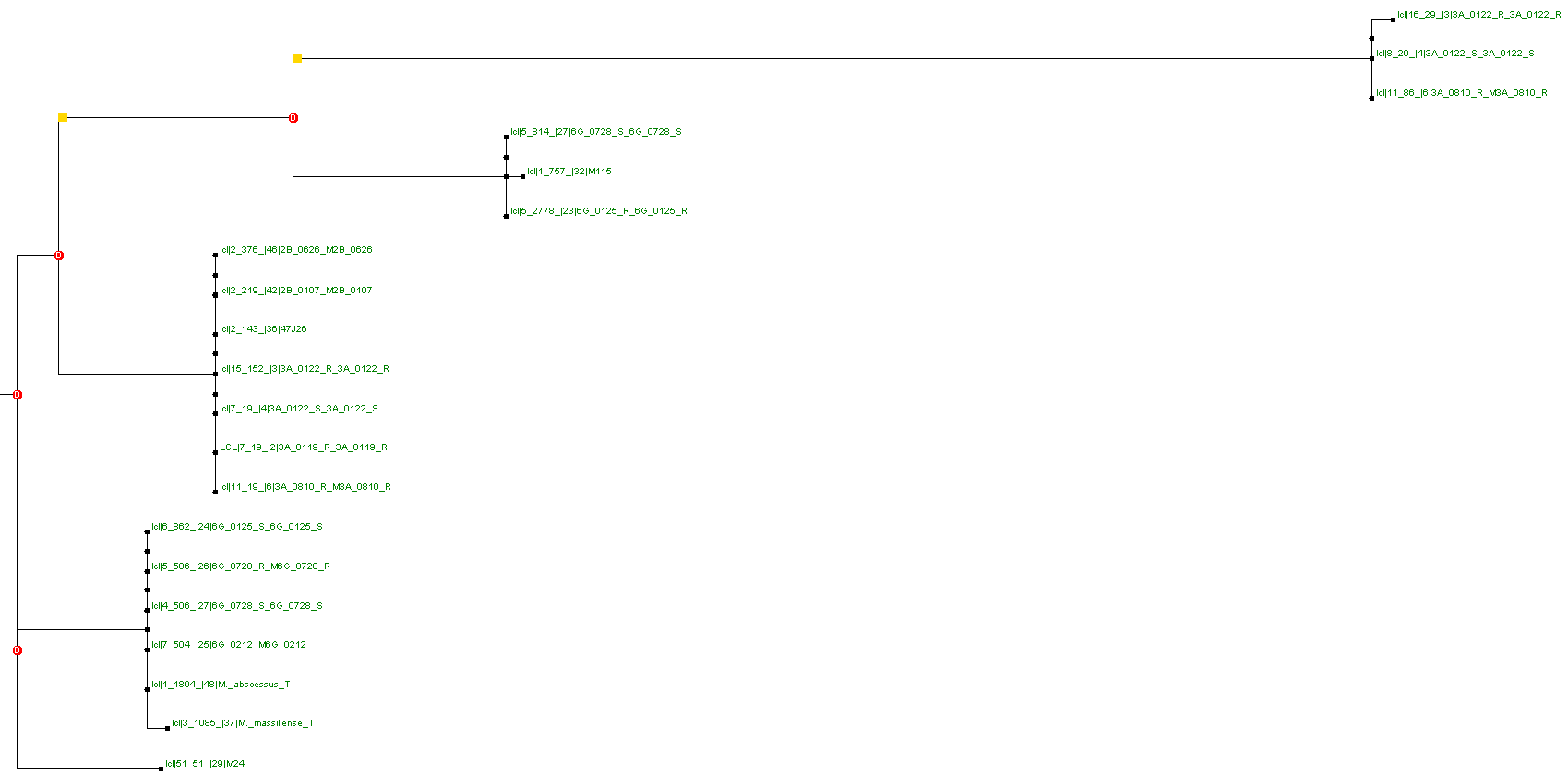

Supplement: Additional file 3 — The reconstructed trees for HGT events. Each tree contains one to six HGT events. The yellow squares represent the HGT event. [file 1745-6150-9-19-S3.zip › Tree_9.png]
